# Outlasting the Heat: Collapse of Herbivorous Fish Control of Invasive Algae During Marine Heatwaves

**DOI:** 10.1111/gcb.70438

**Published:** 2025-08-20

**Authors:** Jeroen Brijs, Leon L. Tran, Chloe Moore, Taylor Souza, Mathias Schakmann, Katherine Grellman, Jacob L. Johansen

**Affiliations:** ^1^ Hawaiʻi Institute of Marine Biology University of Hawaiʻi at Mānoa Kāneʻohe Hawaiʻi USA; ^2^ Institute of Zoology University of Innsbruck Innsbruck Austria; ^3^ Hopkins Marine Station, Stanford Oceans Stanford Doerr School of Sustainability Pacific Grove California USA

**Keywords:** avoidance, climate change, condition, foraging, herbivory, metabolism, preference, warmingalgal removal

## Abstract

Marine heatwaves (MHWs), coral bleaching, and chronic local stressors such as eutrophication are accelerating regime shifts from coral‐ to algae‐dominated reefs, increasingly favoring the proliferation of invasive, fast‐growing, and often more grazing‐resistant turf and macroalgae. A central tenet of global reef management strategies is that herbivorous fishes can sustain critical top‐down control of algal proliferation as oceans warm. Here, we challenge this tenet by experimentally evaluating, under controlled laboratory conditions, whether herbivorous coral reef fishes across three key functional groups—browser ( *Naso lituratus* ), grazer ( *Acanthurus triostegus* ), and scraper (*Chlorurus spilurus*)—can maintain effective algal control across present‐day (24.0°C–27.5°C) temperatures and into projected MHWs (31°C). We assessed (1) whether individuals evacuated thermally stressed conditions, effectively abandoning algal control, and (2) for those that remained, whether they could meet elevated energetic demands by foraging *ad libitum* on a mixture of *Caulerpa* spp.—a rapidly spreading and archetypal group of invasive algae in the Indo‐Pacific. All species gained body mass while foraging exclusively on these algae during winter and summer (~0.18%–0.62% per day). However, despite remaining in thermally stressed conditions and maintaining stable foraging rates, all species experienced consistent body mass declines (~0.41%–1.62% per day) under MHW exposure. This precipitous decline in body mass was driven by ~54%–60% increases in basal energetic demands without corresponding increases in food intake. Survival estimates based on body mass loss ranged from ~20–81 days, which is substantially shorter than the projected ~126–152‐day average duration of future MHWs. Our findings reveal that while short‐term algal control may persist during thermal stress, prolonged exposure appears to erode herbivore physiological condition, effectively undermining top‐down control of some algal types. Consequently, as ocean warming intensifies, herbivore protection strategies may become increasingly less effective at staving off algae proliferation and dominance in threatened reef ecosystems.

## Graphical abstract

Marine heatwaves (MHWs) and eutrophication are driving coral reefs toward algal dominance. Herbivorous fishes are thought to help slow this shift by feeding on fast‐growing and often invasive, herbivore‐resistant algae, but our experiments show their ability to do so declines under prolonged thermal stress. Although fish continued feeding at peak MHW temperatures, they lost body mass due to a mismatch between energy intake and rising energy demands. This suggests that extended MHWs may undermine the long‐term capacity of herbivores to control some algae, weakening a key reef management strategy as climate change accelerates.

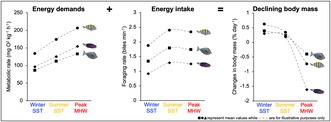

## 1 Introduction

Coral reefs rank among the most biologically diverse marine ecosystems, underpinning critical ecosystem services such as food provision, economic income, and shoreline protection for over half a billion coastal inhabitants worldwide (Hoegh‐Guldberg et al. 2017). Despite their immense ecological and socioeconomic value, coral reefs are currently undergoing unprecedented transformations driven by unabated ocean warming since the 1970s, compounded by increases in the frequency, intensity, and duration of marine heatwaves (MHWs) (Fox‐Kemper et al. 2021; Frölicher et al. 2018; Hobday et al. 2016; Oliver et al. 2018; Smale et al. 2019). Indeed, recent MHWs spanning regions from the Persian/Arabian Gulf (Burt et al. 2019; Genevier et al. 2019), the Indo‐Pacific (Harrison et al. 2019; Hughes et al. 2018), to the Gulf of Mexico and Caribbean (Bove et al. 2022; Donovan et al. 2021; Hobday et al. 2023) have caused widespread coral bleaching and mortality, followed by disease outbreaks and habitat collapse in the worst cases (Eakin et al. 2010; Heron et al. 2016; Hughes et al. 2018).

These forceful agents of disturbance are exacerbated by chronic local anthropogenic stressors, notably eutrophication and overfishing, significantly undermining coral reef resilience—that is, the capacity of coral reefs to absorb shocks, regenerate after disturbances, and resist regime shifts (Bellwood et al. 2004; Bove et al. 2022; Donovan et al. 2021; Graham et al. 2013; Smale et al. 2019). A prominent ecological outcome of these compounded stressors is the rapid proliferation of turf and macroalgae (Anton et al. 2020; Bellwood et al. 2004; Donovan et al. 2021; Hughes et al. 2007), including invasive species, which can exploit nutrient‐rich conditions to rapidly colonize degraded reefs (Arasamuthu et al. 2023; Fernandez and Cortes 2005; Kružić et al. 2008; Lapointe et al. 2005; Lapointe and Bedford 2010; Manikandan and Ravindran 2017; Pérez‐Estrada et al. 2013). These problematic algae not only outcompete corals for critical resources such as space, nutrients, and light, but also inhibit coral recovery through mechanisms such as smothering and enhancing harmful microbial activity (Anton et al. 2020; Bellwood et al. 2004; J. E. Smith et al. 2006). Consequently, uncontrolled algal proliferation reinforces regime shifts away from coral‐dominated states, altering community structure and severely compromising reef ecosystem resilience and productivity (Bellwood et al. 2004; Donovan et al. 2021; Graham et al. 2015; Hughes et al. 2007; Stuart‐Smith et al. 2018).

To mitigate algal proliferation and maintain coral dominance, the protection of herbivorous fish populations has been highlighted as a critical management strategy since they facilitate the settlement, growth, and survival of corals and coral recruits via top‐down algal control (Bellwood et al. 2004; Graham et al. 2013; Hughes et al. 2007). Herbivores are broadly categorized into functional groups—browsers, grazers, and scrapers—that collectively provide complementary roles in limiting algal overgrowth (Bellwood et al. 2004; Clements and Choat 2018; Green and Bellwood 2009; Sheppard et al. 2017). Browsers primarily consume fleshy, fast‐growing macroalgae that overgrow established coral colonies (Sheppard et al. 2017). Grazers feed on epilithic algal turf that smothers coral settlement substrates (Sheppard et al. 2017). Scrapers target protein‐rich epilithic, endolithic, and epiphytic microscopic phototrophs while taking bites off algae or the reef matrix (Clements and Choat 2018). These functional groups are often seen as the last bastion against uncontrolled algal growth; however, their capacity to fulfill this role may be compromised under climate change, particularly during MHWs, when both herbivore performance and algal composition are likely to shift.

The efficacy of herbivorous fishes as algal controllers in future warming scenarios may not only hinge on their resilience to the same thermal conditions that cause corals to bleach and die, but also on their capacity to sustain themselves while foraging on increasingly dominant and fast‐growing problematic algae often known to possess chemical and/or structural defenses against herbivory. Since coral reef fishes are exclusively ectothermic, all aspects of their metabolic demands and fitness are dictated by ambient temperature (Barneche et al. 2014; Pratchett et al. 2015). Based on global mean projections for most marine ectotherms, a 10°C rise in temperature is expected to induce a ~2–3‐fold increase in the metabolic demands of maintaining homeostasis (standard metabolic rate, SMR), defined as a temperature coefficient (*Q*_10_) of 2 to 3 (Seebacher et al. 2015). Therefore, temperature increases of 3°C–4°C during a MHW should, if not lethal (Genin et al. 2020; Pearce and Feng 2013), increase SMR and daily metabolic demand by ~23% to 55% (Seebacher et al. 2015). However, as temperature increases, the maximum capacity for oxygen delivery (maximum metabolic rate, MMR) may plateau or fall when temperatures exceed species‐specific thermal optima (Gardiner et al. 2010; Habary et al. 2017; Johansen and Jones 2011; Nilsson et al. 2009; Rummer et al. 2014) resulting in a loss of excess oxygen availability (aerobic scope, AS) to support activities essential for biological fitness (Pörtner and Farrell 2008; Pörtner and Peck 2010; Pratchett et al. 2015). Indeed, temperatures just 2°C–4°C above annual summer maxima have been demonstrated to cause reductions in swimming ability (Johansen et al. 2014; Johansen and Jones 2011), growth potential (Munday et al. 2008; Zarco‐Perello et al. 2012), reproductive output (Donelson et al. 2010; Pankhurst and Munday 2011) and survivorship (Nilsson et al. 2009) in a wide range of tropical coral reef fishes.

Thus, despite an expectation of high thermal sensitivity, coral reef fishes must be able to overcome thermally induced elevations in metabolic demand if they are to survive and thrive. This may be achieved by either fleeing to cooler waters (e.g., by moving across thermoclines or migrating to more thermally optimal habitats, effectively undermining top‐down algal control; Feary et al. 2014; Habary et al. 2017), increasing energy acquisition (e.g., by increasing foraging rates; Afeworki et al. 2013; Smith 2008), and/or reducing energy expenditure (e.g., by reducing activity; Johansen et al. 2014). However, herbivorous fishes are thought to survive on a low energy diet and are known to forage almost incessantly (e.g., > 90% of the time for some non‐territorial species of herbivores; Bruggemann, Begeman, et al. 1994; Bruggemann, Van Oppen, and Breeman 1994; Polunin et al. 1995) to fulfill energetic requirements under present day thermal conditions. Consequently, the ability of herbivores to either increase energy acquisition or reduce expenditure in response to rising energetic demands in a warming ocean appears limited. Worryingly, this limitation may be further exacerbated by the increasing spread of invasive macroalgae, such as *Caulerpa* spp., which can produce chemical deterrents (e.g., caulerpin and caulerpicin) that reduce palatability and impede effective grazing (Chisholm et al. 1996; Ravaglioli et al. 2022; Williams et al. 1985). Consequently, physiological and ecological constraints stemming from both ocean warming and the increasing proliferation of problematic algal types have the potential to significantly undermine the effectiveness of herbivore fish grazing for top‐down algal control.

This study aims to experimentally evaluate the potential of herbivorous coral reef fishes, across key functional groups (i.e., browsers, grazers, and scrapers), to effectively control an archetypical invasive and problematic group of algae (*Caulerpa* spp.) for tropical coral reefs under conditions of rising ocean temperatures characteristic of MHWs. We specifically investigate whether representatives of all three herbivore functional groups preferentially avoid thermal stress; that is—can their functional roles be relied upon during MHWs? We then characterize their relative capacity to meet elevated metabolic demands through *ad libitum* foraging on a mix of invasive *Caulerpa* algae under present‐day (24.0°C–27.5°C) and MHW (31°C) scenarios. We hypothesize that herbivorous coral reef fishes will selectively avoid thermal stress when given the opportunity, causing a precipitous decline in top‐down algal control during MHWs. For fishes that cannot, or do not, avoid elevated temperatures, we hypothesize that because these fishes already forage continuously throughout the day, they will be unable to further increase algal foraging rates to support thermally induced elevations in energetic demands, which when combined with the reduced palatability of *Caulerpa* spp. will result in a growing mismatch in energy acquisition and demand across temperatures.

## 2 Materials and Methods

### 2.1 Study Species, Collection and Holding Conditions

Representative herbivorous fishes spanning three functional groups (browser: *Naso lituratus* ; grazer: *Acanthurus triostegus* ; scraper: *Chlorurus spilurus*) were selected based on their ecological importance and high abundance on shallow water reefs throughout the equatorial Pacific (Randall 1998; Sheppard et al. 2017; Streit and Bellwood 2023). These species all help control algal overgrowth on coral reefs, with browsers and grazers typically gaining most of their nutrition directly from algal digestion (Nalley et al. 2022; Randall 1961), whereas scrapers also gain a proportion of their nutrition from protein‐rich epilithic, endolithic, and epiphytic microscopic phototrophs (Clements and Choat 2018). A total of 222 fish were collected (i.e., comprising of 66 *N. lituratus* , 76 *A. triostegus* , and 80 *C. spilurus* ) with hand nets and barrier nets by snorkelers and/or scuba divers from patch reefs along the eastern coast of Oʻahu, Hawaiʻi during the winter and summer of 2021 and 2022 (i.e., January–March, 24.0°C ± 0.5°C, and July–September, 27.5°C ± 0.5°C, respectively, data are reported as mean ±95% CI unless otherwise stated). Fish were then transported to the Johansen Fish Resilience Laboratory at Moku o Loʻe in Kāneʻohe Bay and kept in groups of 4–6 individuals per large conical holding tank (height: 86.0 cm; diameter: 82.5 cm; volume: 260 L; stocking density: ~1 g L^−1^), which contained sections of PVC pipe for shelter. Tanks were supplied with flow‐through, filtered, aerated, and UV‐sterilized seawater and subjected to a 12 h:12 h light: dark photoperiod. Water within the holding tanks was maintained at 24.0°C ± 0.1°C and 27.5°C ± 0.1°C during the winter and summer, respectively, reflecting mean seasonal sea surface temperatures (SST) of Kāneʻohe Bay (data retrieved online from tidesandcurrents.noaa.gov; Moku o Loʻe weather station; ID: 1612480; depth = 1 m; 21.433° N, 157.786° W). Water temperature within each tank was regulated using temperature control relays (WH1436, Willhi, China) that activated when the water temperatures were not within the desired temperature range to either heat or cool the tank. Heating within each tank was achieved via an 800 W aquarium heater (TH‐08005, Finnex Inc., USA), whereas cooling was achieved via a submersible pump (D08V045CD, Kedsum, China) that circulated water from within the tank at a rate of 985 L h^−1^ through an aluminum coil submerged in a 95 L external reservoir held at 5°C by a water chiller (ECO‐1 1/2HP, Ecoplus, USA).

Fish were acclimated to laboratory conditions for 2 weeks prior to experimentation and were provided *ad libitum* with a daily surplus of freshly collected algal matrix, composed primarily of *Caulerpa verticillata* (~90%) and *Caulerpa sertularioides* (~10%). Thin mats of this matrix were abundant at study sites surrounding Moku o Loʻe (Abbot and Huisman 2004; Carlton and Eldredge 2009; Hodgson et al. 2004), where both species occurred naturally intertwined, creating a turf matrix of fine siphonous algae measuring 2–6 cm in height (Souza et al. 2025). This matrix was maintained intact during experiments to preserve its structural integrity and ecological relevance as a foraging substrate. Although the broader biogeographic status of these problematic algae across the Indo‐Pacific remains uncertain, *Caulerpa* spp. are recognized for their high morphological plasticity, turf‐forming growth strategies, and capacity to spread laterally via extensive rhizome networks—traits that confer significant invasive potential, particularly under conditions of warming and eutrophication (Fernandez and Cortes 2005; Pérez‐Estrada et al. 2013; Ravaglioli et al. 2022; Williams et al. 1985). Despite not being preferred dietary items for most tropical coral reef herbivores, *Caulerpa* spp. were selected as the problematic algae of choice for this study due to their local abundance, rapid proliferation, and known capacity to disrupt coral reef ecosystems—making them an important and highly topical model for evaluating herbivore performance under climate‐driven reef degradation. Pilot trials confirmed that all focal herbivorous fish species readily foraged on the algal matrix and were able to maintain body condition under ambient temperatures, indicating both palatability and nutritional adequacy for the duration of the experiment (Souza et al. 2025). Importantly, the use of a standardized, non‐preferred, yet ecologically relevant food source minimized dietary variability among species, enabling a more controlled assessment of species‐specific physiological and behavioral responses to thermal stress.

Animal collection was conducted with the permission of the Department of Land and Natural Resources (Permit: SAP2022‐22), while all animal care and experimental procedures adhered to the ethical standards set by the Institutional Animal Care and Use Committee at the University of Hawaiʻi at Mānoa (Permit: 3200). In addition, a simple schematic overview of the experimental procedures is provided in the Supporting Information to aid interpretation of the methodology described below (Data S1).

### 2.2 MHW Simulation

In this study, a simulated MHW was defined as a discrete, prolonged warm water anomaly exceeding the climatological mean for at least 5 days (Hobday et al. 2016). Following the characterization of MHWs along the eastern coast of Oʻahu, Hawaiʻi from 1994 to 2020 (Tran and Johansen 2023), MHWs were simulated within large conical treatment tanks (height: 86.0 cm; diameter: 82.5 cm; volume: 260 L) by increasing water temperature ~0.9°C per day from mean summer SST (27.5°C ± 0.1°C) to a peak (31.0°C ± 0.1°C) over 4 days. This peak temperature was then maintained for an additional 3 days, resulting in a total MHW exposure duration of 7 days. These resulting thermal regimes closely reflected the mean duration (days), maximum heating rate (°C day^−1^), and maximum intensity (°C) for MHWs detected on collection sites for this study, ensuring that MHW simulations closely mirrored field conditions (Tran and Johansen 2023).

### 2.3 Assessment of MHW Avoidance Behaviour

MHW avoidance behaviour was assessed for each of the three representative species by randomly assigning individuals to either a “control” ( *N. lituratus* : *n* = 12, 37.9 ± 21.2 g; *A. triostegus* : *n* = 12, 34.8 ± 19.1 g; *C. spilurus* : *n* = 12, 32.1 ± 16.9 g) or “treatment” group ( *N. lituratus* : *n* = 12, 36.6 ± 13.1 g; *A. triostegus* : *n* = 12, 29.7 ± 11.0 g; *C. spilurus* : *n* = 12, 26.4 ± 10.3 g). Control fish were housed individually in one of four large conical treatment tanks, maintained at mean summer SST for a total of 7 days without exposure to a simulated MHW. In contrast, treatment fish were housed under identical conditions in a separate set of four identical tanks but were exposed to a simulated MHW for a total of 7 days (see *MHW simulation* for more details). Control and treatment fish were introduced into their respective tanks in a staggered fashion to ensure that all individuals experienced a 7‐day exposure period prior to subsequent analyses.

MHW avoidance behaviour was assessed using a two‐chamber shuttle‐box system that consisted of two identical cylindrical chambers made from white PVC walls and a translucent white Plexiglass bottom (diameter: 36 cm; height: 25 cm). The two chambers were joined with an opening between them (diameter: 5 cm; height: 10 cm) to allow fish to swim freely between the chambers. Both chambers contained a section of PVC pipe for shelter and a surplus supply of the previously mentioned algal matrix. One chamber was maintained at water temperatures representative of mean summer SST, while the other was held at the peak of a simulated MHW. To control for potential side bias, the chamber maintained at the peak of a simulated MHW was randomly assigned to either the left or right chamber for each trial. Water temperature was regulated by circulating water between each chamber and its corresponding, temperature‐controlled 37.8 L buffer tank at a rate of 300 L h^−1^ using an in‐line submersible pump (Universal 300, EHEIM, Germany). In turn, water temperature within each buffer tank was controlled using temperature control relays (WH1436, Willhi) that activated submersible pumps (D08V045CD, Kedsum) to keep the water temperature within ±0.1°C of the desired temperature. This resulted in water being pumped at a rate of 985 L h^−1^ through stainless steel coils that were either submerged in a 95 L external reservoir held at 5°C by a water chiller (ECO‐1 1/2HP) or at 45°C by 800 W aquarium heaters (TH‐08005, Finnex Inc.). Inlets and outlets in each chamber were constructed to ensure water flowed in a clockwise direction in one chamber and a counter‐clockwise direction in the other chamber, which prevented unwanted mixing of water between the two chambers via the opening. Temperatures within each chamber were continuously recorded (5 Hz) using in‐line thermocouple PT100 temperature sensors mounted along the inside chamber walls, which were connected to temperature readers (PR‐5714, PR Electronics, Denmark) and a PC. The entire setup was shielded with black plastic sheeting to protect fish from external visual stimuli while still maintaining a 12:12 light: dark photoperiod.

At the beginning of each trial, an individual from the “control” or “treatment” group was captured from its respective holding tank, weighed, and measured (to the nearest 0.1 g and 0.1 cm). The individual was then introduced into the chamber with the same water temperature as its holding tank (mean summer SST or peak of simulated MHW for individuals from the “control” or “treatment” group, respectively). Individuals were given at least 1 h to habituate to the system, during which they were visually monitored to confirm that they navigated between the chambers. This ensured that the fish were aware of the thermal gradient and could freely move toward cooler or warmer conditions as needed. Following habituation, the propensity of the individual to avoid the chamber maintained at the peak of a simulated MHW, while given the freedom to swim freely back and forth between the chambers, was tracked every second throughout the 22‐h experimental period (from ~09:00 to 07:00 HST). This was achieved using a mirror angled downward at 45° above the shuttle‐box system that allowed video monitoring of fish movements via a video camera (HDR‐XR100E, Sony, USA) positioned 4.75 m in the opposite direction. Infrared lights mounted below the translucent white Plexiglas bottoms were used to illuminate the chambers from below to create a detectable contrast between the fish and its surroundings. A PC video frame‐grabber (USB 2.0 DVD maker) then transmitted the video signal from the digital video camera to a PC with position analyzer software (LoliTrack, Loligo Systems, Denmark). Following each trial, the entire system was scrubbed and rinsed with clean seawater to eliminate the olfactory cues from the previous fish.

The proportion of time an individual spent avoiding the MHW chamber was calculated each hour (seconds spent in the chamber maintained at summer SST every hour/3600 s × 100) throughout the 22‐h experimental period. Since the herbivorous coral reef fishes investigated here are diurnal, the mean proportion of time an individual spent avoiding the MHW chamber was calculated during the day (from ~09:00 to 19:00 HST, when diurnal species are most active), and night (from ~19:00 to 07:00 HST, when diurnal species are least active), as well as during the entire protocol (from ~09:00 to 07:00 HST).

### 2.4 Assessment of Foraging Rates, Activity and Daily Change in Body Mass

Foraging rates, activity, and change in body mass of herbivorous coral reef fishes were experimentally evaluated at mean winter SST ( *N. lituratus* : *n* = 12, 70.7 ± 14.4 g; *A. triostegus* : *n* = 22, 53.8 ± 4.0 g; *C. spilurus* : *n* = 18, 42.2 ± 4.6 g), mean summer SST ( *N. lituratus* : *n* = 15, 45.1 ± 6.1 g; *A. triostegus* : *n* = 13, 51.4 ± 5.7 g; *C. spilurus* : *n* = 17, 30.1 ± 5.2 g), and at the peak of a simulated MHW during summer ( *N. lituratus* : *n* = 15, 47.9 ± 7.8 g; *A. triostegus* : *n* = 17, 50.3 ± 4.7 g; *C. spilurus* : *n* = 21, 36.5 ± 5.7 g). It should be noted that data on foraging rate, activity, and daily changes in body mass for *A. triostegus* were acquired (with permission) from Souza et al. (2025) to assess herbivore responses to MHWs within and across evolutionary and functional groups.

At the start of each trial, fish fasted for ~24 h were captured, weighed, and measured to the nearest 0.1 g and 0.1 cm before being individually assigned to one of eight large conical treatment tanks. These tanks were maintained at either 24.0°C ± 0.1°C (for the winter treatment group) or 27.5°C ± 0.1°C (for the summer and simulated MHW treatment groups). Fish were supplied daily with a surplus of the algal matrix (~5–10 g) attached to a PVC pipe with a rubber band at 08:30 HST, which was subsequently removed along with any floating or demersal food remnants at 16:30 HST. Fish were allowed exactly 3 days to resume “normal” foraging behavior before trials began, as pilot trials revealed that foraging rates and activity reached a stable plateau within that period of time. Fish were then video‐recorded throughout the day (between 09:30–09:40, 11:10–11:20, 12:50–13:00, 14:30–14:40, and 16:10–16:20 HST) using a 4 K surveillance camera system at 15 frames per second (N842 Series, Lorex Technology Inc., Canada). Fish in the winter and summer groups were video‐recorded over three consecutive days at their respective treatment temperatures. In contrast, fish in the simulated MHW treatment group were video‐recorded throughout the entire seven‐day MHW exposure (see *MHW simulation* for more details). At the end of each trial, fish were again fasted for ~24 h and remeasured to the nearest 0.1 g and 0.1 cm.

Video‐recordings were visually analyzed for foraging rates (bites min^−1^) by counting the number of bites an individual took from the algal matrix every minute during the video‐recordings. Direct measurement of algal biomass consumption was not feasible due to frequent fragmentation of the algal matrix during feeding, which resulted in a loss of material from the experimental system. Foraging activity (% time active) was visually analyzed by quantifying the proportion of time fish spent outside the shelter either actively feeding (i.e., taking bites from algae) or swimming (i.e., moving around the tank without feeding) each minute during the video recordings. Mean foraging rates and activity of herbivores were calculated from the three consecutive days of video‐recordings at mean winter and summer SSTs, as well as at the peak of the simulated MHW. Daily percent changes in body mass (% day^−1^) were determined by dividing the percent gain or loss of initial body mass by the number of days in the trial. Fish were fasted prior to both pre‐ and post‐trial measurements to minimize gut‐content variability (i.e., observed changes in body mass primarily reflect changes in tissue mass rather than gut contents) and/or to avoid the confounding effects of specific dynamic action (Secor 2009) on the whole‐animal aerobic metabolic rates calculated below.

### 2.5 Assessment of Whole‐Animal Aerobic Metabolic Rates

Whole‐animal aerobic metabolic rates of herbivorous coral reef fishes were determined on the same individuals used for the abovementioned assessment of foraging rates, activity, and daily change in body mass. Oxygen uptake rates (*Ṁ*O_2_, mg O_2_ kg^−1^ h^−1^) were obtained using best practices for intermittent‐flow respirometry (Clark et al. 2013; Roche et al. 2013; Svendsen et al. 2016) and used as a proxy for three estimates of whole‐animal aerobic metabolic rates—that is, MMR (the maximum aerobic metabolic rate that an individual can achieve; Norin and Clark 2016), SMR (the metabolic rate of a fasted and resting individual; Chabot et al. 2016), and AS (the scope for activities beyond basic maintenance calculated as the absolute difference between MMR and SMR; Clark et al. 2013). As above, fish were exposed to the simulated MHW for 7 days, followed by an additional day of fasting at peak MHW temperature prior to respirometry to ensure a post‐absorptive state necessary to estimate SMR (Chabot et al. 2016; Secor 2009). Note, data on SMR for *A. triostegus* were acquired with permission from Souza et al. (2025) to facilitate assessment of herbivore responses to MHWs within and across evolutionary and functional groups.

Intermittent‐flow respirometry was conducted in a temperature‐controlled room containing two experimental tanks (length: 97 cm, width: 53 cm, height: 37 cm) supplied with flow‐through, filtered, aerated, and UV‐sterilized seawater under a 12 h:12 h light: dark photoperiod. Water temperature within the experimental tanks was maintained within 0.1°C of winter, summer, and peak simulated MHW treatment temperatures. Each experimental tank contained four cylindrical acrylic respirometers that were either small (length: 14 cm, diameter: 7 cm, volume: 0.594 L) or large (length: 22 cm, diameter: 10 cm, volume: 1.600 L) depending on the size of the individual. Water was continuously circulated through each respirometer using an in‐line submersible pump (AD20P‐1230E, DollaTek, USA) within a recirculation loop to ensure a homogenous concentration of oxygen throughout the respirometer. Automated flush pumps intermittently refreshed the water within each respirometer according to the flush cycles that are described in detail below when determining MMR and SMR in the AquaResp software (v3.04, www.aquaresp.com). This ensured that oxygen levels in the respirometers always remained above 80% air saturation (Svendsen et al. 2016). The partial pressure of oxygen in the water within each respirometer was measured continuously at 1 Hz using a fiber optic oxygen sensor mounted in the recirculation loop where the flow is sufficient to ensure a rapid response time of the sensor. The optode was connected to a 4‐channel Firesting Optical Oxygen Meter (Pyro Science, Germany), which in turn was connected to a PC that logged the data. Oxygen measurements were automatically compensated for temperature (via a Pt100 temperature probe connected to the temperature port of the oxygen meter) and salinity (via manual input of water salinity into the Firesting logger software). Mass‐specific *Ṁ*O_2_ were then automatically calculated by the AquaResp software from the linear decline in the partial pressure of oxygen during the measurement period when the flush pumps were off.

Prior to eliciting MMR, the AquaResp software was set to MMR mode (“Wait” = 0.5 min, “Measure” = 1 min, and “Flush” = 2.5 min), the water level of the aforementioned treatment tanks holding the fish was lowered to 15 cm, and the shelters were removed. To induce MMR, a “chase protocol” was used (Clark et al. 2013; Norin and Clark 2016; Roche et al. 2013), whereby fish were individually chased by hand for 3 min, scooped into a fine mesh net, and kept out of the water for 1 min during which they were weighed and measured. Immediately following air exposure, fish were placed into respirometers and *Ṁ*O_2_ was measured over four intermittent flush cycles. Following this, SMR was determined by setting the AquaResp software to SMR mode (“Flush” = 5 min, “Wait” = 1 min, and “Measure” = 1 min). Fish remained in the respirometer to recover from the “chase protocol” and *Ṁ*O_2_ was measured for > 24 h. Pilot trials and previous studies on similar reef fish species revealed that this time period is more than sufficient for an individual to reach a stable resting state (Roche et al. 2013). Following the completion of the trial, fish were released back into the wild at the approximate location of capture. To account for background respiration, linear regression over time using measures of bacterial *Ṁ*O_2_ obtained from empty respirometers before and after each respirometry trial was subtracted from all *Ṁ*O_2_ measurements. To limit background respiration rates, all equipment was disinfected with a 1% bleach solution, thoroughly rinsed with freshwater, and allowed to dry before commencing further trials.

Following exclusion of individuals due to high background respiration (i.e., background respiration > 10% of SMR) or poor quality *Ṁ*O_2_ measurements (i.e., slopes with an *R* ^2^ < 0.95), a total of 41 *N. lituratus* (winter = 12, summer = 15, MHW = 14), 41 *A. triostegus* (winter = 18, summer = 12, MHW = 11), and 42 *C. spilurus* (winter = 12, summer = 15, MHW = 14) were used to determine whole‐animal aerobic metabolic rates. MMR was defined as the highest *Ṁ*O_2_ measured during the trial, while the 20% quantile method on *Ṁ*O_2_ measured during the recovery period yielded the most consistent SMR estimates based on visual inspection of *Ṁ*O_2_ and the cumulative variance of the mean lowest normal distribution (Chabot et al. 2016). AS was calculated as MMR minus SMR. Using mean SMR values, *Q*_10_ was calculated as follows: (*R*_2_/*R*_1_)^[10/(T2−T1)]^ for each species between winter and summer (R_1_: SMR_winter_; R_2_: SMR_summer_; T_1_: 24.0°C; T_2_: 27.5°C), as well as between summer and at the peak of a simulated MHW (R_1_: SMR_summer_; R_2_: SMR_peak of MHW_; T_1_: 27.5°C; T_2_: 31.0°C).

### 2.6 Statistical Analyses

All statistical analyses were performed using R version 4.2.3. Detailed descriptions of the statistical analyses used are provided in the Supporting Information (e.g., R packages used, data exploration, model fitting, model selection, model checking, and model inference; see Data [Link], [Link], [Link]).

Based on Akaike's Information Criterion (AIC), the most parsimonious linear mixed‐effects model for MHW avoidance behaviour (i.e., the proportion of time an individual spent avoiding the MHW chamber) included the fixed effects of time (“day” and “night”), treatment (“control” and “treatment”), species ( *N. lituratus* , *A. triostegus* and *C. spilurus* ), the interaction between time and species, and the interaction between treatment and species, while individual was included as a random effect (Data S2). To meet the assumptions of the model, MHW avoidance behaviour was transformed using a logit transformation. To determine whether or not a greater proportion of individuals from each species avoid the MHW chamber, exact binomial tests with exact Clopper‐Pearson 95% CI were subsequently performed using the total proportion of time an individual spent avoiding the MHW chamber during the 22‐h experimental period as the “success” category. MHW avoidance data for each species were pooled for the exact binomial tests based on the output of the linear mixed‐effects model.

Based on AIC, the most parsimonious linear regression models (for SMR, MMR, AS, foraging rates and daily changes in body mass) or beta regression model (for foraging activity) included the fixed effects of final body mass, treatment temperature (24.0°C, 27.5°C and 31.0°C), species, the interaction between final body mass and treatment temperature (for SMR and daily changes in body mass), the interaction between final body mass and species (for MMR, AS, foraging rate and foraging activity), and the interaction between treatment temperature and species (for MMR, AS and daily changes in body mass) (Data S3 and S4). Depending on the specific model, parameters either remained untransformed or were subjected to square root transformations (body mass and foraging rate), log transformations (body mass, SMR, MMR and AS), or an adjusted beta transformation (foraging activity) to meet model assumptions.

The main inferences from the most parsimonious models are reported throughout the text, while back‐transformed model means and confidence intervals overlay the raw data in the figures. Planned contrasts were performed on the estimated marginal means of the various models to investigate treatment temperature differences for average‐sized individuals of each species (mean body masses of 53, 52, and 35 g for *N. lituratus* , *A. triostegus* and *C. spilurus* , respectively). The resulting *p*‐values from the abovementioned analyses were subjected to False Discovery Rate (FDR) adjustment using the Benjamini‐Hochberg procedure to account for multiple testing (Benjamini and Hochberg 1995).

## 3 Results

### 3.1 MHW Avoidance Behaviour

**FIGURE 1:**
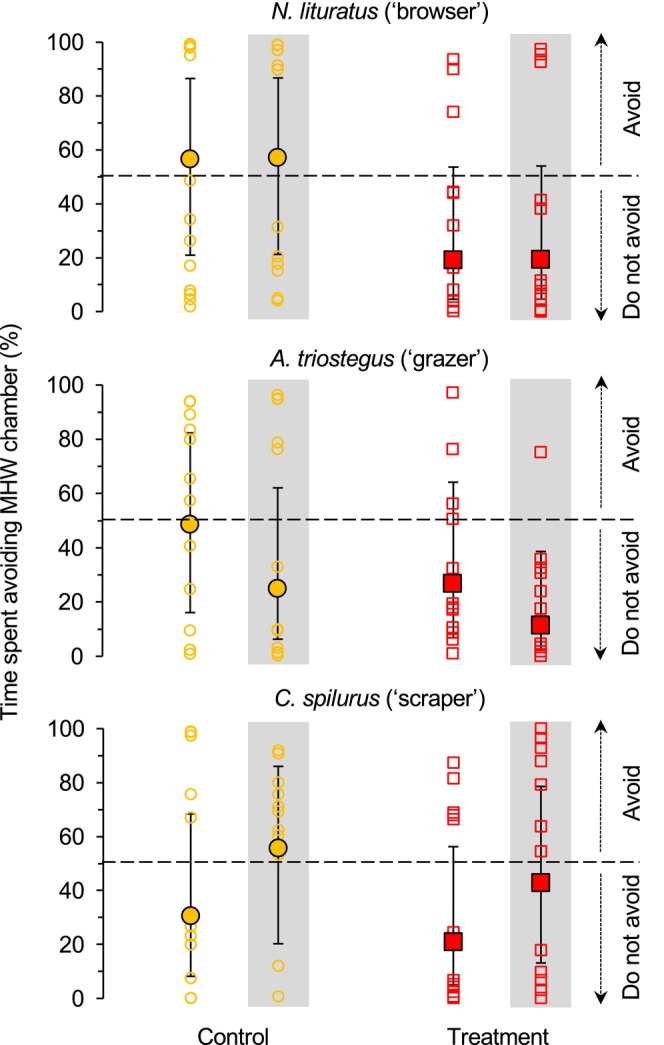
Heatwave avoidance behaviour of herbivorous coral reef fishes. The proportion of time that representative herbivorous coral reef fishes, spanning three functional groups, spent avoiding the chamber maintained at the peak of a simulated MHW (i.e., 31.0°C ± 0.1°C; back‐transformed estimated marginal means and 95% CI overlay the raw data, which are represented with filled and unfilled symbols, respectively). No significant differences (*p* < 0.05) were observed in the heatwave avoidance behaviour of any species with regards to time (“day” vs. “night” represented with no background and grey background, respectively) or treatment (“control/no prior exposure to a MHW” vs. “treatment/prior exposure to a MHW” represented with orange circles and red squares, respectively).

None of the herbivorous coral reef species preferentially avoided thermal stress (Figure 1A–C). Explicitly, the probability of an individual avoiding the MHW chamber did not significantly differ from random (50%) for *N. lituratus* (95% CI: 16%–55%, *p* = 0.152), *A. triostegus* (95% CI: 19%–59%, *p* = 0.308), or *C. spilurus* (95% CI: 19%–59%, *p* = 0.308). Similarly, the proportion of time spent avoiding the MHW chamber was unaffected by time of day (planned contrasts for “day” vs. “night”: *p* = 0.977, 0.218 and 0.218 for *N. lituratus* , *A. triostegus* , and *C. spilurus* , respectively) or treatment (planned contrasts for “control” vs. “treatment”: *p* = 0.218, 0.553 and 0.750 for *N. lituratus* , *A. triostegus* , and *C. spilurus* , respectively) (Figure 1A–C).

### 3.2 Whole‐Animal Aerobic Metabolic Rates

**FIGURE 2:**
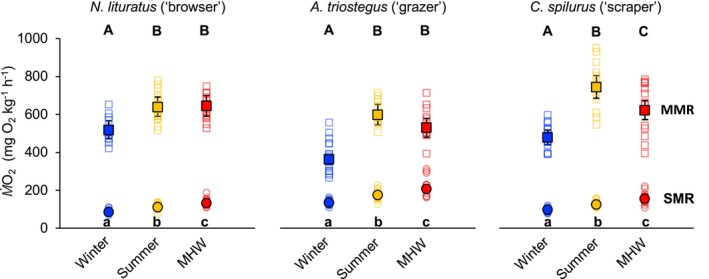
Whole‐animal aerobic metabolic rates of herbivorous coral reef fishes. Standard metabolic rate (SMR, circles) and maximum metabolic rate (MMR, squares) of representative herbivorous coral reef fishes, spanning three functional groups, during winter (24.0°C ± 0.1°C, blue symbols), summer (27.5°C ± 0.1°C, orange symbols), and at the peak of a simulated MHW (31.0°C ± 0.1°C, red symbols). Back‐transformed estimated marginal means and 95% CI overlay the raw data, which are represented with filled and unfilled symbols, respectively, while different letters represent the significant effects (*p <* 0.05) of temperature on SMR (lower case letters) and MMR (upper case letters) for each species.

Thermally‐induced elevations in energetic demand were conserved across the herbivorous coral reef species examined (Treatment temperature: *F*_2,116_ = 94.664, *p* < 0.001). Specifically, SMR increased by ~30% from winter to summer (*Q*_10_'s of 2.1 for all species, *p* < 0.001 for all planned contrasts), and by an additional ~19%–23% from summer to peak of MHW (*Q*_10_'s of 1.6–1.8 for all species, *p* < 0.001 for all planned contrasts) (Figure 2).

Conversely, species‐specific differences emerged in the maximum capacity for oxygen delivery, with responses varying between present day and MHW conditions (Interaction_species×treatment_: *F*_4,112_ = 4.050, *p* = 0.004). Specifically, MMR increased by ~23%–65% from winter to summer for all species (*p* ≤ 0.001 for all planned contrasts), but then it plateaued from summer to the peak of a MHW for *N. lituratus* and *A. triostegus* (*p* = 0.880 and 0.066, respectively) while it decreased by 16% for *C. spilurus* (*p* = 0.003) (Figure 2).

As a result of the thermally‐induced SMR and MMR responses, species‐specific differences in AS were also evident across temperatures (Interaction_species×treatment_: *F*_4,112_ = 4.142, *p* = 0.004). Specifically, AS increased from winter to summer for all species ( *N. lituratus* : Δ22%, *p* = 0.026; *A. triostegus* : Δ90%, *p* < 0.001; *C. spilurus* : Δ62%, *p* < 0.001), but then either plateaued ( *N. lituratus* : *p* = 0.727) or decreased from summer to the peak of a MHW ( *A. triostegus* : Δ28%, *p* < 0.001; *C. spilurus* : Δ27%, *p* < 0.001).

### 3.3 Foraging Rates and Activity Levels

**FIGURE 3:**
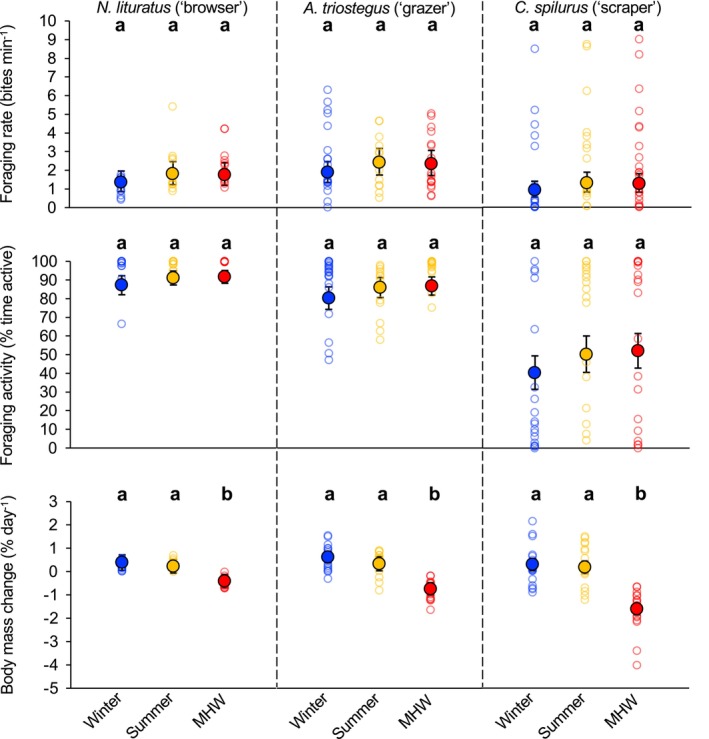
Foraging rates, activity and body mass changes of herbivorous coral reef fishes. Foraging rates (upper panel), foraging activity (middle panel), and daily body mass changes (lower panel) of representative herbivorous coral reef fishes, spanning three functional groups, during winter (24.0°C ± 0.1°C, blue circles), summer (27.5°C ± 0.1°C, orange circles), and at the peak of a simulated MHW (31.0°C ± 0.1°C red circles). Back‐transformed estimated marginal means and 95% CI overlay the raw data, which are represented with filled and unfilled symbols, respectively, while different letters represent the significant effects (*p <* 0.05) of temperature on each parameter for each species.

No significant thermally‐induced changes were evident in the foraging rate (Treatment temperature: *F*_2,142_ = 2.946, *p* = 0.055) or activity (Treatment temperature: *F*_2,142_ = 2.946, *p* = 0.065) of the herbivorous coral reef species examined. Specifically, mean foraging rates ranged between 1.3 and 1.8 bites min^−1^ for *N. lituratus* , 1.9–2.4 bites min^−1^ for *A. triostegus* , and 0.9–1.3 bites min^−1^ for *C. spilurus* across temperatures (*p* > 0.05 for all planned contrasts; Figure 3A). Similarly, mean proportion of time spent foraging ranged between 87% and 92% for *N. lituratus* , 80%–87% for *A. triostegus* , and 40%–52% for *C. spilurus* across temperatures (*p* > 0.05 for all planned contrasts; Figure 3B).

### 3.4 Daily Change in Body Mass

Body mass increased on average during winter and summer across all herbivorous coral reef species, while it declined sharply under MHW conditions, with the extent of the decline varying significantly among species (Interaction_species×treatment_: *F*_4,138_ = 2.640, *p* = 0.036; Figure 3C). Daily changes in body mass did not differ, respectively, between winter and summer for *N. lituratus* (0.39% ± 0.33% vs. 0.23% ± 0.29%, *p* = 0.518), *A. triostegus* (0.62% ± 0.23% vs. 0.34% ± 0.30%, *p* = 0.186) or *C. spilurus* (0.30% ± 0.26% vs. 0.18% ± 0.27%, *p* = 0.545). In contrast, body mass consistently declined under MHW conditions, with daily losses of 0.41% ± 0.28%, 0.74% ± 0.27%, and 1.62% ± 0.24% for *N. lituratus* [*p*_27.5°C vs. 31.0°C_ = 0.004], *A. triostegus* [*p*_27.5°C vs. 31.0°C_ < 0.001] and *C. spilurus* [*p*_27.5°C vs. 31.0°C_ < 0.001], respectively.

## 4 Discussion

The escalating interplay between global climatic disturbances, notably MHWs, and chronic local anthropogenic pressures such as eutrophication and overfishing is accelerating regime shifts from coral‐ to algal‐dominated reef states, favoring the expansion of problematic macroalgal species that undermine coral reef resilience and ecosystem functionality (Anton et al. 2020; Bellwood et al. 2004; Bove et al. 2022; Donovan et al. 2021; Hughes et al. 2007; Graham et al. 2013; Smale et al. 2019). This rapid and global degradation of coral reef ecosystems highlights the need for adaptive management strategies that bolster reef resilience, including the protection of herbivorous fishes and the ecological functions they support. A strong tenet in reef management is the idea that high herbivore abundance will ensure continued top‐down pressures on algal proliferation. However, our experimental findings reveal an important vulnerability in this tenet to manage problematic invasive algae. Despite remaining within thermally stressed conditions and maintaining stable foraging rates under a simulated MHW, herbivorous fishes across key functional groups (i.e., browsers, grazers, and scrapers) all experienced substantial energetic deficits, reflected by consistent declines in body mass. While short‐term control of problematic algae may persist during thermal stress, the progressive deterioration of herbivore physiological condition during prolonged exposure, when foraging solely on these rapidly proliferating algae, is likely to compromise both their survival and functional capacity. In the face of increasingly frequent, intense, and prolonged MHWs, compounded by nutrient enrichment and the rising prevalence of problematic algae, such stress‐induced energetic mismatches could progressively erode the efficacy of herbivory‐based reef management strategies.

A central pillar of global reef management strategies is the protection of herbivorous fishes, based on the assumption that they will remain in thermally stressed habitats and continue to provide top‐down algal control (Bellwood et al. 2004; Graham et al. 2013; Green and Bellwood 2009; Hughes et al. 2007; McLeod et al. 2009). However, many reef fishes are known to behaviorally thermoregulate, a form of phenotypic plasticity that enables individuals to mitigate thermal stress by selecting cooler environments, either through vertical movement or latitudinal range shifts (Feary et al. 2014; Habary et al. 2017; Nay et al. 2015, 2020, 2021). If also prevalent in herbivorous fishes, this behavior would directly challenge the efficacy of herbivore protection to maintain top‐down algal control in thermally stressed habitats. Interestingly, and contrary to our expectations, the herbivorous species examined here, spanning three functional groups and evolutionary lineages that diverged 17–62 million years ago (Bellwood et al. 2017; Siqueira et al. 2019; Sorenson et al. 2013), did not change their behavior to avoid thermally stressed conditions. Our findings may in fact highlight the extent of thermal site fidelity in the wild. On natural reefs, avoiding heat stress would likely require longer‐distance movements, often in the absence of clear thermal cues, and could come at significant energetic or ecological cost (Feary et al. 2014). This challenge is further compounded by the fact that marine heatwaves can substantially warm the entire water column to depths exceeding 100 m (Zhang et al. 2023), thereby reducing or eliminating the availability of thermal refugia (Bahr et al. 2017). Nonetheless, the structural complexity, thermal microhabitats, and species interactions found on natural reefs may influence behavioral responses in ways not captured here (Nay et al. 2015, 2020, 2021), and thus field‐based studies will be essential to verify the generality of a lacking thermal preference or movement behavior in herbivorous reef fishes, across more ecologically complex and variable reef environments.

A second criteria for effective top‐down algal control during periods of thermal stress depends on foraging rates and the ability of herbivores to survive on algae, typically viewed as a relatively energetically and nutritionally poor food source (Montgomery and Gerking 1980; Sterner and Hessen 1994). Specifically, fishes occupying thermally stressed habitats are expected to face significant elevations in energetic demand while their ability to meet these demands may become increasingly compromised (Pörtner and Farrell 2008; Pörtner and Peck 2010; Pratchett et al. 2015). Indeed, basal energetic demands across functional groups increased by ~54%–60% from winter to the peak of a MHW (*Q*_10_'s of ~1.9), consistent with thermally induced elevations of physiological rates in a range of other coral reef fish species (*Q*_10_'s of ~1.1–5.7; Gardiner et al. 2010; Habary et al. 2017; Johansen and Jones 2011; Nilsson et al. 2009; Rummer et al. 2014). Concurrently, aerobic scope of herbivorous coral reef fishes either plateaued or decreased by ~27%–28% from summer to the peak of a MHW, reflective of declining biological fitness (Gardiner et al. 2010; Habary et al. 2017; Johansen and Jones 2011; Nilsson et al. 2009; Pörtner and Peck 2010; Rummer et al. 2014). Declining biological fitness can take many forms including lowered reproductive output or growth, none of which were examined here. However, from a functional standpoint, all species retained adequate aerobic surplus to maintain foraging rates and activity within the temperatures examined here, highlighting that removal rates of problematic algae remained unaltered.

As energetic demands increase, so too does the need to feed. While basal energetic demands increased by ~54%–60% across the entire temperature range examined here, none of the study species were able to increase energy intake concomitantly when foraging on the *Caulerpa* algal matrix. While this resource was sufficient to support average daily body mass gains of ~0.18%–0.62% during winter and summer, all species experienced marked reductions in body mass during MHW exposure. Specifically, *N. lituratus* , *A. triostegus* , and *C. spilurus* exhibited average daily reductions in body mass of ~0.41%, 0.74%, and 1.62%, respectively. While exact mortality thresholds for body mass reductions in fish are conspicuously absent from the literature, starvation studies suggest death may occur after ~18%–33% body mass loss (Fishelson 1997; McCue 2010). Applying a conservative maximum loss of 33% (a level coincidentally defined as an extreme endpoint in many animal experiments; Talbot et al. 2020), individuals across the various functional groups may only survive for ~81, 45, and 20 days, respectively, when feeding solely on the *Caulerpa* algal matrix during MHWs. Given that the average duration of marine heatwaves under current high‐emission scenarios (SSP5–8.5) is projected to reach ~126–152 days by the end of the century (Collins et al. 2019; Frölicher et al. 2018; Laufkötter et al. 2020; Oliver et al. 2018), it is increasingly likely that some herbivorous coral reef fishes—and the critical ecosystem functions they support—may become locally untenable on reefs experiencing both prolonged thermal stress and a rising dominance of problematic macroalgae (Anton et al. 2020; Bellwood et al. 2004; Donovan et al. 2021; Hughes et al. 2007).

The inability of herbivorous fishes to maintain physiological condition when foraging exclusively on rapidly proliferating problematic algae during the simulated MHW likely reflects a combination of factors, including deviation from their natural dietary preferences (Bellwood et al. 2004; Clements and Choat 2018; Green and Bellwood 2009; Sheppard et al. 2017) and/or the presence of chemical or structural defenses that deter herbivory by reducing palatability (e.g., sesquiterpenoid compounds such as caulerpin and caulerpicin; Chisholm et al. 1996; Ravaglioli et al. 2022; Souza et al. 2025; Williams et al. 1985). Indeed, mean foraging rates on the problematic algae observed in the present study were lower than those previously reported in natural reef settings for *N. lituratus* (1.3–1.8 vs. 2.5–5.8 bites min^−1^; Rasher et al. 2013), *A. triostegus* (1.9–2.4 vs. 10–16 bites min^−1^; Basford et al. 2015), and *C. spilurus* (0.9–1.3 vs. 13–14 bites min^−1^; Bellwood and Choat 1990). This itself is not surprising given natural reef habitats (when not impacted by problematic algae) provide a broader range of food sources that align more fully with the dietary specializations of the individual species (Bellwood et al. 2004; Clements and Choat 2018; Green and Bellwood 2009; Sheppard et al. 2017). These specializations—shaped by the complex interplay of food quality (e.g., nutritional content and secondary metabolites), feeding mechanisms (e.g., jaw morphology and feeding behaviour), and digestive traits (e.g., gut length, stomach pH, enzyme activity, and fermentative capacity) (Clements et al. 2009)—may help, at least in part, to explain the species‐specific responses to thermal stress observed in the present study. For instance, *N. lituratus* , which specializes in macroalgae, experienced the smallest reductions in body mass, while *C. spilurus* , less reliant on algae and gaining much of its energy from protein‐rich epilithic, endolithic, and epiphytic microscopic phototrophs (Clements and Choat 2018), showed the greatest reductions. Nevertheless, despite differences in dietary specialization, all examined species across functional groups experienced significant energetic deficits during thermal stress, suggesting that none are likely to readily sustain themselves on reefs dominated by problematic algal species during prolonged periods of extreme warming.

A worthwhile future research direction will be to determine whether herbivorous fishes can meet the elevated energetic demands imposed by MHWs when foraging on their natural dietary items, or whether survival under thermal stress requires a shift toward more energy‐ and nutrient‐rich resources—such as benthic invertebrates (Shraim et al. 2017) or faeces from higher‐level consumers (Schiettekatte et al. 2020, 2023). Given the generally low energetic content of algae (Montgomery and Gerking 1980; Sterner and Hessen 1994) and the fact that many herbivorous species already allocate a substantial proportion of their daily activity budgets to foraging (Bruggemann, Begeman, et al. 1994; Bruggemann, Van Oppen, and Breeman 1994; Polunin et al. 1995), it seems unlikely that increasing foraging effort alone would be sufficient to offset future energetic demands. Indeed, > 50% declines in herbivore abundance and biomass during a nine‐month thermal stress event in the central Pacific Ocean (Magel et al. 2020) reinforce the likelihood that the ecological functions herbivores provide may be far more thermally sensitive than previously recognized. While such dietary shifts may enhance individual resilience by providing access to more energetically favorable resources, they would not mitigate the broader ecological challenge of managing rapid proliferation and dominance of problematic algal types during periods of extreme warming (Anton et al. 2020; Arasamuthu et al. 2023; Bellwood et al. 2004; Donovan et al. 2021; Fernandez and Cortes 2005; Hughes et al. 2007; Kružić et al. 2008; Lapointe et al. 2005; Lapointe and Bedford 2010; Manikandan and Ravindran 2017; Pérez‐Estrada et al. 2013).

Finally, while this study provides valuable insights into the short‐term responses of herbivorous fishes to summer MHW conditions, other factors may influence their longer‐term resilience and ecological function. For instance, metabolic and digestive processes can take weeks to fully acclimate to elevated temperatures (Sandblom et al. 2014; Seebacher et al. 2015), suggesting that the acute responses observed here may underestimate the potential for physiological compensation during prolonged heat exposure. Whether such acclimation enables herbivores to partially offset energetic shortfalls—and thus survive the 4–5 month durations projected for future MHWs (Frölicher et al. 2018; Oliver et al. 2018)—remains unknown. Furthermore, emerging evidence indicates that herbivorous fishes may be even more thermally sensitive during winter MHWs (Tran and Johansen 2023), a period that coincides with energetically demanding reproductive events in many reef species (Grandcourt et al. 2004; Randall 1961; Sadovy 1996; Schemmel and Friedlander 2017). Elevated temperatures during this time have been shown to reduce breeding rates, clutch size, egg quality, and hatching success (Donelson et al. 2010; Miller et al. 2015; Pankhurst and Munday 2011; Spinks et al. 2021), and to alter reproductive timing and range boundaries (Potts et al. 2014). These findings suggest that the biological consequences of ocean warming may disrupt ecological and reproductive processes across a greater portion of the year than previously recognized. Thus, to comprehensively assess the year‐round effectiveness of herbivore‐based reef management strategies, it is crucial to elucidate whether herbivorous coral reef fishes can meet elevated energetic demands under thermal stress when foraging on natural or mixed diets, and to understand how seasonal variation in thermal sensitivity, acclimation capacity, and trophic flexibility influence key ecosystem functions.

## 5 Conclusions

Overall, our findings reveal that while herbivorous coral reef fishes, spanning three distinct functional groups (i.e., browsers, grazers, and scrapers), may persist within thermally stressed habitats and maintain foraging activity short‐term, their longer‐term functional capacity to control rapidly proliferating algae such as *Caulerpa* spp. is likely to be severely compromised under future warming scenarios. Our data suggest that prolonged exposure to MHWs imposes energetic demands that these algae alone cannot satisfy, leading to progressive declines in herbivore condition and potential population attrition. These vulnerabilities, compounded by the increasing spread of problematic algal species and the intensifying impacts of local stressors, forewarn of a reduced capacity for reefs to recover from disturbance and resist regime shifts toward algal dominance. Sustaining coral reef resilience under unabated warming will therefore require management strategies that move beyond herbivore protection alone, incorporating proactive efforts to mitigate algal proliferation, preserve functional diversity, and reduce co‐occurring local stressors wherever possible.

## Author Contributions

**Jeroen Brijs:** conceptualization, data curation, formal analysis, investigation, methodology, validation, visualization, writing – original draft, writing – review and editing. **Leon L. Tran:** formal analysis, investigation, validation, visualization, writing – original draft, writing – review and editing. **Chloe Moore:** formal analysis, investigation, validation, visualization, writing – original draft, writing – review and editing. **Taylor Souza:** formal analysis, investigation, validation, visualization, writing – original draft, writing – review and editing. **Mathias Schakmann:** formal analysis, investigation, validation, visualization, writing – original draft, writing – review and editing. **Katherine Grellman:** formal analysis, investigation, validation, visualization, writing – original draft, writing – review and editing. **Jacob L. Johansen:** conceptualization, data curation, formal analysis, funding acquisition, investigation, methodology, project administration, resources, software, supervision, validation, visualization, writing – original draft, writing – review and editing.

## Conflicts of Interest

The authors declare no conflicts of interest.

## Supporting information


**Data S1:** Supporting Information 1.

**Data S2:** Supporting Information 2.

**Data S3:** Supporting Information 3.

**Data S4:** Supporting Information 4.

## Data Availability

The data that support the findings of this study are openly available in https://doi.org/10.6084/m9.figshare.28562525. Due to large file size, video recordings of fish behavior are available on request.
