# Supplementary material for: Outlasting the Heat: Collapse of Herbivorous Fish Control of Invasive Algae During Marine Heatwaves
Source: Glob Chang Biol. 2025 Aug 20;31(8):e70438. doi: 10.1111/gcb.70438 (PMC12365732; doi:10.1111/gcb.70438)
Supplement: Supplementary file 1 — Data S1: Supporting Information 1. [file GCB-31-e70438-s002.pdf]

Schematic of experimental methodology

1.

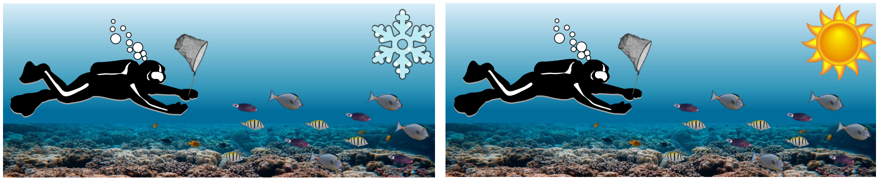

Fish collection & acclimation

N. lituratus, *A. triostegus* and *C. spilurus* were collected in winter (Jan-Mar, ❄) or summer (Jul-Sep, ☀), and acclimated in the laboratory at mean SSTs of 24.0 or 27.5°C, respectively.

2a.

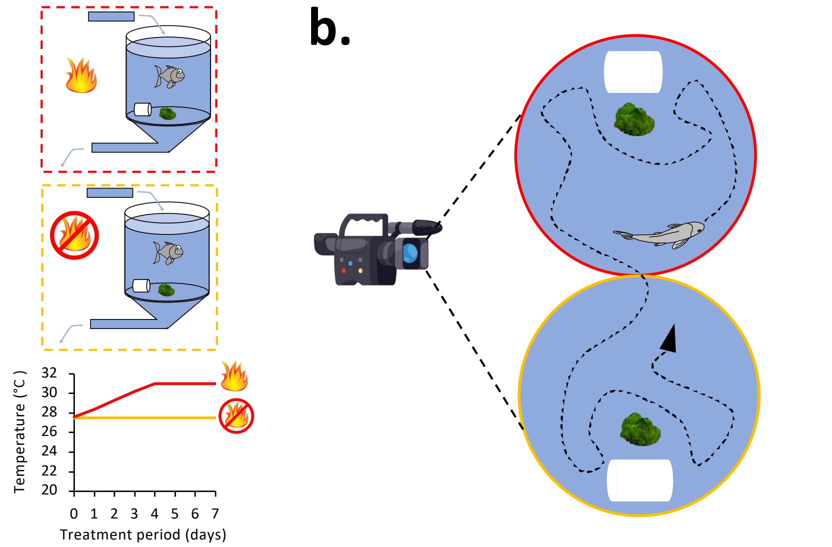

MHW avoidance behaviour

a) Treatment exposure

Fish collected in summer were individually assigned to either…

- ’control’ - no MHW exposure (🚫🔥)
- ’treatment’ - MHW exposure (🔥)

b) Assessment of MHW avoidance

After treatment period, fish were transferred from their treatment tank to the corresponding temperature chamber of the shuttlebox system…

-0-1 h - habituation period

-1-23 h - movements of fish between chambers tracked using LoliTrack software.

3a.

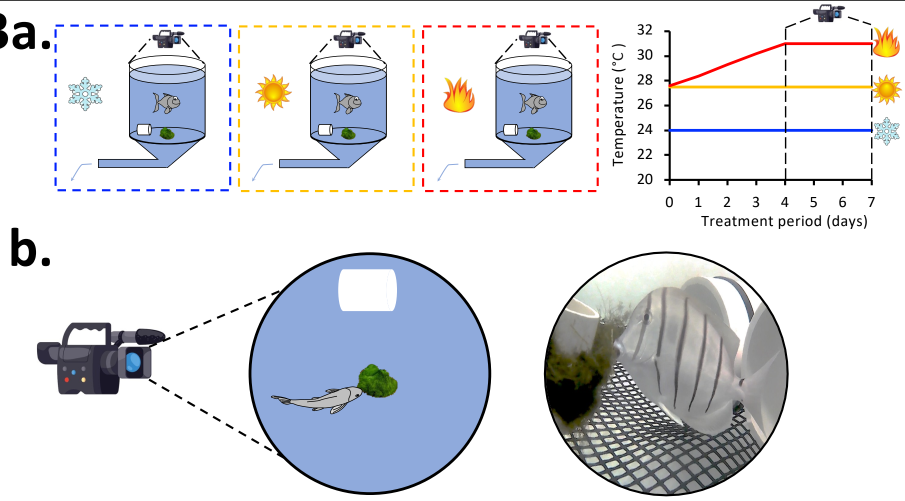

Video-recording times: 09:30-09:40, 11:10-11:20, 12:50-13:00, 14:30-14:40, and 16:10-16:20 HST

Foraging rates & activity

a) Treatment exposure

Fish were fasted for 24 h, then weighed, measured, and assigned to winter (❄), summer (☀), or MHW (🔥) treatment groups.

b) Assessment of foraging rates & activity

Fish were supplied daily with surplus of algal matrix and video-recorded for the last 3 treatment days. Video-recordings were analysed for…

-bites taken from algal matrix per min

-proportion of time spent foraging

4.

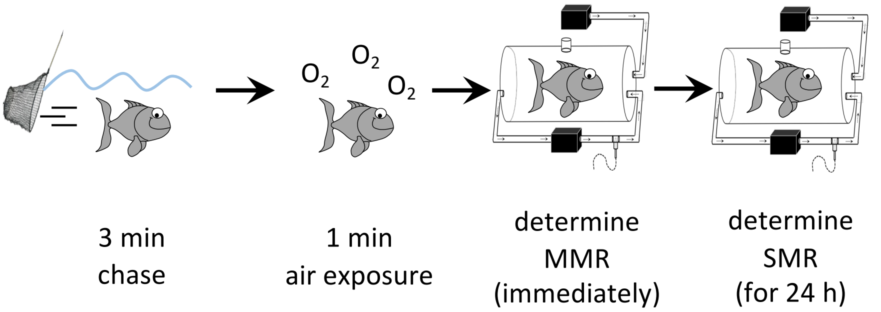

Whole-animal metabolic rates

Following assessment of foraging rates and activity, fish from each treatment (❄, ☀, 🔥) were…

-fasted for 24 h

-weighed and measured

-subjected to a ’chase protocol’ for 3 min

-subjected to air exposure for 1 min

-placed in respirometer to determine MMR

-left in respirometer to determine SMR

5.

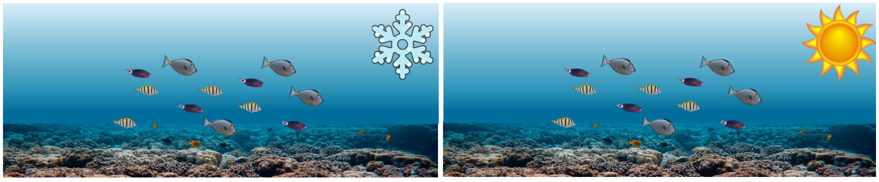

Fish release

At end of experiment, fish were released back into the wild at the approximate location of capture
