# Supplementary material for: Outlasting the Heat: Collapse of Herbivorous Fish Control of Invasive Algae During Marine Heatwaves
Source: Glob Chang Biol. 2025 Aug 20;31(8):e70438. doi: 10.1111/gcb.70438 (PMC12365732; doi:10.1111/gcb.70438)
Supplement: Supplementary file 2 — Data S2: Supporting Information 2. [file GCB-31-e70438-s004.pdf]

Supp. Info. 2: MHW Avoidance Behaviour

2025-02-01 - Jeroen Brijs

SETUP

In this section, we loaded the necessary packages and data, and defined useful plotting utilities.

```
# load packages
library(readr)
library(lme4)
library(lmerTest)
library(ggplot2)
library(emmeans)
library(MuMIn)

# load data into R studio
Heatwave <- read_csv("/Users/xbrije/Documents/Research/20 Herbivores/Stats/Avoidance.csv")

# transformations
Heatwave$sq <- sqrt(Heatwave$Avoidance)
Heatwave$log <- log(Heatwave$Avoidance)
Heatwave$Logit <- log(Heatwave$Avoidance / (1 - Heatwave$Avoidance))
Heatwave$Arcsine <- asin(sqrt(Heatwave$Avoidance))

# turn parameters into factors
Heatwave$Species <- factor(Heatwave$Species)
Heatwave$SpeciesII <- factor(Heatwave$SpeciesII)
Heatwave$Treatment <- factor(Heatwave$Treatment)
Heatwave$Time <- factor(Heatwave$Time)
Heatwave$Individual <- factor(Heatwave$Individual)

# split file into species
split_data <- split(Heatwave, Heatwave$SpeciesII)
NL <- split_data[["NL"]]
AT <- split_data[["AT"]]
CS <- split_data[["CS"]]
```

PROPORTION OF TIME AVOIDING MHW CHAMBER

Data exploration

In this section, we explore the distribution of the untransformed and transformed data.

```
# plot histograms of untransformed and transformed data
par(mfrow = c(2,2))
hist(Heatwave$Avoidance, ylim = c(0, 60), main = "Avoidance", xlab = "Value")
hist(Heatwave$log, ylim = c(0, 60), main = "Log", xlab = "Value")
hist(Heatwave$Logit, ylim = c(0, 60), main = "Logit", xlab = "Value")
```

```
hist(Heatwave$Arcsine, ylim = c(0, 60), main = "Arcsine", xlab = "Value")
```

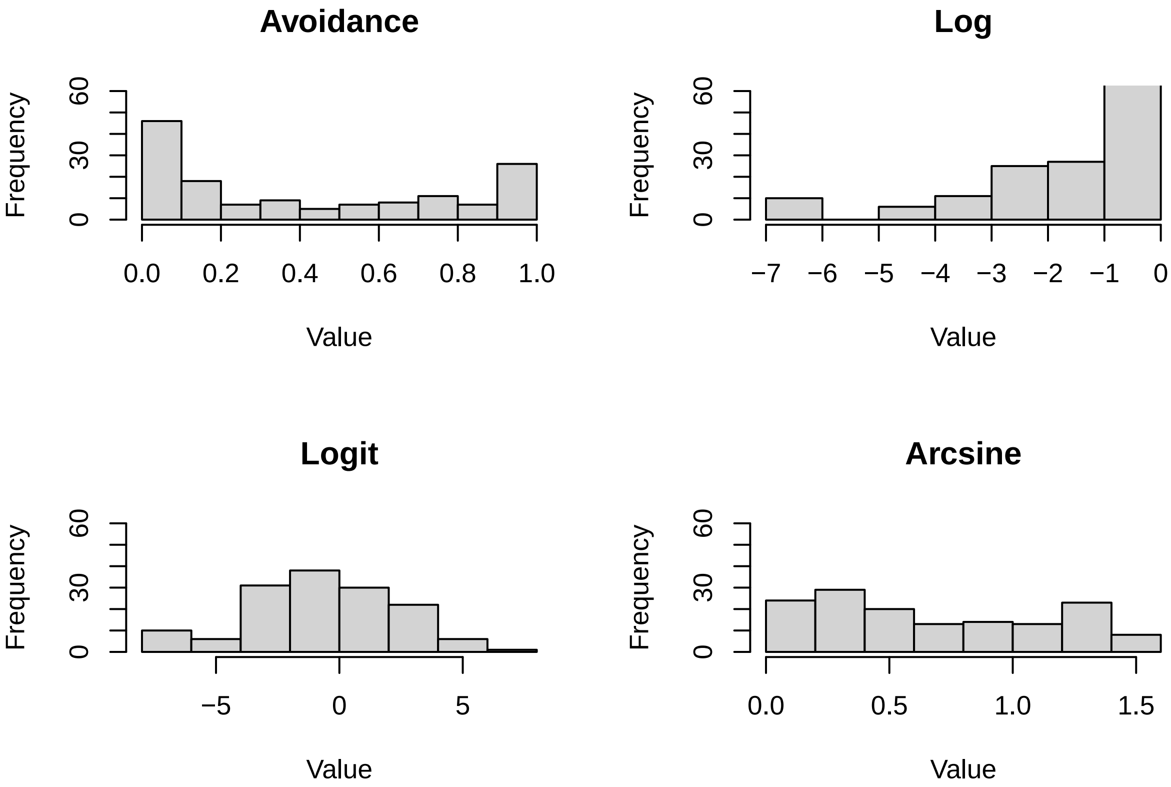

Model fitting and selection

In this section, we built a selection of candidate models for heatwave avoidance behaviour. Avoidance (% of time spent avoiding MHW chamber) was logit transformed to best meet the assumptions underlying linear mixed effect regression models.

```
# fit models
All_model1 = lmer(Logit ~ 1 + (1 | Individual), data = Heatwave)
All_model2 = lmer(Logit ~ Time + (1 | Individual), data = Heatwave)
All_model3 = lmer(Logit ~ Treatment + (1 | Individual), data = Heatwave)
All_model4 = lmer(Logit ~ Species + (1 | Individual), data = Heatwave)
All_model5 = lmer(Logit ~ Time + Treatment + (1 | Individual), data = Heatwave)
All_model6 = lmer(Logit ~ Time + Species + (1 | Individual), data = Heatwave)
All_model7 = lmer(Logit ~ Treatment + Species + (1 | Individual),data = Heatwave)
All_model8 = lmer(Logit ~ Time + Treatment + Time:Treatment + (1 | Individual), data = Heatwave)
All_model9 = lmer(Logit ~ Time + Species + Time:Species + (1 | Individual),data = Heatwave)
All_model10 = lmer(Logit ~ Treatment + Species + Treatment:Species + (1 | Individual), data
              = Heatwave)
All_model11 = lmer(Logit ~ Time + Treatment + Species + (1 | Individual), data = Heatwave)
All_model12 = lmer(Logit ~ Time + Treatment + Species + Time:Treatment + (1 | Individual), data
              = Heatwave)
All_model13 = lmer(Logit ~ Time + Treatment + Species + Time:Species + (1 | Individual), data
              = Heatwave)
All_model14 = lmer(Logit ~ Time + Treatment + Species + Treatment:Species + (1 | Individual),
All_model15 = lmer(Logit ~ Time + Treatment + Species + Time:Treatment + Time:Species + (1 |
              Individual), data = Heatwave)
All_model16 = lmer(Logit ~ Time + Treatment + Species + Time:Treatment + Treatment:Species + (1 |
```

```r
All_model17 = lmer(Logit ~ Time + Treatment + Species + Time:Species + Treatment:Species + (1 |
              Individual), data = Heatwave)
All_model18 = lmer(Logit ~ Time + Treatment + Species + Time:Treatment + Time:Species +
              Treatment:Species + (1 | Individual), data = Heatwave)

# model selection based on AIC
All_aic = AIC(All_model1, All_model2, All_model3, All_model4, All_model5, All_model6, All_model7,
All_aic = All_aic[order(All_aic$AIC), ]
All_aic
```

```
##             df      AIC
## All_model17 11 701.4724
## All_model18 12 702.3649
## All_model13  9 703.1050
## All_model15 10 703.9976
## All_model10  8 704.0500
## All_model3   4 704.4202
## All_model9   8 705.0059
## All_model7   6 705.6826
## All_model14  9 706.3124
## All_model1   3 706.3639
## All_model5   5 706.6826
## All_model16 10 707.1479
## All_model8   6 707.5181
## All_model4   5 707.5835
## All_model11  7 707.9451
## All_model2   4 708.6263
## All_model12  8 708.7806
## All_model6   6 709.8459
```

All of the models for heatwave avoidance behaviour were fit without convergence issues.

Model checking

In this section, we checked the primary assumptions of linear mixed-effects regression models (i.e. normality, homoscedasticity and outliers).

Assumption of normality

```r
# Most parsimonious model
residuals <- resid(All_model17)
hist(residuals, breaks = 20, col = "blue", border = "black", main = "Histogram of Residuals")
```

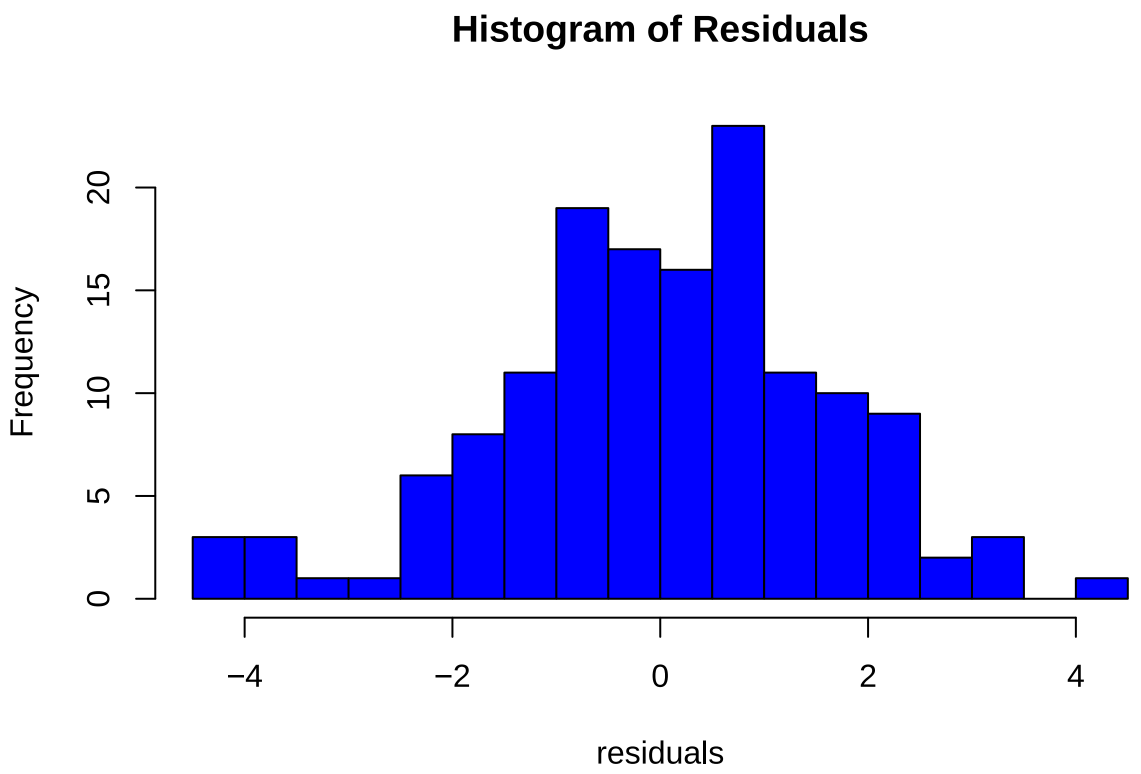


```
shapiro.test(residuals)
```

```
##
##  Shapiro-Wilk normality test
##
## data:  residuals
## W = 0.98651, p-value = 0.1733
```

```
qqnorm(residuals)
qqline(residuals)
```

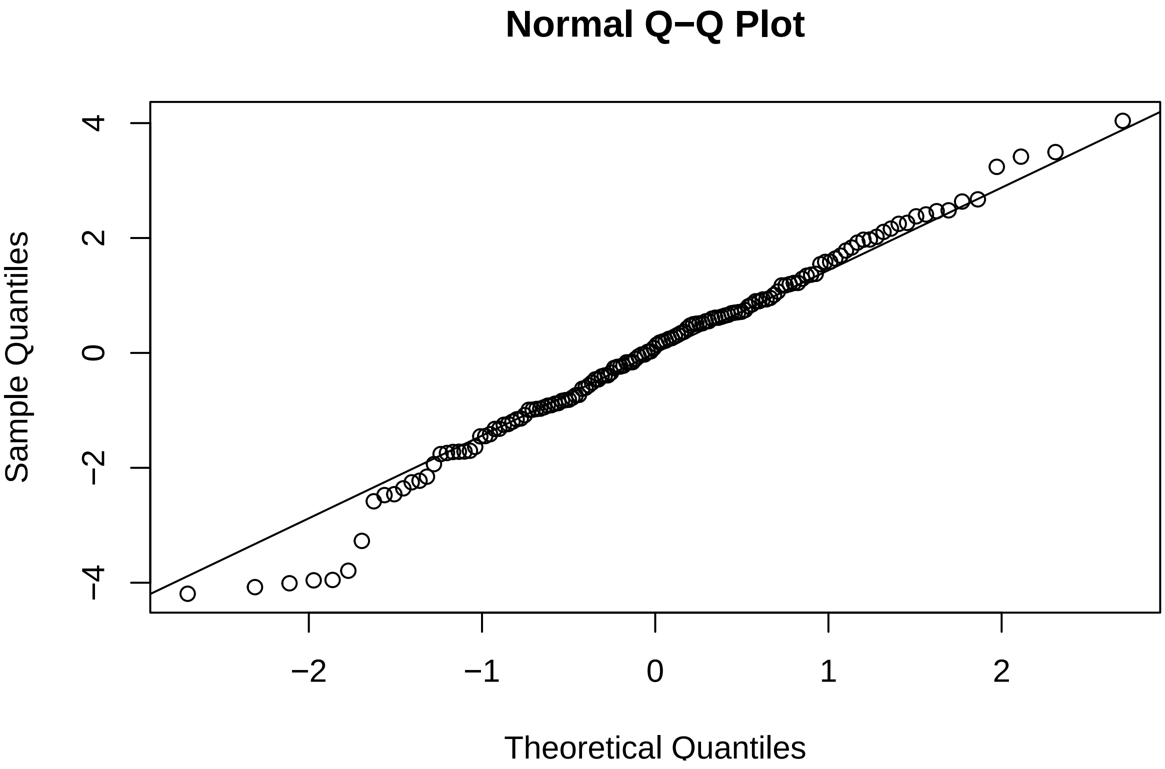

Assumption of homoscedasticity

```
# Most parsimonious model
plot(fitted(All_model17), residuals, ylab = "Residuals", xlab = "Fitted values")
```

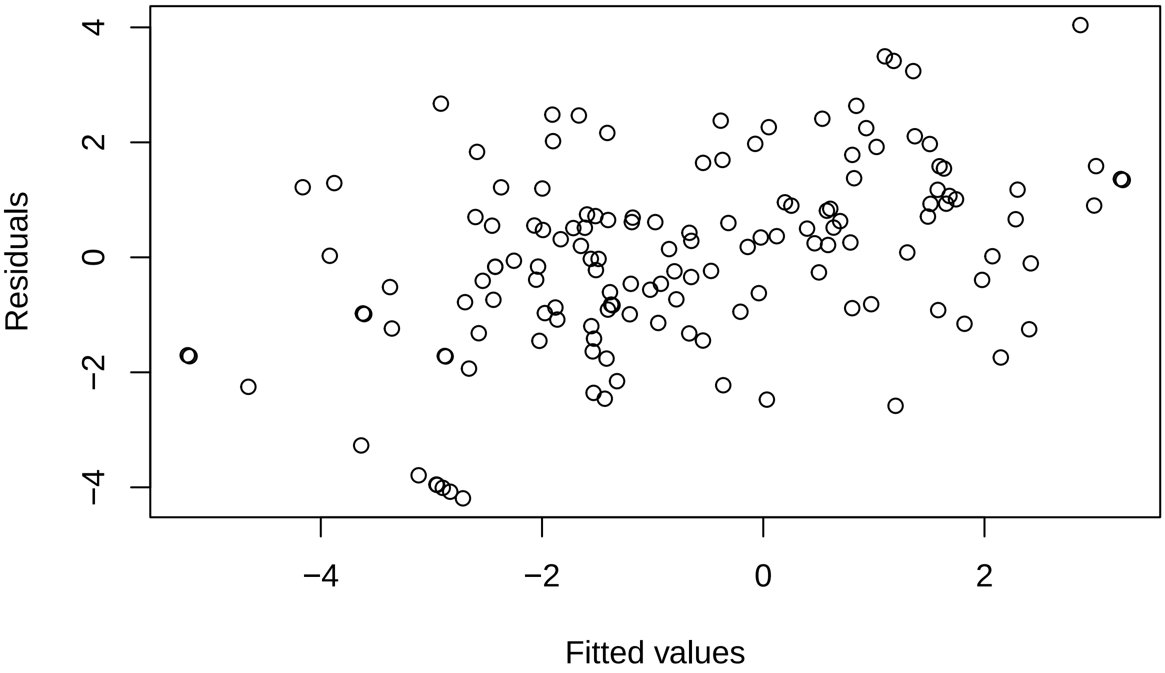

```
plot(Heatwave$Time, residuals(All_model17), ylab = "Residuals", xlab = "Time")
```

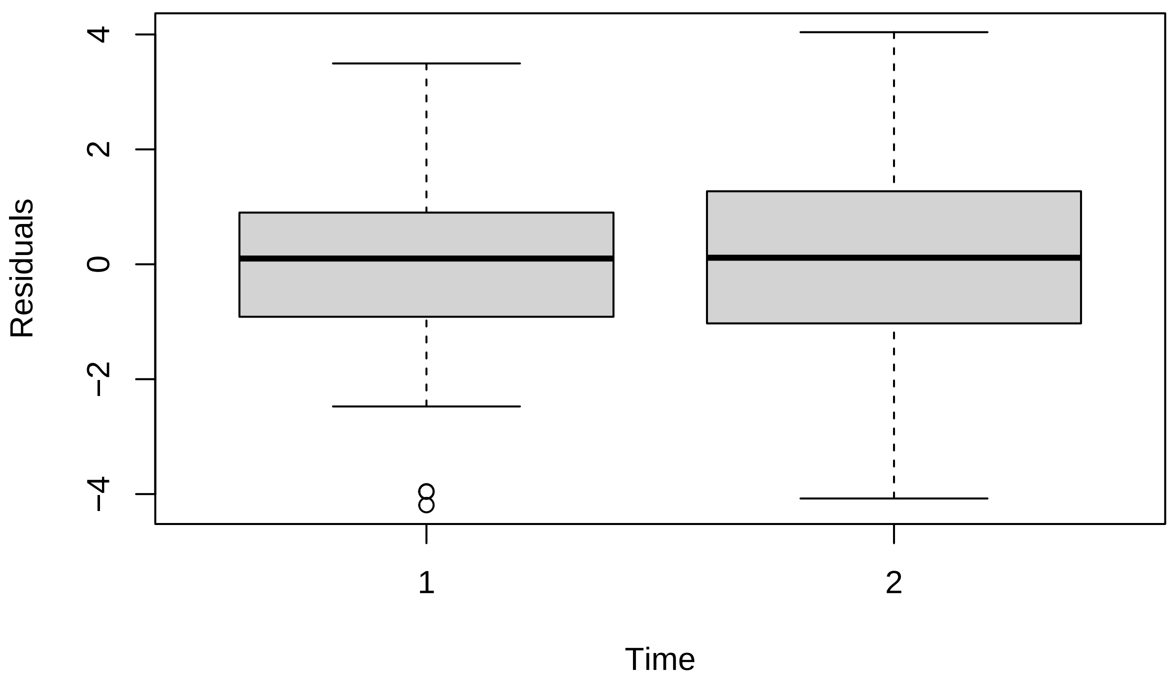

Outliers

```
# Examine standardized residuals versus fitted values
residuals <- resid(All_model17, type = "pearson")
plot(fitted(All_model17), residuals, ylab = "Standardized Residuals", xlab = "Fitted Values")
```

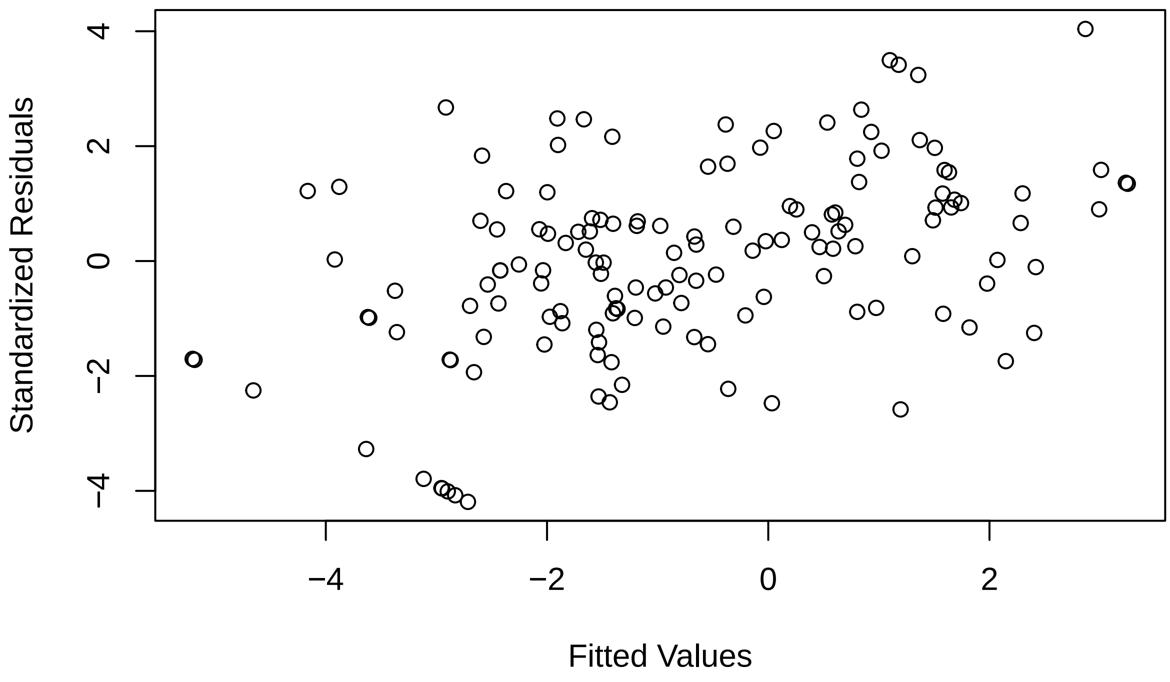


```
# Examine Cooks distances
cooksd <- cooks.distance(All_model17)
plot(cooksd, type = "o", pch = 19, ylab = "Cook's Distance", xlab = "Observation")
```

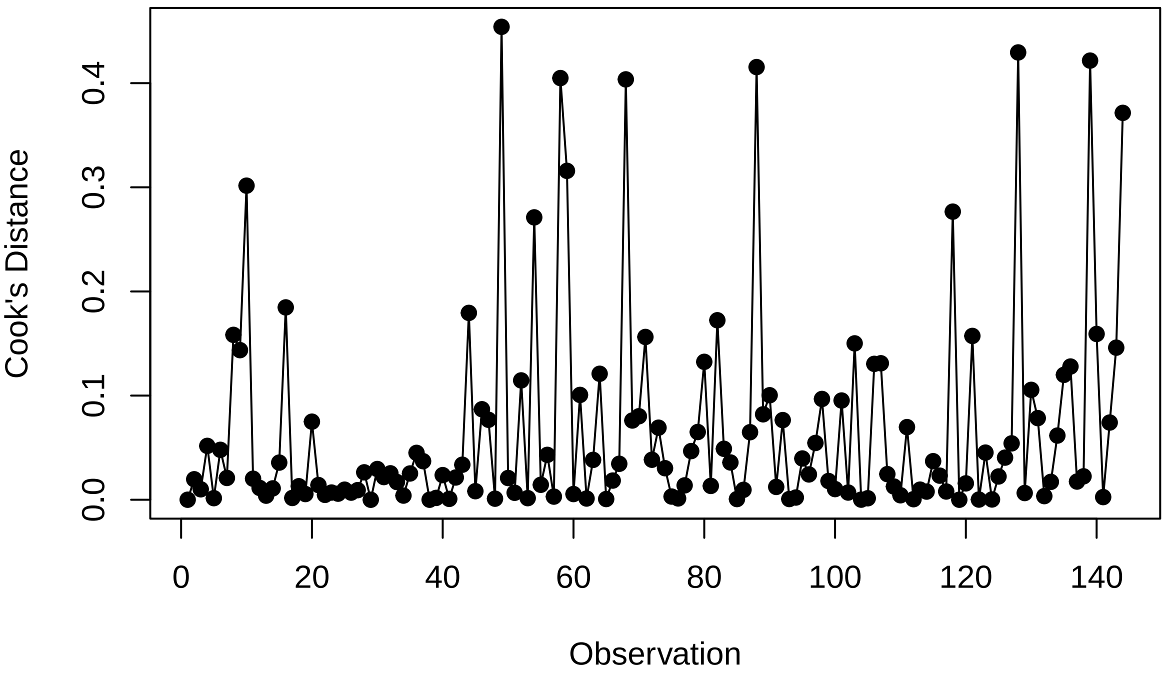

We chose to proceed with All_model17 based on the fact that it had the lowest AIC and best met the assumptions of normality (based on q-q plots and Shapiro-Wilk test statistic), homoscedasticity (based on residuals vs. fitted values) and had no significant outliers (cook's distances were below 1).

Model inference

In this section, we inspected the values of the coefficients and the corresponding confidence intervals for time (Time 1 = 'day', Time 2 = 'night'), treatment (Treatment 1 = Control, Treatment 2 = Treatment), species (Species 1 = N. lituratus, Species 2 = A. triostegus, Species 3 = C. spilurus), and the interactions (Time:Species and Treatment:Species).

```
# generate model summary and confidence interval
summary(All_model17)
```

```
## Linear mixed model fit by REML. t-tests use Satterthwaite's method [
## lmerModLmerTest]
## Formula:
## Logit ~ Time + Treatment + Species + Time:Species + Treatment:Species +
##     (1 | Individual)
##    Data: Heatwave
##
## REML criterion at convergence: 679.5
##
## Scaled residuals:
##      Min       1Q   Median       3Q      Max
## -2.04936 -0.47490  0.05566  0.47416  1.97490
##
## Random effects:
##  Groups     Name        Variance Std.Dev.
##  Individual (Intercept) 4.588    2.142
##  Residual               4.184    2.045
## Number of obs: 144, groups:  Individual, 72
```

```
##
## Fixed effects:
##                      Estimate Std. Error       df t value Pr(>|t|)
## (Intercept)           0.26482    0.80236 86.26465   0.330    0.742
## Time2                 0.01715    0.59046 69.00000   0.029    0.977
## Treatment2           -1.71411    1.05510 66.00000  -1.625    0.109
## Species2             -0.31955    1.13471 86.26465  -0.282    0.779
## Species3             -1.08865    1.13471 86.26465  -0.959    0.340
## Time2:Species2       -1.06465    0.83503 69.00000  -1.275    0.207
## Time2:Species3        1.03087    0.83503 69.00000   1.235    0.221
## Treatment2:Species2   0.75834    1.49214 66.00000   0.508    0.613
## Treatment2:Species3   1.19534    1.49214 66.00000   0.801    0.426
##
## Correlation of Fixed Effects:
##              (Intr) Time2  Trtmn2 Specs2 Specs3 Tm2:S2 Tm2:S3 Tr2:S2
## Time2        -0.368
## Treatment2   -0.657  0.000
## Species2     -0.707  0.260  0.465
## Species3     -0.707  0.260  0.465  0.500
## Time2:Spcs2   0.260 -0.707  0.000 -0.368 -0.184
## Time2:Spcs3   0.260 -0.707  0.000 -0.184 -0.368  0.500
## Trtmnt2:Sp2   0.465  0.000 -0.707 -0.657 -0.329  0.000  0.000
## Trtmnt2:Sp3   0.465  0.000 -0.707 -0.329 -0.657  0.000  0.000  0.500
```

```
r_squared <- r.squaredGLMM(All_model17, muM2 = 0, method = "conditional")
```

```
## Warning: 'r.squaredGLMM' now calculates a revised statistic. See the help page.
```

```
r_squared
```

```
##             R2m       R2c
## [1,] 0.06230385 0.5527433
```

```
anova(All_model17)
```

```
## Type III Analysis of Variance Table with Satterthwaite's method
##                   Sum Sq Mean Sq NumDF DenDF F value   Pr(>F)
## Time              0.0012  0.0012     1    69  0.0003 0.98627
## Treatment        12.7368 12.7368     1    66  3.0444 0.08567 .
## Species           2.3612  1.1806     2    66  0.2822 0.75503
## Time:Species     26.3494 13.1747     2    69  3.1491 0.04912 *
## Treatment:Species  2.7495  1.3748     2    66  0.3286 0.72110
## ---
## Signif. codes:  0 '***' 0.001 '**' 0.01 '*' 0.05 '.' 0.1 ' ' 1
```

```
# calculate estimated marginal means for each combination of time, treatment and species
emms <- emmeans(All_model17, ~ Time * Treatment * Species)
print(emms)
```

```
##  Time Treatment Species  emmean    SE   df lower.CL upper.CL
##  1    1         1        0.2648 0.802 86.3    -1.33    1.860
##  2    1         1        0.2820 0.802 86.3    -1.31    1.877
##  1    2         1       -1.4493 0.802 86.3    -3.04    0.146
##  2    2         1       -1.4321 0.802 86.3    -3.03    0.163
##  1    1         2       -0.0547 0.802 86.3    -1.65    1.540
##  2    1         2       -1.1022 0.802 86.3    -2.70    0.493
##  1    2         2       -1.0105 0.802 86.3    -2.61    0.584
```

```
## 2 2 2 -2.0580 0.802 86.3 -3.65 -0.463
## 1 1 3 -0.8238 0.802 86.3 -2.42 0.771
## 2 1 3 0.2242 0.802 86.3 -1.37 1.819
## 1 2 3 -1.3426 0.802 86.3 -2.94 0.252
## 2 2 3 -0.2946 0.802 86.3 -1.89 1.300
##
## Degrees-of-freedom method: kenward-roger
## Confidence level used: 0.95
```

```
# perform interaction contrasts - effect of time within each species
NL_emmeans <- emmeans(All_model17, ~ Time, at = list(Species = "1"))
NL_contrasts <- pairs(NL_emmeans, adjust = "none")
NL_summary <- summary(NL_contrasts)
NL_p_values <- NL_summary$p.value
NL_emmeans
```

```
##  Time emmean    SE  df lower.CL upper.CL
##  1    -0.592 0.605 104    -1.79    0.607
##  2    -0.575 0.605 104    -1.77    0.624
##
## Results are averaged over the levels of: Treatment
## Degrees-of-freedom method: kenward-roger
## Confidence level used: 0.95
```

```
AT_emmeans <- emmeans(All_model17, ~ Time, at = list(Species = "2"))
AT_contrasts <- pairs(AT_emmeans, adjust = "none")
AT_summary <- summary(AT_contrasts)
AT_p_values <- AT_summary$p.value
AT_emmeans
```

```
##  Time emmean    SE  df lower.CL upper.CL
##  1    -0.533 0.605 104    -1.73    0.666
##  2    -1.580 0.605 104    -2.78   -0.381
##
## Results are averaged over the levels of: Treatment
## Degrees-of-freedom method: kenward-roger
## Confidence level used: 0.95
```

```
CS_emmeans <- emmeans(All_model17, ~ Time, at = list(Species = "3"))
CS_contrasts <- pairs(CS_emmeans, adjust = "none")
CS_summary <- summary(CS_contrasts)
CS_p_values <- CS_summary$p.value
CS_emmeans
```

```
##  Time  emmean    SE  df lower.CL upper.CL
##  1    -1.0832 0.605 104    -2.28    0.116
##  2    -0.0352 0.605 104    -1.23    1.164
##
## Results are averaged over the levels of: Treatment
## Degrees-of-freedom method: kenward-roger
## Confidence level used: 0.95
```

```
# perform interaction contrasts - effect of treatment within each species
NLTreatment_emmeans <- emmeans(All_model17, ~ Treatment, at = list(Species = "1"))
NLTreatment_contrasts <- pairs(NLTreatment_emmeans, adjust = "none")
NLTreatment_summary <- summary(NLTreatment_contrasts)
NLTreatment_p_values <- NLTreatment_summary$p.value
```

```r
NLTreatment_emmeans
```

```
##  Treatment emmean    SE df lower.CL upper.CL
##  1          0.273 0.746 66    -1.22   1.7630
##  2         -1.441 0.746 66    -2.93   0.0489
##
## Results are averaged over the levels of: Time
## Degrees-of-freedom method: kenward-roger
## Confidence level used: 0.95
```

```r
ATTreatment_emmeans <- emmeans(All_model17, ~ Treatment, at = list(Species = "2"))
ATTreatment_contrasts <- pairs(ATTreatment_emmeans, adjust = "none")
ATTreatment_summary <- summary(ATTreatment_contrasts)
ATTreatment_p_values <- ATTreatment_summary$p.value
ATTreatment_emmeans
```

```
##  Treatment emmean    SE df lower.CL upper.CL
##  1         -0.578 0.746 66    -2.07   0.9111
##  2         -1.534 0.746 66    -3.02  -0.0447
##
## Results are averaged over the levels of: Time
## Degrees-of-freedom method: kenward-roger
## Confidence level used: 0.95
```

```r
CSTreatment_emmeans <- emmeans(All_model17, ~ Treatment, at = list(Species = "3"))
CSTreatment_contrasts <- pairs(CSTreatment_emmeans, adjust = "none")
CSTreatment_summary <- summary(CSTreatment_contrasts)
CSTreatment_p_values <- CSTreatment_summary$p.value
CSTreatment_emmeans
```

```
##  Treatment emmean    SE df lower.CL upper.CL
##  1         -0.300 0.746 66    -1.79    1.190
##  2         -0.819 0.746 66    -2.31    0.671
##
## Results are averaged over the levels of: Time
## Degrees-of-freedom method: kenward-roger
## Confidence level used: 0.95
```

```r
# perform FDR correction on all contrasts
All_p_values <- c(NL_p_values, AT_p_values, CS_p_values, NLTreatment_p_values,
                  ATTreatment_p_values, CSTreatment_p_values)
All_fdr_corrected_p <- p.adjust(All_p_values, method = "fdr")

All_contrast_names <- c("NL day - NL night","AT day - AT night","CS day - CS night",
                        "NL control - NL treatment", "AT control - AT treatment",
                        "CS control - CS treatment")
All_fdr_corrected_p_named <- setNames(All_fdr_corrected_p, All_contrast_names)
All_fdr_corrected_p_named
```

```
##         NL day - NL night         AT day - AT night         CS day - CS night
##                 0.9769164                 0.2180336                 0.2180336
## NL control - NL treatment AT control - AT treatment CS control - CS treatment
##                 0.2180336                 0.5524606                 0.7494955
```

The fixed and random effects of the linear mixed-effects model for MHW avoidance explained 6% and 49% of the variation in the proportion of time that fish spent avoiding the MHW chamber, respectively. Model output revealed that the interaction between time and species (F2,69=3.1491, p=0.0491) had a significant effect

on MHW avoidance behaviour, whereas treatment ($F2,69=3.0444$, $p=0.0857$) and the interaction between treatment and species did not ($F2,66=0.3286$, $p=0.7211$).

In addition, planned contrasts revealed that the proportion of time that fish spent avoiding the MHW chamber did not differ for any of the species with regards to time ('day' vs. 'night': $p = 0.9769$, 0.2180 and 0.2180 for N. lituratus, A. triostegus, and C. spilurus, respectively) or treatment ('control' vs. 'treatment': $p = 0.2180$, 0.5525 and 0.7495 for N. lituratus, A. triostegus, and C. spilurus, respectively).

PROBABILITY OF AVOIDING MHW CHAMBER

Based on the output of the above model, exact binomial tests with exact Clopper-Pearson 95% C.I. were subsequently performed using the total proportion of time an individual spent avoiding the MHW chamber during the 22-hour experimental period as the 'success' category and all individuals within each species were included regardless of treatment.

```
# Example observed proportions
NLobserved_proportions <- NL$AvoidanceII
ATobserved_proportions <- AT$AvoidanceII
CSobserved_proportions <- CS$AvoidanceII

# Define the expected proportion
expected_proportion <- 0.5

# Calculate the total number of trials
NLnum_trials <- length(NLobserved_proportions)
ATnum_trials <- length(ATobserved_proportions)
CSnum_trials <- length(CSobserved_proportions)

# Calculate the number of successes
NLnum_avoid <- sum(NLobserved_proportions > 0.5)
ATnum_avoid <- sum(ATobserved_proportions > 0.5)
CSnum_avoid <- sum(CSobserved_proportions > 0.5)

# Perform the binomial test
NLbinomial_test <- binom.test(NLnum_avoid, NLnum_trials, expected_proportion)
ATbinomial_test <- binom.test(ATnum_avoid, ATnum_trials, expected_proportion)
CSbinomial_test <- binom.test(CSnum_avoid, CSnum_trials, expected_proportion)

# Print the result
print(NLbinomial_test)
```

```
##
##  Exact binomial test
##
## data:  NLnum_avoid and NLnum_trials
## number of successes = 8, number of trials = 24, p-value = 0.1516
## alternative hypothesis: true probability of success is not equal to 0.5
## 95 percent confidence interval:
##  0.1563023 0.5532196
## sample estimates:
## probability of success
##              0.3333333
```

```
print(ATbinomial_test)
```

```
##
##  Exact binomial test
##
## data:  ATnum_avoid and ATnum_trials
## number of successes = 9, number of trials = 24, p-value = 0.3075
## alternative hypothesis: true probability of success is not equal to 0.5
## 95 percent confidence interval:
##  0.1879929 0.5940636
## sample estimates:
## probability of success
##                  0.375
```

```
print(CSbinomial_test)
```

```
##
##  Exact binomial test
##
## data:  CSnum_avoid and CSnum_trials
## number of successes = 9, number of trials = 24, p-value = 0.3075
## alternative hypothesis: true probability of success is not equal to 0.5
## 95 percent confidence interval:
##  0.1879929 0.5940636
## sample estimates:
## probability of success
##                  0.375
```

Exact binomial tests demonstrated that the probability of an individual successfully avoiding the MHW chamber did not significantly differ from random (i.e., 50%) for N. lituratus (95% CI: 16-55%, p=0.1515), A. triostegus (95% CI: 19-59%, p=0.3075) or C. spilurus (95% CI: 19-59%, p=0.3075).
