# Supplementary material for: Outlasting the Heat: Collapse of Herbivorous Fish Control of Invasive Algae During Marine Heatwaves
Source: Glob Chang Biol. 2025 Aug 20;31(8):e70438. doi: 10.1111/gcb.70438 (PMC12365732; doi:10.1111/gcb.70438)
Supplement: Supplementary file 3 — Data S3: Supporting Information 3. [file GCB-31-e70438-s003.pdf]

Supp. Info. 3: Metabolic statistical analyses

2025-02-01 - Jeroen Brijs

SETUP

In this section, we loaded the necessary packages and data, and defined useful plotting utilities.

```
# load packages
library(readr) # for loading data file
library(dplyr) # for data manipulation
library(ggplot2) # for plotting
library(ggstance) # for plotting
library(cowplot) # for plotting
library(lmtest) # for assumption testing
library(emmeans) # for posthoc analyses
library(openxlsx) # for exporting to excel

# load data into R studio
Metabolism <- read_csv("/Users/xbrije/Documents/Research/20 Herbivores/Stats/Metabolism.csv")

# change from a numeric to a factor independent variable and clarify treatment colours
Metabolism$Species <- factor(Metabolism$Species)
Metabolism$Treatment <- factor(Metabolism$Treatment)
Treatment_colors <- c("1" = "blue", "2" = "orange", "3" = "red")
Treatment_labels <- c("1" = "Winter", "2" = "Summer", "3" = "MHW")
Species_labels <- c("1" = "NL", "2" = "AT", "3" = "CS")
```

STANDARD METABOLIC RATE (SMR)

Data exploration (SMR)

In this section, we explore the relationship between SMR and body mass for all species of herbivores.

```
# plot untransformed dependent variable (SMR) vs. continuous independent variable (Mass)
smr_NL_linearplot <- ggplot(Metabolism %>% filter(Species == "1"), aes(x = Mass, y = SMR,
  color = Treatment)) +
  geom_point() +
  geom_smooth(method = "lm", se = FALSE, formula = y ~ x) +
  ggtitle("NL") +
  scale_color_manual(values = Treatment_colors, labels = Treatment_labels) +
  labs(x = "Mass (g)", y = "SMR (mg O2/h/kg)") +
  theme(legend.position = "none") +
  coord_cartesian(xlim = c(0, 150), ylim = c(50, 350))

smr_AT_linearplot <- ggplot(Metabolism %>% filter(Species == "2"), aes(x = Mass, y = SMR,
  color = Treatment)) +
  geom_point() +
  geom_smooth(method = "lm", se = FALSE, formula = y ~ x) +
```

```
  ggtitle("AT") +
  scale_color_manual(values = Treatment_colors, labels = Treatment_labels) +
  labs(x = "Mass (g)", y = "") +
  theme(legend.position = "none") +
  coord_cartesian(xlim = c(0, 150), ylim = c(50, 350))

smr_CS_linearplot <- ggplot(Metabolism %>% filter(Species == "3"), aes(x = Mass, y = SMR,
  color = Treatment)) +
  geom_point() +
  geom_smooth(method = "lm", se = FALSE, formula = y ~ x) +
  ggtitle("CS") +
  scale_color_manual(values = Treatment_colors, labels = Treatment_labels) +
  labs(x = "Mass (g)", y = "") +
  theme(legend.position = "none") +
  coord_cartesian(xlim = c(0, 150), ylim = c(50, 350))

legend <- get_legend(
  ggplot(Metabolism %>% filter(Species == "1"), aes(x = Mass, y = SMR, color = Treatment)) +
    geom_point() +
    scale_color_manual(values = Treatment_colors, labels = Treatment_labels) +
    theme(legend.box.margin = margin(0, 0, 0, 12)))
```

```
## Warning in get_plot_component(plot, "guide-box"): Multiple components found;
## returning the first one. To return all, use `return_all = TRUE`.
```

```
SMR_plot <- plot_grid(
  plot_grid(smr_NL_linearplot, smr_AT_linearplot, smr_CS_linearplot, nrow = 1, rel_widths
  = c(1, 1, 1)), legend, ncol = 2, rel_widths = c(3, 0.5))

print(SMR_plot)
```

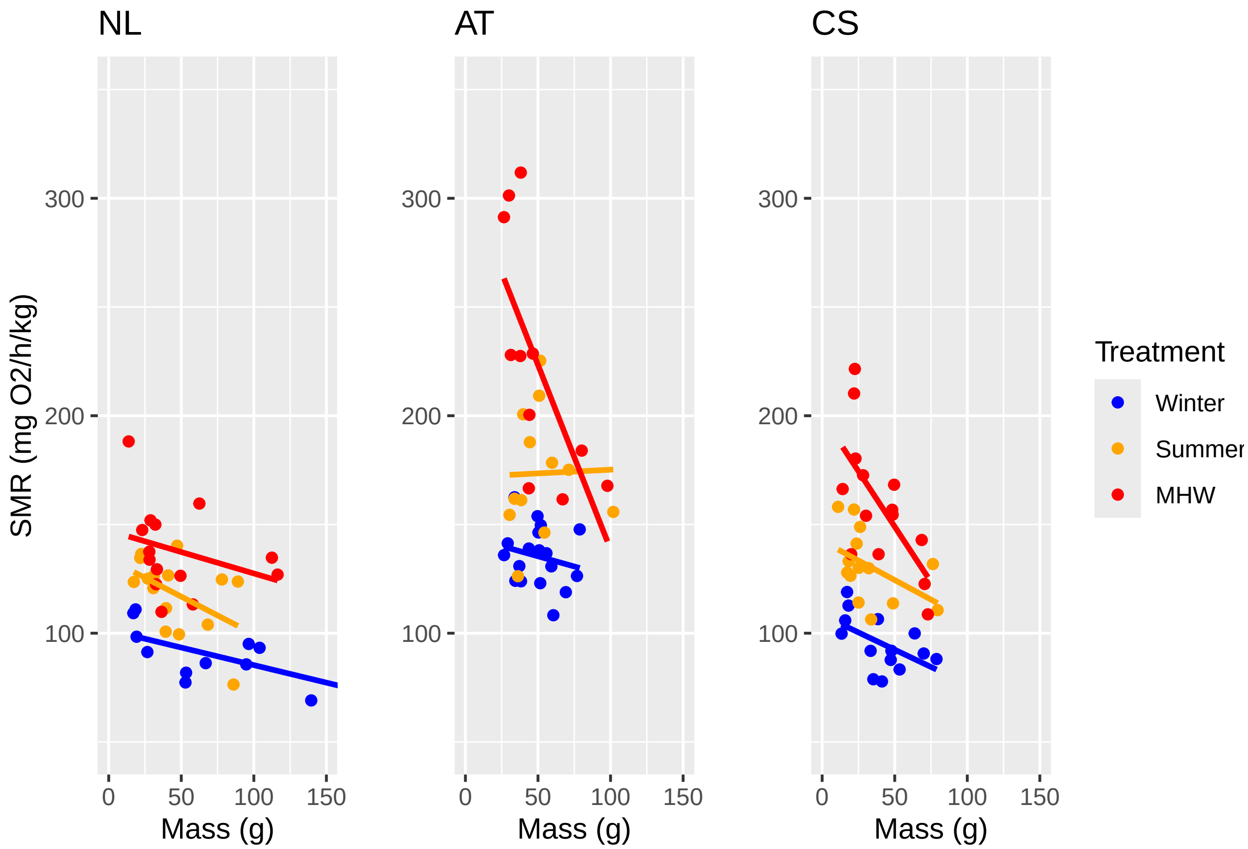


```
# plot transformed dependent variable (lnSMR) vs. continuous independent variable (lnMass)
lnsmr_NL_linearplot <- ggplot(Metabolism %>% filter(Species == "1"), aes(x = lnMass,
  y = lnSMR, color = Treatment)) +
  geom_point() +
  geom_smooth(method = "lm", se = FALSE, formula = y ~ x) +
  ggtitle("NL") +
  scale_color_manual(values = Treatment_colors, labels = Treatment_labels) +
  labs(x = "lnMass (g)", y = "lnSMR (mg O2/h/kg)") +
  theme(legend.position = "none") +
  coord_cartesian(xlim = c(2.5, 5.0), ylim = c(4.00, 5.75))

lnsmr_AT_linearplot <- ggplot(Metabolism %>% filter(Species == "2"), aes(x = lnMass,
  y = lnSMR, color = Treatment)) +
  geom_point() +
  geom_smooth(method = "lm", se = FALSE, formula = y ~ x) +
  ggtitle("AT") +
  scale_color_manual(values = Treatment_colors, labels = Treatment_labels) +
  labs(x = "lnMass (g)", y = "") +
  theme(legend.position = "none") +
  coord_cartesian(xlim = c(2.5, 5.0), ylim = c(4.00, 5.75))

lnsmr_CS_linearplot <- ggplot(Metabolism %>% filter(Species == "3"), aes(x = lnMass,
  y = lnSMR, color = Treatment)) +
  geom_point() +
  geom_smooth(method = "lm", se = FALSE, formula = y ~ x) +
  ggtitle("CS") +
  scale_color_manual(values = Treatment_colors, labels = Treatment_labels) +
```

```
  labs(x = "lnMass (g)", y = "") +
  theme(legend.position = "none") +
  coord_cartesian(xlim = c(2.5, 5.0), ylim = c(4.00, 5.75))

legend <- get_legend(
  ggplot(Metabolism %>% filter(Species == "1"), aes(x = lnMass, y = lnSMR, color =
    Treatment)) +
    geom_point() +
    scale_color_manual(values = Treatment_colors, labels = Treatment_labels) +
    theme(legend.box.margin = margin(0, 0, 0, 12)))
```

```
## Warning in get_plot_component(plot, "guide-box"): Multiple components found;
## returning the first one. To return all, use `return_all = TRUE`.
```

```
lnSMR_plot <- plot_grid(
  plot_grid(lnsmr_NL_linearplot, lnsmr_AT_linearplot, lnsmr_CS_linearplot, nrow = 1,
  rel_widths = c(1, 1, 1)), legend, ncol = 2, rel_widths = c(3, 0.5))

print(lnSMR_plot)
```

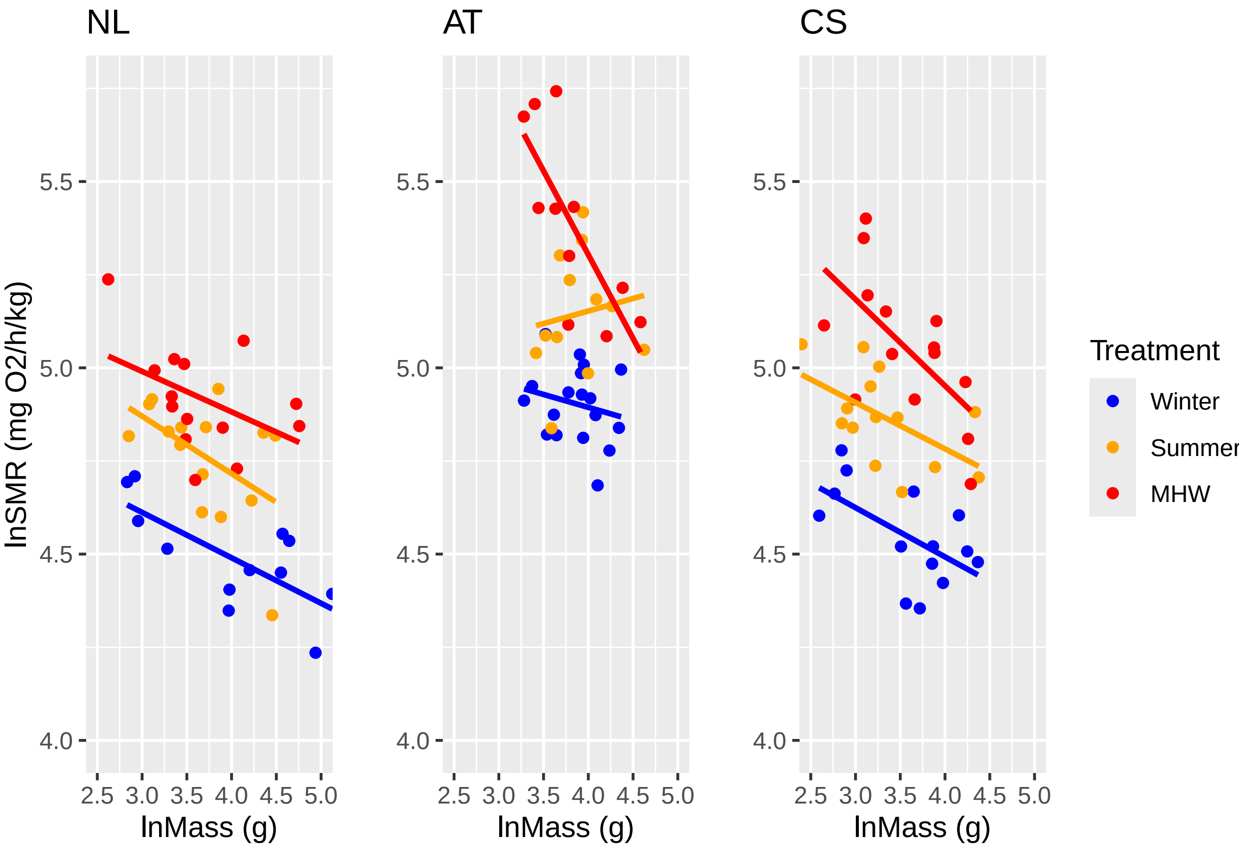

Model fitting (SMR)

In this section, we built a selection of candidate models for SMR. SMR and body mass were log transformed to best meet the assumptions underlying linear regression models.

```
# fit models
smr_model1 = lm(lnSMR ~ 1, data = Metabolism)
smr_model2 = lm(lnSMR ~ lnMass, data = Metabolism)
```

```r
smr_model3 = lm(lnSMR ~ Treatment, data = Metabolism)
smr_model4 = lm(lnSMR ~ Species, data = Metabolism)
smr_model5 = lm(lnSMR ~ lnMass + Treatment, data = Metabolism)
smr_model6 = lm(lnSMR ~ lnMass + Species, data = Metabolism)
smr_model7 = lm(lnSMR ~ Treatment + Species, data = Metabolism)
smr_model8 = lm(lnSMR ~ lnMass * Treatment, data = Metabolism)
smr_model9 = lm(lnSMR ~ lnMass * Species, data = Metabolism)
smr_model10 = lm(lnSMR ~ Treatment * Species, data = Metabolism)
smr_model11 = lm(lnSMR ~ lnMass + Treatment + Species, data = Metabolism)
smr_model12 = lm(lnSMR ~ lnMass * Treatment + Species, data = Metabolism)
smr_model13 = lm(lnSMR ~ Treatment + lnMass * Species, data = Metabolism)
smr_model14 = lm(lnSMR ~ lnMass + Treatment * Species, data = Metabolism)
smr_model15 = lm(lnSMR ~ lnMass * Treatment + lnMass * Species, data = Metabolism)
smr_model16 = lm(lnSMR ~ lnMass * Treatment + Treatment * Species, data = Metabolism)
smr_model17 = lm(lnSMR ~ lnMass * Species + Treatment * Species, data = Metabolism)
smr_model18 = lm(lnSMR ~ lnMass * Treatment + lnMass * Species + Treatment * Species,
              data = Metabolism)
```

All of the models for SMR were fit without convergence issues.

Model selection (SMR)

In this section, we selected the best-fitting model based on Akaike's Information Criterion (AIC) from the set of candidate models (Burnham and Anderson, 2004).

```r
# model selection based on AIC
smr_aic = AIC(smr_model1, smr_model2, smr_model3, smr_model4, smr_model5, smr_model6,
    smr_model7, smr_model8, smr_model9, smr_model10, smr_model11, smr_model12, smr_model13,
    smr_model14, smr_model15, smr_model16, smr_model17, smr_model18)
smr_aic = smr_aic[order(smr_aic$AIC), ]
smr_aic
```

```
##             df         AIC
## smr_model12  9 -135.839033
## smr_model11  7 -135.720825
## smr_model15 11 -132.754173
## smr_model13  9 -132.753498
## smr_model16 13 -131.267845
## smr_model14 11 -131.245222
## smr_model17 13 -127.925788
## smr_model18 15 -127.881175
## smr_model7   6 -104.742785
## smr_model10 10  -98.667289
## smr_model6   5   -6.141174
## smr_model5   5   -4.041125
## smr_model9   7   -2.214031
## smr_model8   7   -1.777788
## smr_model3   4   -0.656854
## smr_model4   4   10.817915
## smr_model2   3   44.579078
## smr_model1   2   49.289080
```

Model checking (SMR)

In this section, we checked the primary assumptions of linear regression models (i.e. normality, homoscedasticity and outliers).

Assumption of normality

```
# check assumption of normality for top four models
# q-q plot
plot(smr_model12, which = 2)
```

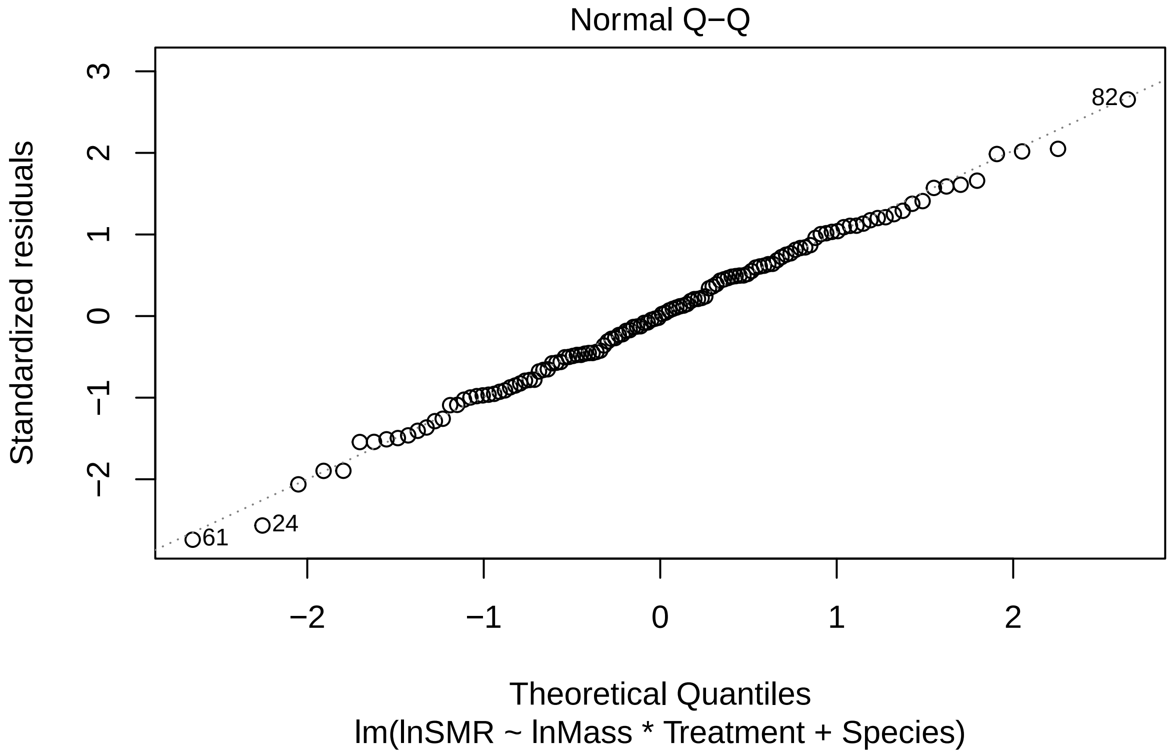


```
# shapiro-wilk test
shapiro.test(resid(smr_model12))
```

```
##
##  Shapiro-Wilk normality test
##
## data:  resid(smr_model12)
## W = 0.99671, p-value = 0.9941
```

Assumption of homoscedasticity

```
# check assumption of homoscedasticity for top four models
# plot residuals vs. fitted, plot standardized residuals vs. fitted
par(mfrow = c(2, 2))
plot(smr_model12, which = 1, main = "Residuals vs. fitted")
plot(smr_model12, which = 3, main = "Std. Residuals vs. fitted")
```

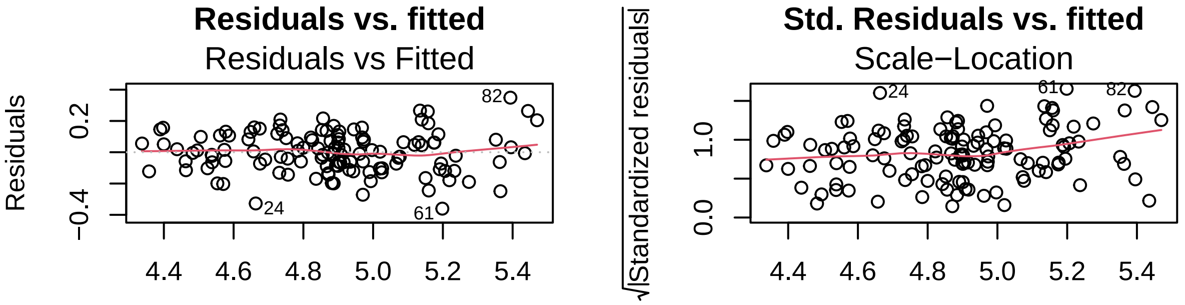

Outliers

```r
# check for outliers in top four models
# plot studentized residuals, cook's distance, and leverage
smra_stud.resid <- rstudent(smr_model12)
par(mfrow = c(1, 3))
plot(smra_stud.resid, main = "Studentized residuals")
plot(smr_model12, which = 4, main = "Cook's distance")
plot(smr_model12, which = 5, main = "Leverage")
```

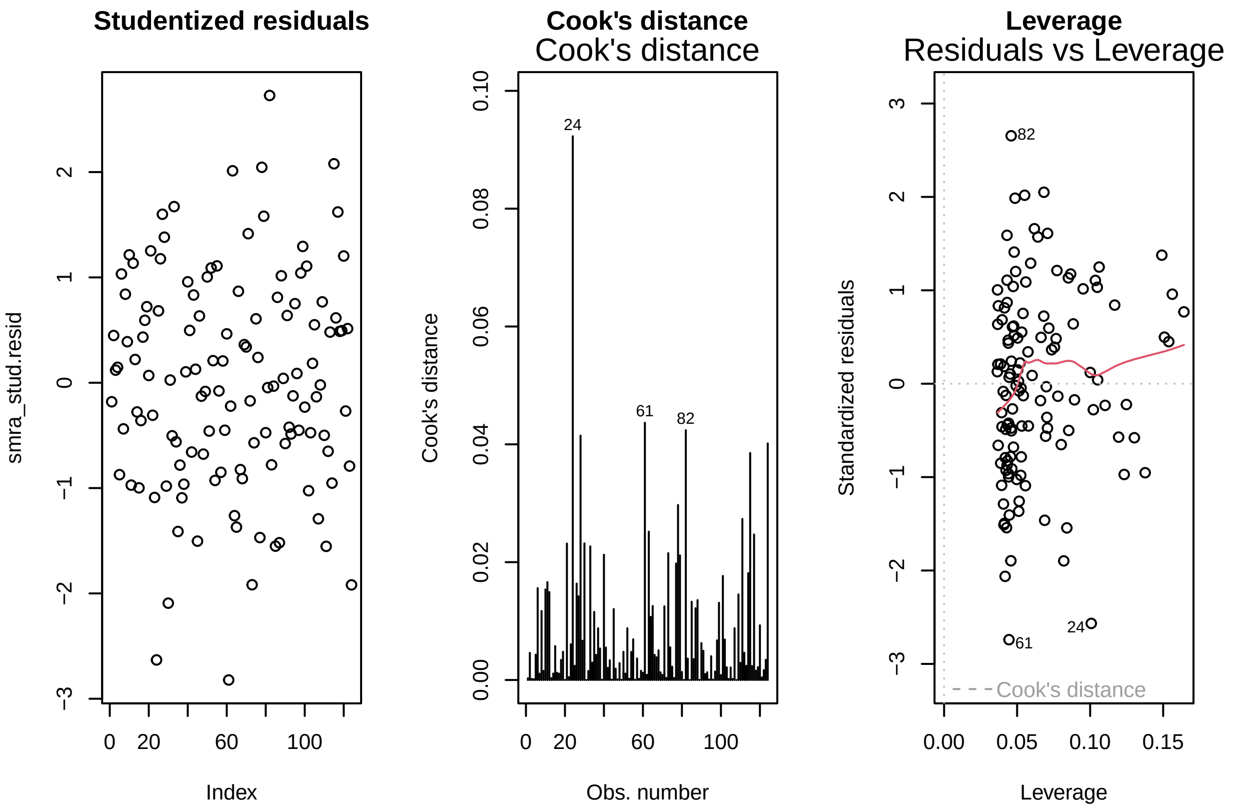

We chose to proceed with smr_model12 based on the fact that it had the lowest AIC and best met the assumptions of normality (based on q-q plots and Shapiro-Wilk test statistic), homoscedasticity (based on residuals vs. fitted values, standardized residuals vs. fitted values) and had no significant outliers (studentized residuals between -3 and 3, cook's distances were below 1, and/or leverage was less than 0.5).

Model inference (SMR)

In this section, we inspected the values of the coefficients and the corresponding confidence intervals for body mass, treatment (1 = winter, 2 = summer, 3 = MHW), species (1= NL, 2 = AT, 3 = CS), and the body mass:treatment interaction.

```
# generate model summary and confidence interval
summary(smr_model12)
```

```
##
## Call:
## lm(formula = lnSMR ~ lnMass * Treatment + Species, data = Metabolism)
##
## Residuals:
##      Min       1Q   Median       3Q      Max
## -0.36045 -0.08762  0.00029  0.09110  0.34881
##
## Coefficients:
##                    Estimate Std. Error t value Pr(>|t|)
## (Intercept)        4.895386   0.140352  34.879  < 2e-16 ***
## lnMass            -0.108875   0.035614  -3.057 0.002775 **
## Treatment2         0.292780   0.202006   1.449 0.149933
## Treatment3         0.833376   0.206920   4.028 0.000101 ***
## Species2           0.433007   0.030076  14.397  < 2e-16 ***
## Species3           0.062838   0.030471   2.062 0.041420 *
## lnMass:Treatment2 -0.008934   0.053893  -0.166 0.868621
## lnMass:Treatment3 -0.102083   0.054866  -1.861 0.065334 .
## ---
## Signif. codes:  0 '***' 0.001 '**' 0.01 '*' 0.05 '.' 0.1 ' ' 1
##
## Residual standard error: 0.1345 on 116 degrees of freedom
## Multiple R-squared:  0.7993, Adjusted R-squared:  0.7872
## F-statistic: 65.99 on 7 and 116 DF,  p-value: < 2.2e-16
```

```
confint(smr_model12)
```

```
##                          2.5 %       97.5 %
## (Intercept)        4.617401025  5.173370334
## lnMass            -0.179412039 -0.038337161
## Treatment2        -0.107317132  0.692878053
## Treatment3         0.423545061  1.243206096
## Species2           0.373438263  0.492575240
## Species3           0.002486339  0.123189110
## lnMass:Treatment2 -0.115676956  0.097808401
## lnMass:Treatment3 -0.210752462  0.006585552
```

```
# create a forest plot for model coefficients and confidence intervals
smr_forestplot_coef <- data.frame(
  Variable = rownames(summary(smr_model12)$coefficients),
  Estimate = summary(smr_model12)$coefficients[, 1],
  Lower = confint(smr_model12)[, 1],
  Upper = confint(smr_model12)[, 2])

ggplot(smr_forestplot_coef, aes(x = Estimate, y = Variable)) +
  geom_errorbarh(aes(xmin = Lower, xmax = Upper), height = 0, color = "black", size = 1) +
  geom_vline(xintercept = 0, linetype = "dotted", color = "black") +
```

```r
  geom_point(size = 3, shape = 21, fill = "white", color = "black") +
  coord_cartesian(xlim = c(-1, 7)) +
  scale_y_discrete(labels = function(x) gsub(":", " - ", x)) +
  theme_classic() +
  xlab("Coefficient Estimate") +
  ylab("") +
  ggtitle("SMR - Model coefficients")
```

```
## Warning: Using the `size` aesthetic with geom_path was deprecated in ggplot2 3.4.0.
## i Please use the `linewidth` aesthetic instead.
## This warning is displayed once every 8 hours.
## Call `lifecycle::last_lifecycle_warnings()` to see where this warning was
## generated.
```

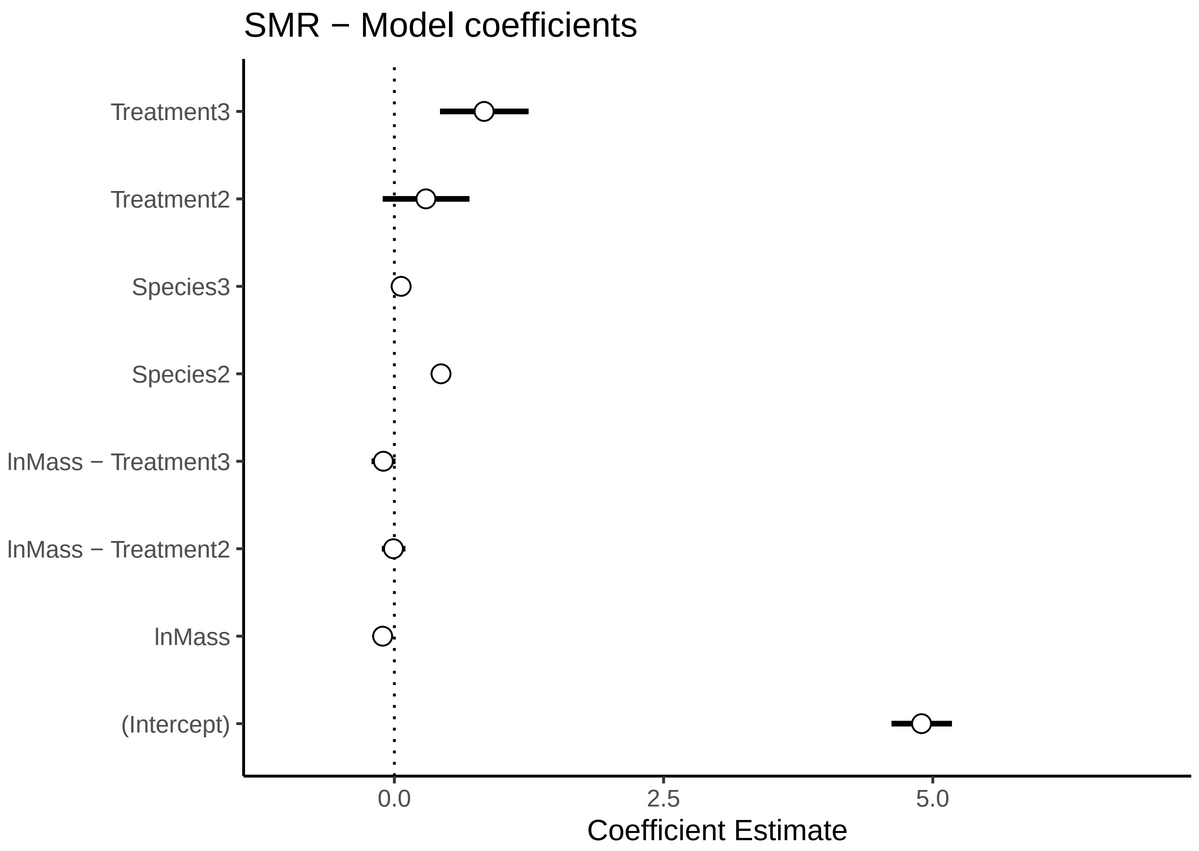


```r
# perform ANOVA
anova(smr_model12)
```

```
## Analysis of Variance Table
##
## Response: lnSMR
##                    Df Sum Sq Mean Sq  F value    Pr(>F)
## lnMass              1 0.5510 0.55102  30.4427 2.122e-07 ***
## Treatment           2 3.4269 1.71345  94.6641 < 2.2e-16 ***
## Species             2 4.3124 2.15618 119.1237 < 2.2e-16 ***
## lnMass:Treatment    2 0.0709 0.03545   1.9586    0.1457
## Residuals         116 2.0996 0.01810
## ---
## Signif. codes:  0 '***' 0.001 '**' 0.01 '*' 0.05 '.' 0.1 ' ' 1
```

```r
# perform planned contrasts - treatment differences within species
smr_NL_emmeans <- emmeans(smr_model12, ~ Treatment, at = list(lnMass = 3.9703, Species = "1"))
smr_NL_contrasts <- pairs(smr_NL_emmeans, adjust = "none")
smr_NL_summary <- summary(smr_NL_contrasts)
smr_NL_p_values <- smr_NL_summary$p.value
smr_NL_emmeans
```

```
##  Treatment emmean     SE  df lower.CL upper.CL
##  1           4.46 0.0279 116     4.41     4.52
##  2           4.72 0.0297 116     4.66     4.78
##  3           4.89 0.0300 116     4.83     4.95
##
## Confidence level used: 0.95
```

```r
smr_AT_emmeans <- emmeans(smr_model12, ~ Treatment, at = list(lnMass = 3.9512, Species = "2"))
smr_AT_contrasts <- pairs(smr_AT_emmeans, adjust = "none")
smr_AT_summary <- summary(smr_AT_contrasts)
smr_AT_p_values <- smr_AT_summary$p.value
smr_AT_emmeans
```

```
##  Treatment emmean     SE  df lower.CL upper.CL
##  1           4.90 0.0259 116     4.85     4.95
##  2           5.16 0.0298 116     5.10     5.21
##  3           5.33 0.0303 116     5.27     5.39
##
## Confidence level used: 0.95
```

```r
smr_CS_emmeans <- emmeans(smr_model12, ~ Treatment, at = list(lnMass = 3.5553, Species = "3"))
smr_CS_contrasts <- pairs(smr_CS_emmeans, adjust = "none")
smr_CS_summary <- summary(smr_CS_contrasts)
smr_CS_p_values <- smr_CS_summary$p.value
smr_CS_emmeans
```

```
##  Treatment emmean     SE  df lower.CL upper.CL
##  1           4.57 0.0279 116     4.52     4.63
##  2           4.83 0.0272 116     4.78     4.89
##  3           5.04 0.0277 116     4.99     5.10
##
## Confidence level used: 0.95
```

```r
# perform FDR correction on all contrasts
smr_p_values <- c(smr_NL_p_values, smr_AT_p_values, smr_CS_p_values)
smr_fdr_corrected_p <- p.adjust(smr_p_values, method = "fdr")

smr_contrast_names <- c("NL winter - NL summer","NL winter - NL MHW","NL summer - NL MHW",
                        "AT winter - AT summer","AT winter - AT MHW","AT summer - AT MHW",
                        "CS winter - CS summer","CS winter - CS MHW","CS summer - CS MHW")
smr_fdr_corrected_p_named <- setNames(smr_fdr_corrected_p, smr_contrast_names)
smr_fdr_corrected_p_named
```

```
## NL winter - NL summer    NL winter - NL MHW    NL summer - NL MHW
##          4.325657e-12          4.190437e-24          4.665881e-06
## AT winter - AT summer    AT winter - AT MHW    AT summer - AT MHW
##          2.954273e-12          1.911044e-24          3.031124e-06
## CS winter - CS summer    CS winter - CS MHW    CS summer - CS MHW
##          2.138080e-13          7.948805e-28          5.150297e-10
```

```r
# forest plot of planned contrasts - treatment differences within species
smr_create_forestplot <- function(data, plot_title = NULL, show_legend = FALSE)
  {data <- data %>% filter(Treatment %in% c(1, 2, 3))
  data$lower.CL <- data$emmean - 1.96 * data$SE
  data$upper.CL <- data$emmean + 1.96 * data$SE
  data$y_axis <- c(1, 2, 3)
  color_vector <- c("blue", "orange", "red")

  ggplot(data, aes(x = emmean, xmin = lower.CL, xmax = upper.CL, y = y_axis)) +
    geom_point(aes(color = factor(Treatment)), size = 4) +
    geom_errorbarh(aes(color = factor(Treatment)), height = 0) +
    scale_color_manual(values = color_vector, name = "Treatment", labels =
    c("Winter", "Summer", "MHW")) +
    scale_y_continuous(breaks = c(1, 2, 3), labels = c("Winter", "Summer", "MHW")) +
    labs(title = plot_title, x = "lnSMR (mg O2/h/kg)", y = "") +
    theme_classic() +
    theme(legend.position = ifelse(show_legend, "right", "none")) +
    coord_cartesian(xlim = c(4.4, 5.4))}

smr_NL_forestplot_emmean <- smr_create_forestplot(data.frame(smr_NL_emmeans), plot_title="NL")
smr_AT_forestplot_emmean <- smr_create_forestplot(data.frame(smr_AT_emmeans), plot_title="AT")
smr_CS_forestplot_emmean <- smr_create_forestplot(data.frame(smr_CS_emmeans), plot_title="CS")
plot_grid(smr_NL_forestplot_emmean, smr_AT_forestplot_emmean, smr_CS_forestplot_emmean,
  nrow = 1, rel_widths = c(0.33, 0.33, 0.33))
```

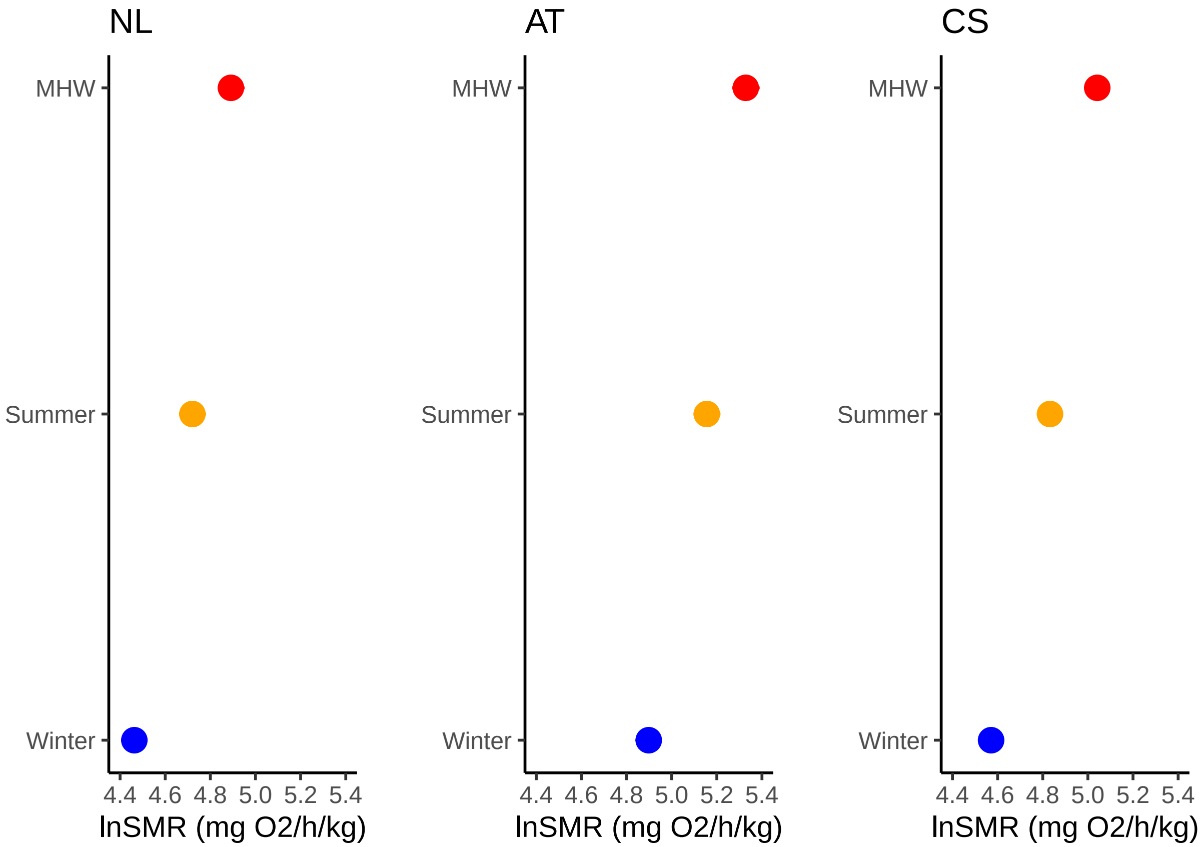

The most parsimonious linear regression model (smr_model12) statistically significantly predicted SMR (F7,116=65.99, p<0.001, adj. R2=0.787). Body mass (F1,116=30.443, p<0.001), treatment (F2,116=94.664,

p<0.001), and species (F2,116=119.124, p<0.001) added significantly to the prediction of SMR. With regards to our hypothesis, model coefficients and/or planned contrasts revealed that SMR increased in response to treatment temperature for all species (i.e. SMR in winter < summer < MHW).

Model predictions (SMR)

In this section, we use the model to predict mean SMR with confidence intervals for all species across their respective body mass ranges.

```
# predicting SMR (mean and confidence intervals)
# find the minimum and maximum body mass values for AT and NL in the dataset
nl_min_mass <- 2.6218
nl_max_mass <- 5.1249
at_min_mass <- 3.2794
at_max_mass <- 4.6238
cs_min_mass <- 2.3970
cs_max_mass <- 4.3764

# create a data frame with the desired body mass values for prediction
smr_nl_new_predictors <- data.frame(
  Species = '1',
  lnMass = rep(seq(nl_min_mass, nl_max_mass, length.out = 20), times = 3),
  Treatment = factor(rep(c(1, 2, 3), each = 20)))
smr_at_new_predictors <- data.frame(
  Species = '2',
  lnMass = rep(seq(at_min_mass, at_max_mass, length.out = 20), times = 3),
  Treatment = factor(rep(c(1, 2, 3), each = 20)))
smr_cs_new_predictors <- data.frame(
  Species = '3',
  lnMass = rep(seq(cs_min_mass, cs_max_mass, length.out = 20), times = 3),
  Treatment = factor(rep(c(1, 2, 3), each = 20)))

# use predict() to obtain predictions for each treatment level
smr_nl_predictions <- predict(smr_model12, newdata = smr_nl_new_predictors, interval = "confidence")
smr_at_predictions <- predict(smr_model12, newdata = smr_at_new_predictors, interval = "confidence")
smr_cs_predictions <- predict(smr_model12, newdata = smr_cs_new_predictors, interval = "confidence")

# add the predicted SMR values to the data frame
smr_nl_new_predictors$lnSMR <- smr_nl_predictions[,1]
smr_nl_new_predictors$lower <- smr_nl_predictions[,2]
smr_nl_new_predictors$upper <- smr_nl_predictions[,3]
smr_at_new_predictors$lnSMR <- smr_at_predictions[,1]
smr_at_new_predictors$lower <- smr_at_predictions[,2]
smr_at_new_predictors$upper <- smr_at_predictions[,3]
smr_cs_new_predictors$lnSMR <- smr_cs_predictions[,1]
smr_cs_new_predictors$lower <- smr_cs_predictions[,2]
smr_cs_new_predictors$upper <- smr_cs_predictions[,3]

# combine the predictions with the new predictors
smr_nl_predicted <- cbind(smr_nl_new_predictors, smr_nl_predictions)
smr_at_predicted <- cbind(smr_at_new_predictors, smr_at_predictions)
smr_cs_predicted <- cbind(smr_cs_new_predictors, smr_cs_predictions)

# plot the data
```

```r
smr_NL_predictplot <- ggplot() +
  geom_ribbon(data = smr_nl_predicted, aes(x = lnMass, y = lnSMR, ymin = lwr, ymax = upr,
  fill = Treatment), alpha = 0.2) +
  geom_line(data = smr_nl_predicted, aes(x = lnMass, y = lnSMR, color = Treatment),
  linewidth = 1) +
  geom_point(data = Metabolism[Metabolism$Species == '1',], aes(x = lnMass, y = lnSMR,
  color = Treatment), size = 2) +
  ggtitle("NL") +
  scale_fill_manual(values = Treatment_colors, guide = "none") +
  scale_color_manual(values = Treatment_colors, labels = Treatment_labels) +
  labs(x = "lnMass (g)", y = "lnSMR (mg O2/h/kg)") +
  theme(legend.position = "none") +
  coord_cartesian(xlim = c(2.5, 5.0), ylim = c(4.00, 5.75))

smr_AT_predictplot <- ggplot() +
  geom_ribbon(data = smr_at_predicted, aes(x = lnMass, y = lnSMR, ymin = lwr, ymax = upr,
  fill = Treatment), alpha = 0.2) +
  geom_line(data = smr_at_predicted, aes(x = lnMass, y = lnSMR, color = Treatment),
  linewidth = 1) +
  geom_point(data = Metabolism[Metabolism$Species == '2',], aes(x = lnMass, y = lnSMR,
  color = Treatment), size = 2) +
  ggtitle("AT") +
  scale_fill_manual(values = Treatment_colors, guide = "none") +
  scale_color_manual(values = Treatment_colors, labels = Treatment_labels) +
  labs(x = "lnMass (g)", y = "lnSMR (mg O2/h/kg)") +
  theme(legend.position = "none") +
  coord_cartesian(xlim = c(2.5, 5.0), ylim = c(4.00, 5.75))

smr_CS_predictplot <- ggplot() +
  geom_ribbon(data = smr_cs_predicted, aes(x = lnMass, y = lnSMR, ymin = lwr, ymax = upr,
  fill = Treatment), alpha = 0.2) +
  geom_line(data = smr_cs_predicted, aes(x = lnMass, y = lnSMR, color = Treatment),
  linewidth = 1) +
  geom_point(data = Metabolism[Metabolism$Species == '3',], aes(x = lnMass, y = lnSMR,
  color = Treatment), size = 2) +
  ggtitle("CS") +
  scale_fill_manual(values = Treatment_colors, guide = "none") +
  scale_color_manual(values = Treatment_colors, labels = Treatment_labels) +
  labs(x = "lnMass (g)", y = "lnSMR (mg O2/h/kg)") +
  theme(legend.position = "right") +
  coord_cartesian(xlim = c(2.5, 5.0), ylim = c(4.00, 5.75))

# Arrange plots in a grid
plot_grid(smr_NL_predictplot, smr_AT_predictplot, smr_CS_predictplot,
  nrow = 1, rel_widths = c(0.28, 0.28, 0.44))
```

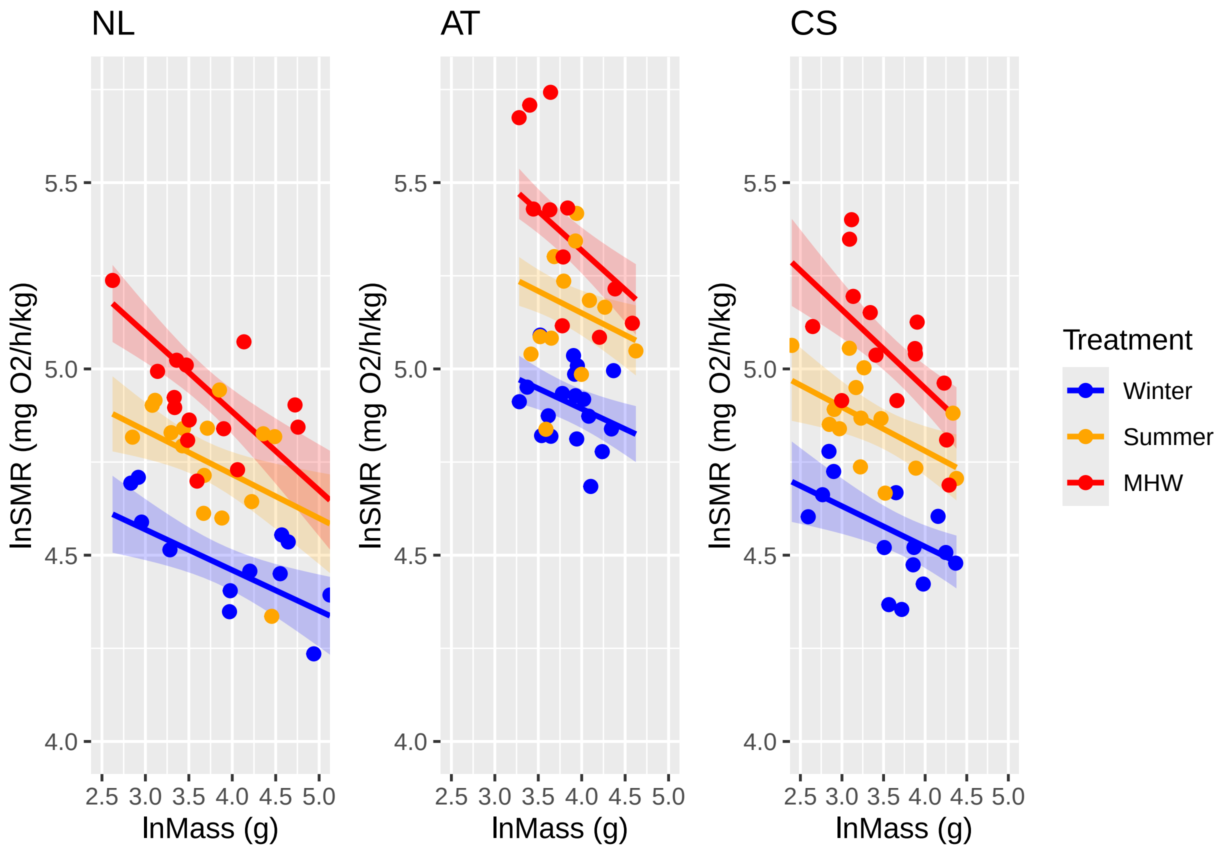

MAXIMUM METABOLIC RATE (MMR)

Data exploration (MMR)

In this section, we explore the relationship between MMR and body mass for all species of herbivores.

```
# plot untransformed dependent variable (MMR) vs. continuous independent variable (Mass)
mmr_NL_linearplot <- ggplot(Metabolism %>% filter(Species == "1"), aes(x = Mass, y = MMR,
  color = Treatment)) +
  geom_point() +
  geom_smooth(method = "lm", se = FALSE, formula = y ~ x) +
  ggtitle("NL") +
  scale_color_manual(values = Treatment_colors, labels = Treatment_labels) +
  labs(x = "Mass (g)", y = "MMR (mg O2/h/kg)") +
  theme(legend.position = "none") +
  coord_cartesian(xlim = c(0, 150), ylim = c(250, 1250))

mmr_AT_linearplot <- ggplot(Metabolism %>% filter(Species == "2"), aes(x = Mass, y = MMR,
  color = Treatment)) +
  geom_point() +
  geom_smooth(method = "lm", se = FALSE, formula = y ~ x) +
  ggtitle("AT") +
  scale_color_manual(values = Treatment_colors, labels = Treatment_labels) +
  labs(x = "Mass (g)", y = "") +
  theme(legend.position = "none") +
  coord_cartesian(xlim = c(0, 150), ylim = c(250, 1250))
```

```r
mmr_CS_linearplot <- ggplot(Metabolism %>% filter(Species == "3"), aes(x = Mass, y = MMR,
  color = Treatment)) +
  geom_point() +
  geom_smooth(method = "lm", se = FALSE, formula = y ~ x) +
  ggtitle("CS") +
  scale_color_manual(values = Treatment_colors, labels = Treatment_labels) +
  labs(x = "Mass (g)", y = "") +
  theme(legend.position = "none") +
  coord_cartesian(xlim = c(0, 150), ylim = c(250, 1250))

legend <- get_legend(
  ggplot(Metabolism %>% filter(Species == "1"), aes(x = Mass, y = MMR, color = Treatment)) +
    geom_point() +
    scale_color_manual(values = Treatment_colors, labels = Treatment_labels) +
    theme(legend.box.margin = margin(0, 0, 0, 12)))
```

```
## Warning in get_plot_component(plot, "guide-box"): Multiple components found;
## returning the first one. To return all, use `return_all = TRUE`.
```

```r
MMR_plot <- plot_grid(
  plot_grid(mmr_NL_linearplot, mmr_AT_linearplot, mmr_CS_linearplot, nrow = 1, rel_widths
  = c(1, 1, 1)), legend, ncol = 2, rel_widths = c(3, 0.5))

print(MMR_plot)
```

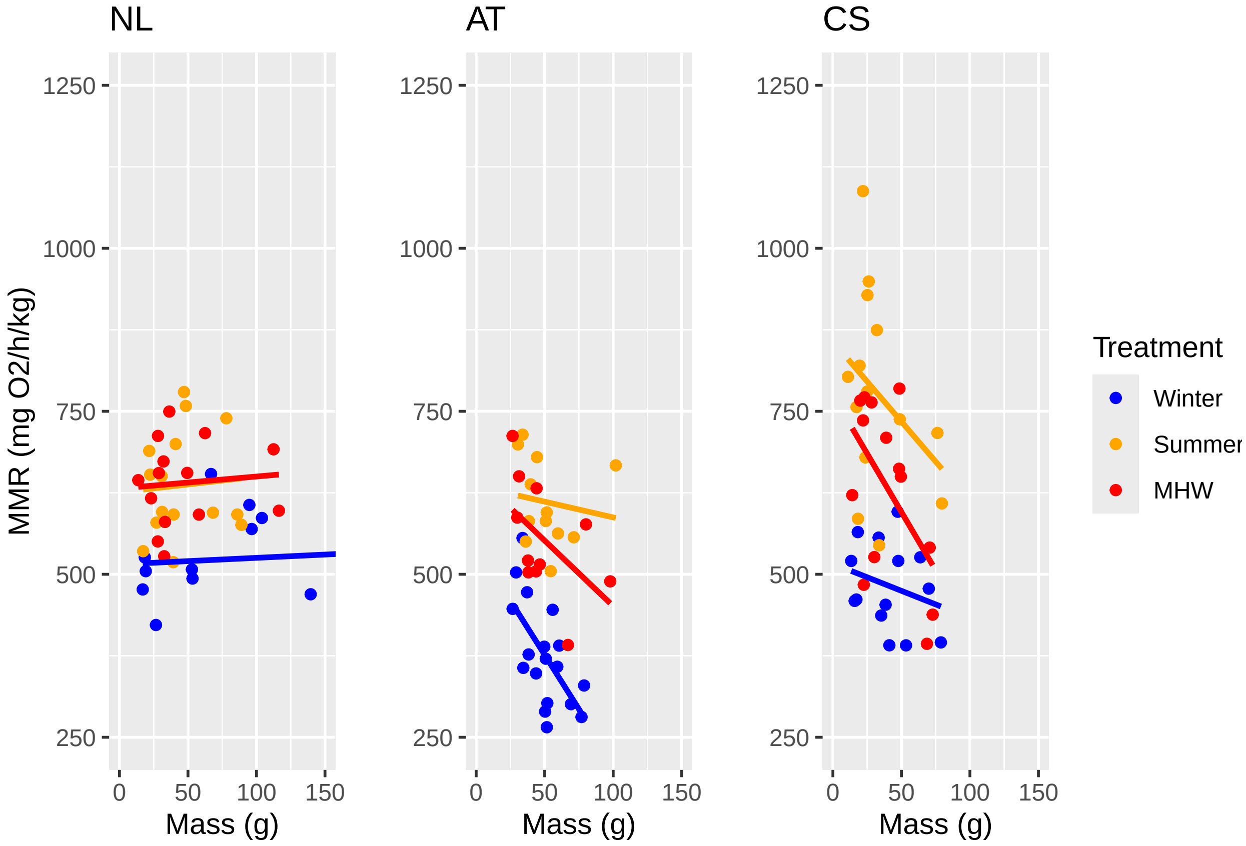


```r
# plot transformed dependent variable (lnMMR) vs. continuous independent variable (lnMass)
lnmmr_NL_linearplot <- ggplot(Metabolism %>% filter(Species == "1"), aes(x = lnMass,
```

```
  y = lnMMR, color = Treatment)) +
  geom_point() +
  geom_smooth(method = "lm", se = FALSE, formula = y ~ x) +
  ggtitle("NL") +
  scale_color_manual(values = Treatment_colors, labels = Treatment_labels) +
  labs(x = "lnMass (g)", y = "lnMMR (mg O2/h/kg)") +
  theme(legend.position = "none") +
  coord_cartesian(xlim = c(2.5, 5.0), ylim = c(5.5, 7.0))

lnmmr_AT_linearplot <- ggplot(Metabolism %>% filter(Species == "2"), aes(x = lnMass,
  y = lnMMR, color = Treatment)) +
  geom_point() +
  geom_smooth(method = "lm", se = FALSE, formula = y ~ x) +
  ggtitle("AT") +
  scale_color_manual(values = Treatment_colors, labels = Treatment_labels) +
  labs(x = "lnMass (g)", y = "") +
  theme(legend.position = "none") +
  coord_cartesian(xlim = c(2.5, 5.0), ylim = c(5.5, 7.0))

lnmmr_CS_linearplot <- ggplot(Metabolism %>% filter(Species == "3"), aes(x = lnMass,
  y = lnMMR, color = Treatment)) +
  geom_point() +
  geom_smooth(method = "lm", se = FALSE, formula = y ~ x) +
  ggtitle("CS") +
  scale_color_manual(values = Treatment_colors, labels = Treatment_labels) +
  labs(x = "lnMass (g)", y = "") +
  theme(legend.position = "none") +
  coord_cartesian(xlim = c(2.5, 5.0), ylim = c(5.5, 7.0))

legend <- get_legend(
  ggplot(Metabolism %>% filter(Species == "1"), aes(x = lnMass, y = lnMMR, color =
    Treatment)) +
    geom_point() +
    scale_color_manual(values = Treatment_colors, labels = Treatment_labels) +
    theme(legend.box.margin = margin(0, 0, 0, 12)))
```

```
## Warning in get_plot_component(plot, "guide-box"): Multiple components found;
## returning the first one. To return all, use `return_all = TRUE`.
```

```
lnMMR_plot <- plot_grid(
  plot_grid(lnmmr_NL_linearplot, lnmmr_AT_linearplot, lnmmr_CS_linearplot, nrow = 1,
  rel_widths = c(1, 1, 1)), legend, ncol = 2, rel_widths = c(3, 0.5))

print(lnMMR_plot)
```

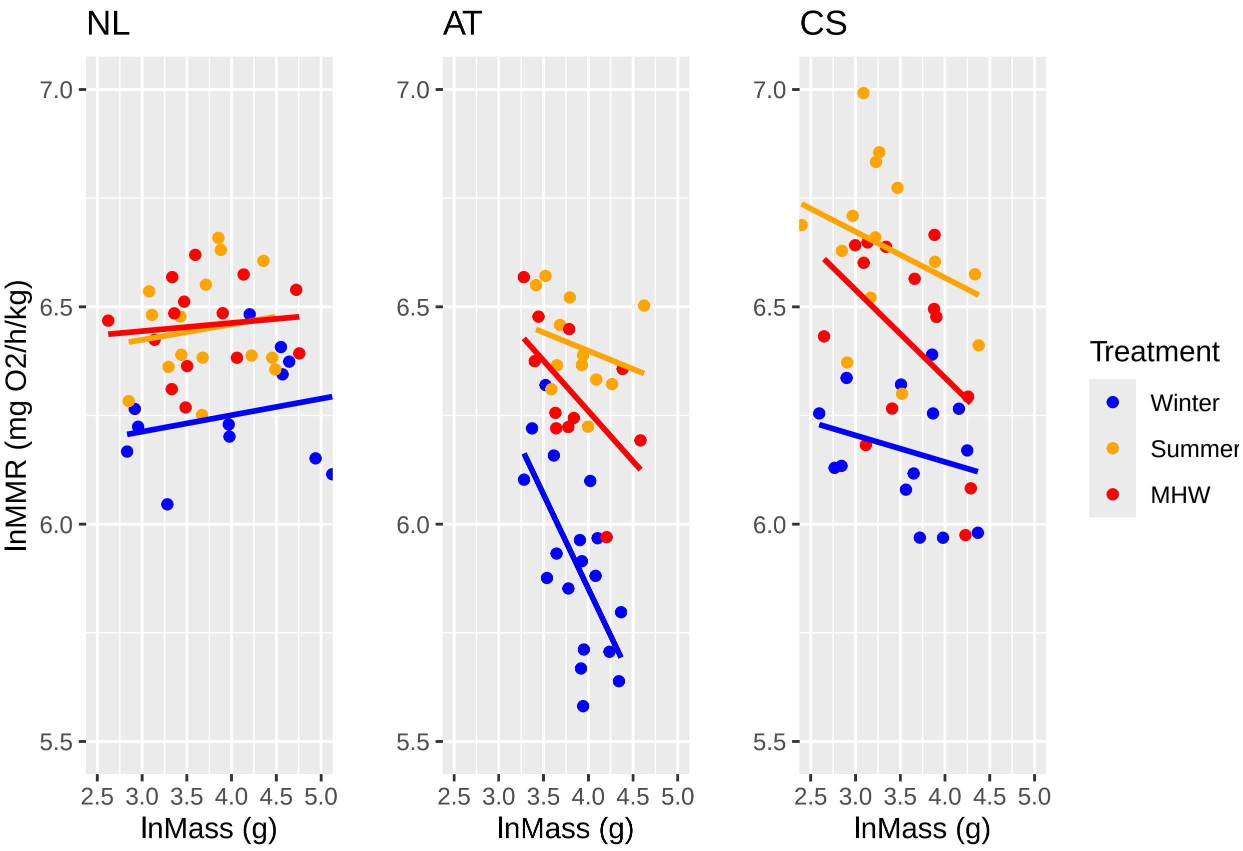

Model fitting (MMR)

In this section, we built a selection of candidate models for MMR. MMR and body mass were log transformed to best meet the assumptions underlying linear regression models.

```
# fit models
mmr_model1 = lm(lnMMR ~ 1, data = Metabolism)
mmr_model2 = lm(lnMMR ~ lnMass, data = Metabolism)
mmr_model3 = lm(lnMMR ~ Treatment, data = Metabolism)
mmr_model4 = lm(lnMMR ~ Species, data = Metabolism)
mmr_model5 = lm(lnMMR ~ lnMass + Treatment, data = Metabolism)
mmr_model6 = lm(lnMMR ~ lnMass + Species, data = Metabolism)
mmr_model7 = lm(lnMMR ~ Treatment + Species, data = Metabolism)
mmr_model8 = lm(lnMMR ~ lnMass * Treatment, data = Metabolism)
mmr_model9 = lm(lnMMR ~ lnMass * Species, data = Metabolism)
mmr_model10 = lm(lnMMR ~ Treatment * Species, data = Metabolism)
mmr_model11 = lm(lnMMR ~ lnMass + Treatment + Species, data = Metabolism)
mmr_model12 = lm(lnMMR ~ lnMass * Treatment + Species, data = Metabolism)
mmr_model13 = lm(lnMMR ~ Treatment + lnMass * Species, data = Metabolism)
mmr_model14 = lm(lnMMR ~ lnMass + Treatment * Species, data = Metabolism)
mmr_model15 = lm(lnMMR ~ lnMass * Treatment + lnMass * Species, data = Metabolism)
mmr_model16 = lm(lnMMR ~ lnMass * Treatment + Treatment * Species, data = Metabolism)
mmr_model17 = lm(lnMMR ~ lnMass * Species + Treatment * Species, data = Metabolism)
mmr_model18 = lm(lnMMR ~ lnMass * Treatment + lnMass * Species + Treatment * Species,
              data = Metabolism)
```

All of the models for MMR were fit without convergence issues.

Model selection (MMR)

In this section, we selected the best-fitting model based on Akaike's Information Criterion (AIC) from the set of candidate models (Burnham and Anderson, 2004).

```
# model selection based on AIC
mmr_aic = AIC(mmr_model1, mmr_model2, mmr_model3, mmr_model4, mmr_model5, mmr_model6,
          mmr_model7, mmr_model8, mmr_model9, mmr_model10, mmr_model11, mmr_model12,
          mmr_model13, mmr_model14, mmr_model15, mmr_model16, mmr_model17, mmr_model18)
mmr_aic = mmr_aic[order(mmr_aic$AIC), ]
mmr_aic
```

```
##             df          AIC
## mmr_model17 13 -101.0274538
## mmr_model18 15  -97.9509398
## mmr_model13  9  -92.2749178
## mmr_model14 11  -88.7197163
## mmr_model15 11  -88.6163997
## mmr_model16 13  -85.6418323
## mmr_model10 10  -84.7925514
## mmr_model11  7  -78.3399161
## mmr_model7   6  -75.9444277
## mmr_model12  9  -74.9428711
## mmr_model5   5  -53.6444859
## mmr_model8   7  -50.6801471
## mmr_model3   4  -47.1611167
## mmr_model9   7   -2.2966602
## mmr_model6   5    0.6109463
## mmr_model4   4    5.1469585
## mmr_model2   3   17.8364956
## mmr_model1   2   26.2545632
```

Model checking (MMR)

In this section, we checked the primary assumptions of linear regression models (i.e. normality, homoscedasticity and outliers).

Assumption of normality

```
# check assumption of normality for top four models
# q-q plot
plot(mmr_model17, which = 2)
```

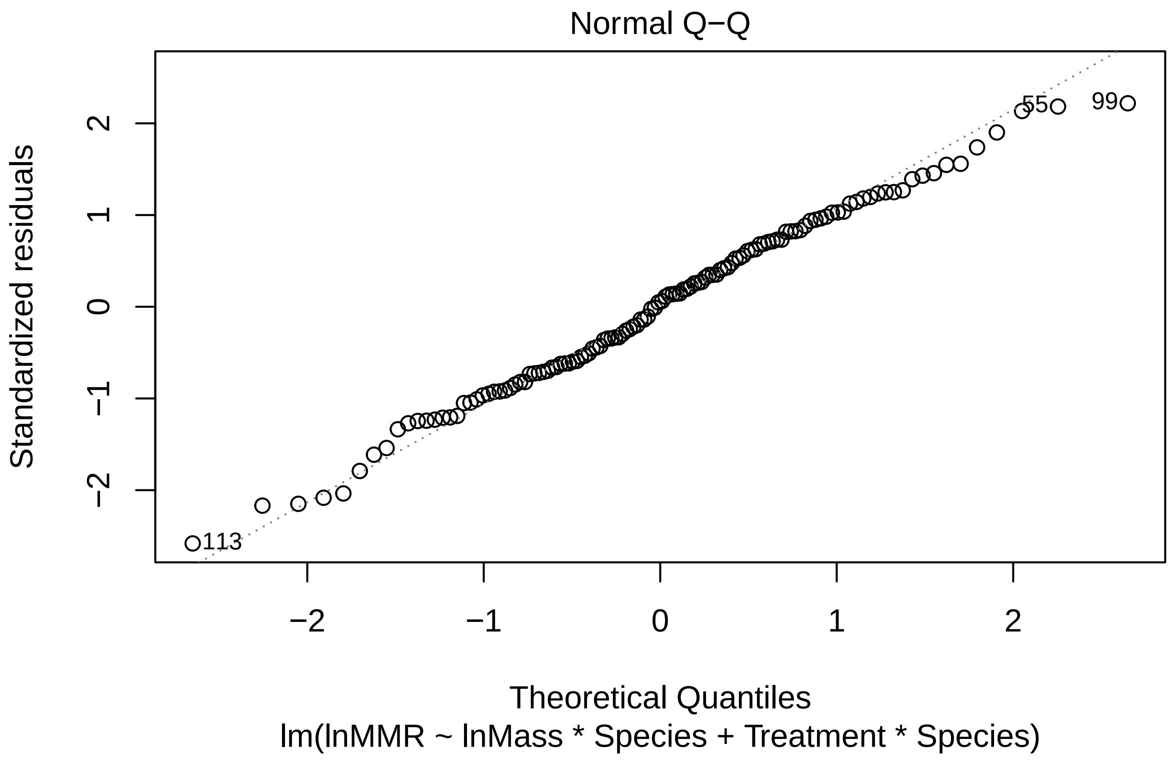


```
# shapiro-wilk test
shapiro.test(resid(mmr_model17))
```

```
##
##  Shapiro-Wilk normality test
##
## data:  resid(mmr_model17)
## W = 0.99168, p-value = 0.6691
```

Assumption of homoscedasticity

```
# check assumption of homoscedasticity for top four models
# plot residuals vs. fitted, plot standardized residuals vs. fitted
par(mfrow = c(2, 2))
plot(mmr_model17, which = 1, main = "Residuals vs. fitted")
plot(mmr_model17, which = 3, main = "Std. Residuals vs. fitted")
```

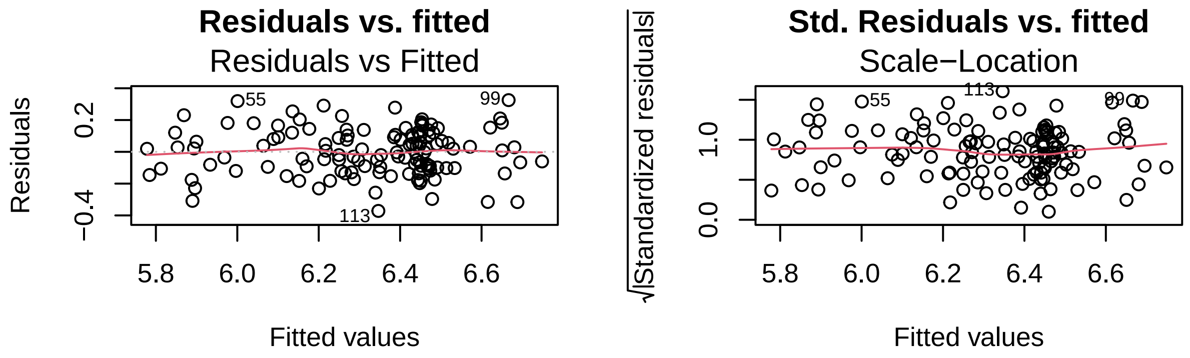

Outliers

```
# check for outliers in top four models
# plot studentized residuals, cook's distance, and leverage
mmra_stud.resid <- rstudent(mmr_model17)
par(mfrow = c(1, 3))
plot(mmra_stud.resid, main = "Studentized residuals")
plot(mmr_model17, which = 4, main = "Cook's distance")
plot(mmr_model17, which = 5, main = "Leverage")
```

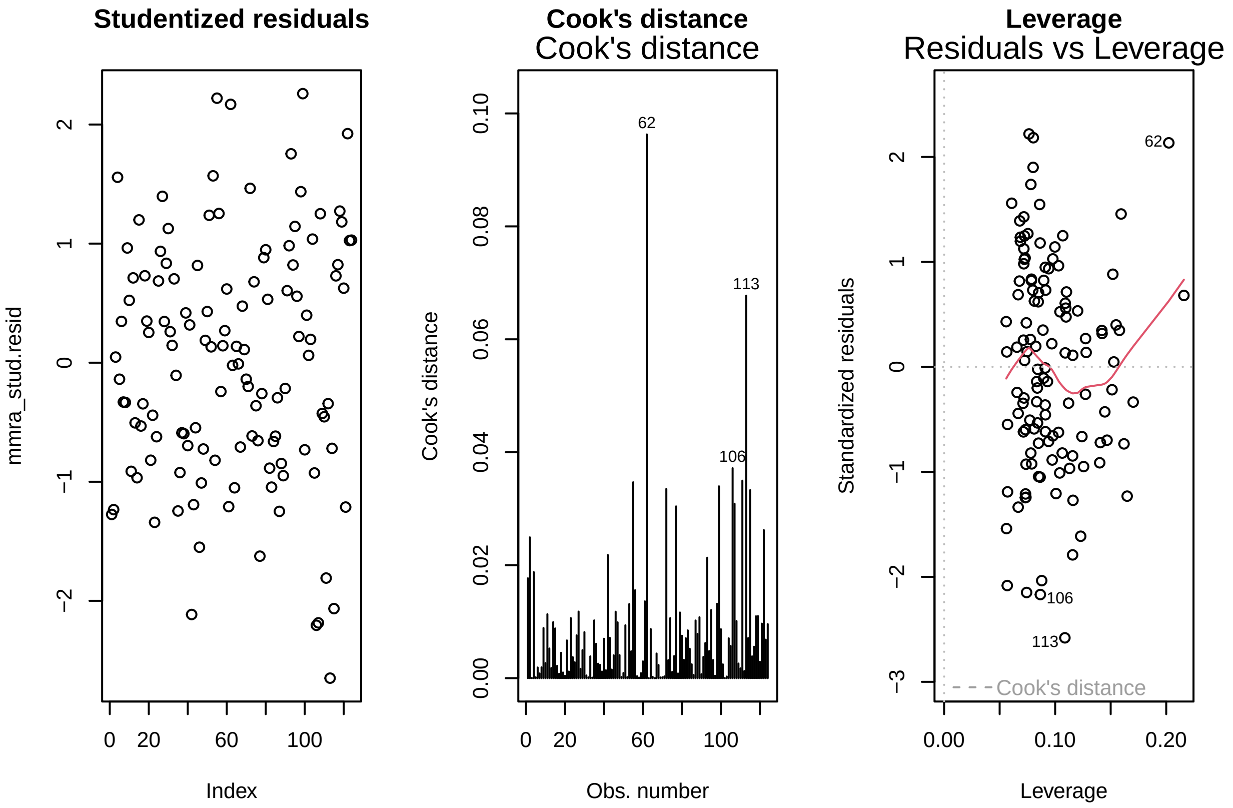

We chose to proceed with mmr_model17 based on the fact that it had the lowest AIC and best met the assumptions of normality (based on q-q plots and Shapiro-Wilk test statistic), homoscedasticity (based on residuals vs. fitted values, standardized residuals vs. fitted values) and had no significant outliers (studentized residuals between -3 and 3, cook's distances were below 1, and/or leverage was less than 0.5).

Model inference (MMR)

In this section, we inspected the values of the coefficients and the corresponding confidence intervals for body mass, treatment (1 = winter, 2 = summer, 3 = MHW), species (1= NL, 2 = AT, 3 = CS), the body mass:species interaction, and treatment:species interaction.

```
# generate model summary and confidence interval
summary(mmr_model17)
```

```
##
## Call:
## lm(formula = lnMMR ~ lnMass * Species + Treatment * Species,
##     data = Metabolism)
##
## Residuals:
```

```
##       Min       1Q   Median       3Q      Max
## -0.37168 -0.10209  0.00784  0.10648  0.32531
##
## Coefficients:
##                     Estimate Std. Error t value Pr(>|t|)
## (Intercept)          6.12350    0.16053  38.144  < 2e-16 ***
## lnMass               0.03181    0.03862   0.824 0.411936
## Species2             0.80535    0.31745   2.537 0.012560 *
## Species3             0.46966    0.22849   2.055 0.042156 *
## Treatment2           0.20784    0.06018   3.454 0.000781 ***
## Treatment3           0.21644    0.06131   3.531 0.000603 ***
## lnMass:Species2     -0.29533    0.08018  -3.683 0.000356 ***
## lnMass:Species3     -0.15046    0.05859  -2.568 0.011543 *
## Species2:Treatment2  0.29386    0.08279   3.549 0.000565 ***
## Species3:Treatment2  0.23198    0.08400   2.762 0.006722 **
## Species2:Treatment3  0.16329    0.08473   1.927 0.056483 .
## Species3:Treatment3  0.03861    0.08416   0.459 0.647268
## ---
## Signif. codes:  0 '***' 0.001 '**' 0.01 '*' 0.05 '.' 0.1 ' ' 1
##
## Residual standard error: 0.1526 on 112 degrees of freedom
## Multiple R-squared:    0.7,  Adjusted R-squared:  0.6705
## F-statistic: 23.75 on 11 and 112 DF,  p-value: < 2.2e-16
```

```
confint(mmr_model17)
```

```
##                            2.5 %      97.5 %
## (Intercept)          5.805423623  6.44158197
## lnMass              -0.044716118  0.10833093
## Species2             0.176354055  1.43434519
## Species3             0.016937299  0.92239134
## Treatment2           0.088606806  0.32707365
## Treatment3           0.094973662  0.33791228
## lnMass:Species2     -0.454194173 -0.13646151
## lnMass:Species3     -0.266553099 -0.03437444
## Species2:Treatment2  0.129822145  0.45789548
## Species3:Treatment2  0.065547561  0.39841447
## Species2:Treatment3 -0.004586252  0.33115872
## Species3:Treatment3 -0.128143006  0.20536982
```

```
# create a forest plot for model coefficients and confidence intervals
mmr_forestplot_coef <- data.frame(
  Variable = rownames(summary(mmr_model17)$coefficients),
  Estimate = summary(mmr_model17)$coefficients[, 1],
  Lower = confint(mmr_model17)[, 1],
  Upper = confint(mmr_model17)[, 2])

ggplot(mmr_forestplot_coef, aes(x = Estimate, y = Variable)) +
  geom_errorbarh(aes(xmin = Lower, xmax = Upper), height = 0, color = "black", size = 1) +
  geom_vline(xintercept = 0, linetype = "dotted", color = "black") +
  geom_point(size = 3, shape = 21, fill = "white", color = "black") +
  coord_cartesian(xlim = c(-1, 7)) +
  scale_y_discrete(labels = function(x) gsub(":", " - ", x)) +
  theme_classic() +
  xlab("Coefficient Estimate") +
```

```
  ylab("") +
  ggtitle("MMR - Model coefficients")
```

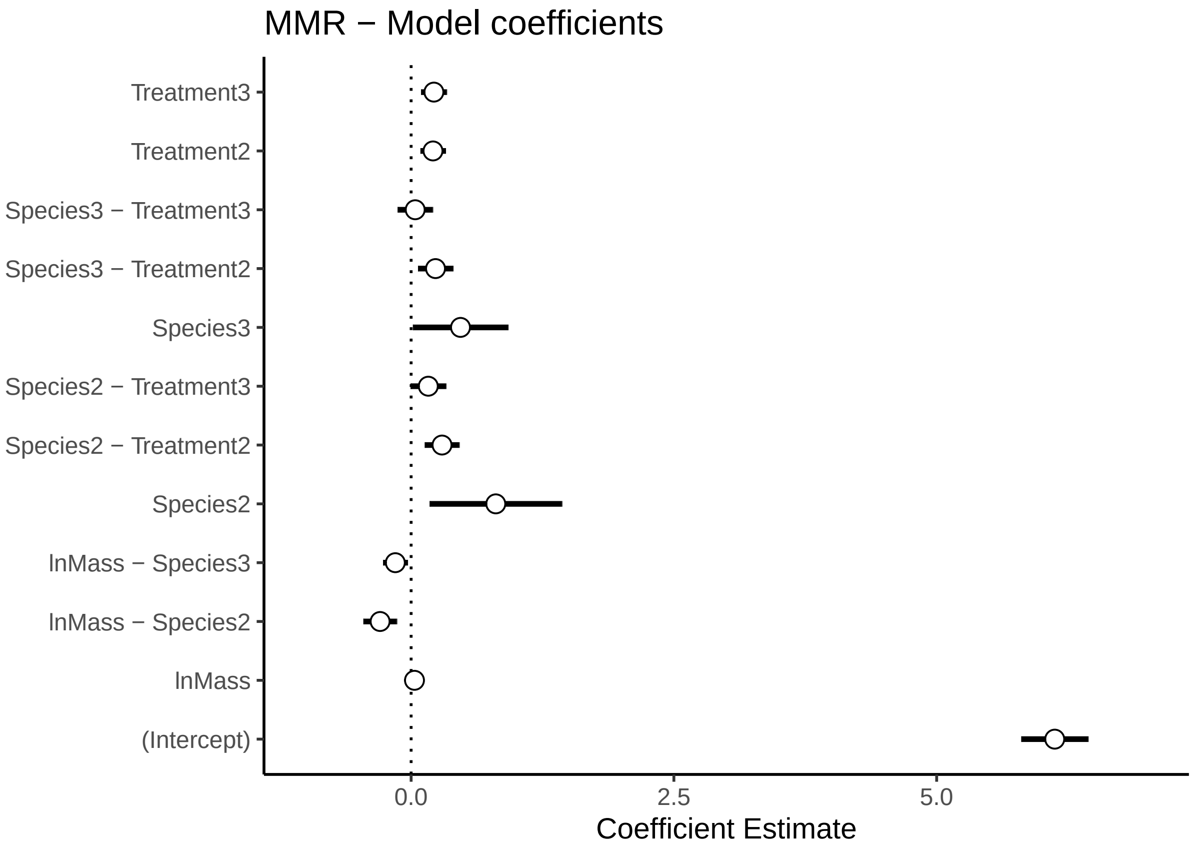


```
# perform ANOVA
anova(mmr_model17)
```

```
## Analysis of Variance Table
##
## Response: lnMMR
##                    Df Sum Sq Mean Sq F value    Pr(>F)
## lnMass              1 0.7001 0.70007 30.0821 2.596e-07 ***
## Species             2 1.2566 0.62831 26.9985 2.695e-10 ***
## Treatment           2 3.2830 1.64149 70.5353 < 2.2e-16 ***
## lnMass:Species      2 0.4643 0.23214  9.9753 0.0001031 ***
## Species:Treatment   4 0.3770 0.09426  4.0503 0.0041833 **
## Residuals         112 2.6065 0.02327
## ---
## Signif. codes:  0 '***' 0.001 '**' 0.01 '*' 0.05 '.' 0.1 ' ' 1
```

```
# perform planned contrasts - treatment differences within species
mmr_NL_emmeans <- emmeans(mmr_model17, ~ Treatment, at = list(lnMass = 3.9703, Species = "1"))
mmr_NL_contrasts <- pairs(mmr_NL_emmeans, adjust = "none")
mmr_NL_summary <- summary(mmr_NL_contrasts)
mmr_NL_p_values <- mmr_NL_summary$p.value
mmr_NL_emmeans
```

```
##  Treatment emmean     SE  df lower.CL upper.CL
##  1           6.25 0.0440 112     6.16     6.34
```

```
## 2          6.46 0.0407 112     6.38     6.54
## 3          6.47 0.0424 112     6.38     6.55
##
## Confidence level used: 0.95
```

```
mmr_AT_emmeans <- emmeans(mmr_model17, ~ Treatment, at = list(lnMass = 3.9512, Species = "2"))
mmr_AT_contrasts <- pairs(mmr_AT_emmeans, adjust = "none")
mmr_AT_summary <- summary(mmr_AT_contrasts)
mmr_AT_p_values <- mmr_AT_summary$p.value
mmr_AT_emmeans
```

```
##  Treatment emmean     SE  df lower.CL upper.CL
##  1           5.89 0.0365 112     5.82     5.96
##  2           6.39 0.0444 112     6.30     6.48
##  3           6.27 0.0470 112     6.17     6.36
##
## Confidence level used: 0.95
```

```
mmr_CS_emmeans <- emmeans(mmr_model17, ~ Treatment, at = list(lnMass = 3.5553, Species = "3"))
mmr_CS_contrasts <- pairs(mmr_CS_emmeans, adjust = "none")
mmr_CS_summary <- summary(mmr_CS_contrasts)
mmr_CS_p_values <- mmr_CS_summary$p.value
mmr_CS_emmeans
```

```
##  Treatment emmean     SE  df lower.CL upper.CL
##  1           6.17 0.0408 112     6.09     6.25
##  2           6.61 0.0419 112     6.53     6.69
##  3           6.43 0.0408 112     6.35     6.51
##
## Confidence level used: 0.95
```

```
# perform FDR correction on all contrasts
mmr_p_values <- c(mmr_NL_p_values, mmr_AT_p_values, mmr_CS_p_values)
mmr_fdr_corrected_p <- p.adjust(mmr_p_values, method = "fdr")

mmr_contrast_names <- c("NL winter - NL summer","NL winter - NL MHW","NL summer - NL MHW",
                        "AT winter - AT summer","AT winter - AT MHW","AT summer - AT MHW",
                        "CS winter - CS summer","CS winter - CS MHW","CS summer - CS MHW")
mmr_fdr_corrected_p_named <- setNames(mmr_fdr_corrected_p, mmr_contrast_names)
mmr_fdr_corrected_p_named
```

```
## NL winter - NL summer    NL winter - NL MHW    NL summer - NL MHW
##          1.171892e-03          1.085496e-03          8.796786e-01
## AT winter - AT summer    AT winter - AT MHW    AT summer - AT MHW
##          1.523772e-13          7.159678e-09          6.583276e-02
## CS winter - CS summer    CS winter - CS MHW    CS summer - CS MHW
##          7.116584e-11          5.092852e-05          2.625213e-03
```

```
# forest plot of planned contrasts - treatment differences within species
mmr_create_forestplot <- function(data, plot_title = NULL, show_legend = FALSE)
  {data <- data %>% filter(Treatment %in% c(1, 2, 3))
  data$lower.CL <- data$emmean - 1.96 * data$SE
  data$upper.CL <- data$emmean + 1.96 * data$SE
  data$y_axis <- c(1, 2, 3)
  color_vector <- c("blue", "orange", "red")

  ggplot(data, aes(x = emmean, xmin = lower.CL, xmax = upper.CL, y = y_axis)) +
```

```
    geom_point(aes(color = factor(Treatment)), size = 4) +
    geom_errorbarh(aes(color = factor(Treatment)), height = 0) +
    scale_color_manual(values = color_vector, name = "Treatment", labels =
    c("Winter", "Summer", "MHW")) +
    scale_y_continuous(breaks = c(1, 2, 3), labels = c("Winter", "Summer", "MHW")) +
    labs(title = plot_title, x = "lnMMR (mg O2/h/kg)", y = "") +
    theme_classic() +
    theme(legend.position = ifelse(show_legend, "right", "none")) +
    coord_cartesian(xlim = c(5.5, 7.0))}

mmr_NL_forestplot_emmean <- mmr_create_forestplot(data.frame(mmr_NL_emmeans), plot_title="NL")
mmr_AT_forestplot_emmean <- mmr_create_forestplot(data.frame(mmr_AT_emmeans), plot_title="AT")
mmr_CS_forestplot_emmean <- mmr_create_forestplot(data.frame(mmr_CS_emmeans), plot_title="CS")
plot_grid(mmr_NL_forestplot_emmean, mmr_AT_forestplot_emmean, mmr_CS_forestplot_emmean,
  nrow = 1, rel_widths = c(0.33, 0.33, 0.33))
```

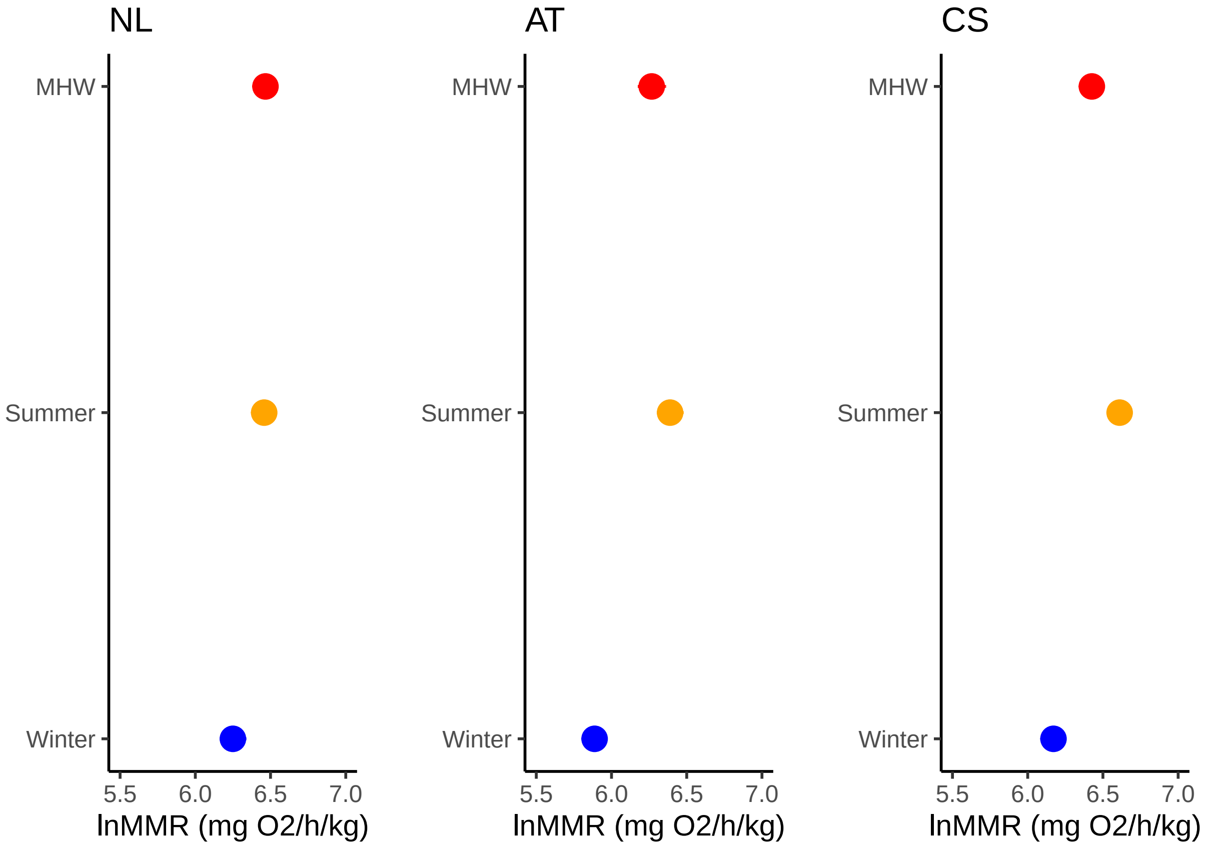

The most parsimonious linear regression model (mmr_model17) statistically significantly predicted MMR ($F_{11,112}$=23.750, p<0.001, adj. R^2=0.671). The interaction between body mass and species ($F_{2,112}$=9.975, p<0.001) and the interaction between species and treatment ($F_{4,112}$=4.050, p=0.004) added significantly to the prediction of MMR. With regards to our hypothesis, model coefficients and/or planned contrasts revealed that MMR increased from winter to summer in all species, and then from summer to MHW it either plateaued (NL and AT) or decreased (CS).

Model predictions (MMR)

In this section, we use the model to predict mean MMR with confidence intervals for all species across their respective body mass ranges.

```r
# predicting MMR (mean and confidence intervals)
# find the minimum and maximum body mass values for AT and NL in the dataset
nl_min_mass <- 2.6218
nl_max_mass <- 5.1249
at_min_mass <- 3.2794
at_max_mass <- 4.6238
cs_min_mass <- 2.3970
cs_max_mass <- 4.3764

# create a data frame with the desired body mass values for prediction
mmr_nl_new_predictors <- data.frame(
  Species = '1',
  lnMass = rep(seq(nl_min_mass, nl_max_mass, length.out = 20), times = 3),
  Treatment = factor(rep(c(1, 2, 3), each = 20)))
mmr_at_new_predictors <- data.frame(
  Species = '2',
  lnMass = rep(seq(at_min_mass, at_max_mass, length.out = 20), times = 3),
  Treatment = factor(rep(c(1, 2, 3), each = 20)))
mmr_cs_new_predictors <- data.frame(
  Species = '3',
  lnMass = rep(seq(cs_min_mass, cs_max_mass, length.out = 20), times = 3),
  Treatment = factor(rep(c(1, 2, 3), each = 20)))

# use predict() to obtain predictions for each treatment level
mmr_nl_predictions <- predict(mmr_model17, newdata = mmr_nl_new_predictors, interval = "confidence")
mmr_at_predictions <- predict(mmr_model17, newdata = mmr_at_new_predictors, interval = "confidence")
mmr_cs_predictions <- predict(mmr_model17, newdata = mmr_cs_new_predictors, interval = "confidence")

# add the predicted MMR values to the data frame
mmr_nl_new_predictors$lnMMR <- mmr_nl_predictions[,1]
mmr_nl_new_predictors$lower <- mmr_nl_predictions[,2]
mmr_nl_new_predictors$upper <- mmr_nl_predictions[,3]
mmr_at_new_predictors$lnMMR <- mmr_at_predictions[,1]
mmr_at_new_predictors$lower <- mmr_at_predictions[,2]
mmr_at_new_predictors$upper <- mmr_at_predictions[,3]
mmr_cs_new_predictors$lnMMR <- mmr_cs_predictions[,1]
mmr_cs_new_predictors$lower <- mmr_cs_predictions[,2]
mmr_cs_new_predictors$upper <- mmr_cs_predictions[,3]

# combine the predictions with the new predictors
mmr_nl_predicted <- cbind(mmr_nl_new_predictors, mmr_nl_predictions)
mmr_at_predicted <- cbind(mmr_at_new_predictors, mmr_at_predictions)
mmr_cs_predicted <- cbind(mmr_cs_new_predictors, mmr_cs_predictions)

# plot the data
mmr_NL_predictplot <- ggplot() +
  geom_ribbon(data = mmr_nl_predicted, aes(x = lnMass, y = lnMMR, ymin = lwr, ymax = upr,
  fill = Treatment), alpha = 0.2) +
  geom_line(data = mmr_nl_predicted, aes(x = lnMass, y = lnMMR, color = Treatment),
  linewidth = 1) +
  geom_point(data = Metabolism[Metabolism$Species == '1',], aes(x = lnMass, y = lnMMR,
  color = Treatment), size = 2) +
  ggtitle("NL") +
```

```r
  scale_fill_manual(values = Treatment_colors, guide = "none") +
  scale_color_manual(values = Treatment_colors, labels = Treatment_labels) +
  labs(x = "lnMass (g)", y = "lnMMR (mg O2/h/kg)") +
  theme(legend.position = "none") +
  coord_cartesian(xlim = c(2.5, 5.0), ylim = c(5.5, 7.0))

mmr_AT_predictplot <- ggplot() +
  geom_ribbon(data = mmr_at_predicted, aes(x = lnMass, y = lnMMR, ymin = lwr, ymax = upr,
  fill = Treatment), alpha = 0.2) +
  geom_line(data = mmr_at_predicted, aes(x = lnMass, y = lnMMR, color = Treatment),
  linewidth = 1) +
  geom_point(data = Metabolism[Metabolism$Species == '2',], aes(x = lnMass, y = lnMMR,
  color = Treatment), size = 2) +
  ggtitle("AT") +
  scale_fill_manual(values = Treatment_colors, guide = "none") +
  scale_color_manual(values = Treatment_colors, labels = Treatment_labels) +
  labs(x = "lnMass (g)", y = "lnMMR (mg O2/h/kg)") +
  theme(legend.position = "none") +
  coord_cartesian(xlim = c(2.5, 5.0), ylim = c(5.5, 7.0))

mmr_CS_predictplot <- ggplot() +
  geom_ribbon(data = mmr_cs_predicted, aes(x = lnMass, y = lnMMR, ymin = lwr, ymax = upr,
  fill = Treatment), alpha = 0.2) +
  geom_line(data = mmr_cs_predicted, aes(x = lnMass, y = lnMMR, color = Treatment),
  linewidth = 1) +
  geom_point(data = Metabolism[Metabolism$Species == '3',], aes(x = lnMass, y = lnMMR,
  color = Treatment), size = 2) +
  ggtitle("CS") +
  scale_fill_manual(values = Treatment_colors, guide = "none") +
  scale_color_manual(values = Treatment_colors, labels = Treatment_labels) +
  labs(x = "lnMass (g)", y = "lnMMR (mg O2/h/kg)") +
  theme(legend.position = "right") +
  coord_cartesian(xlim = c(2.5, 5.0), ylim = c(5.5, 7.0))

# Arrange plots in a grid
plot_grid(mmr_NL_predictplot, mmr_AT_predictplot, mmr_CS_predictplot,
  nrow = 1, rel_widths = c(0.28, 0.28, 0.44))
```

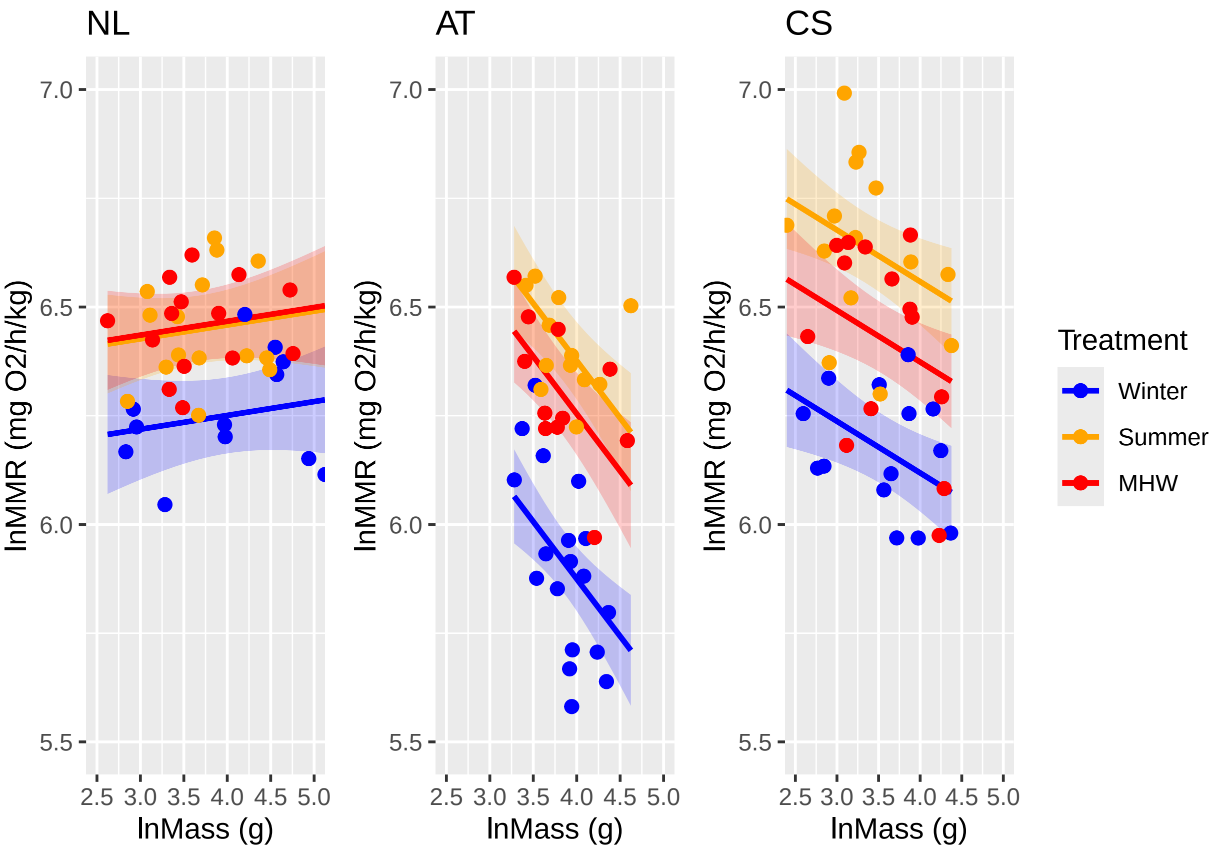

AEROBIC SCOPE (AS)

Data exploration (AS)

In this section, we explore the relationship between AS and body mass for all species of herbivores.

```
# plot untransformed dependent variable (AS) vs. continuous independent variable (Mass)
as_NL_linearplot <- ggplot(Metabolism %>% filter(Species == "1"), aes(x = Mass, y = AS,
  color = Treatment)) +
  geom_point() +
  geom_smooth(method = "lm", se = FALSE, formula = y ~ x) +
  ggtitle("NL") +
  scale_color_manual(values = Treatment_colors, labels = Treatment_labels) +
  labs(x = "Mass (g)", y = "AS (mg O2/h/kg)") +
  theme(legend.position = "none") +
  coord_cartesian(xlim = c(0, 150), ylim = c(100, 900))

as_AT_linearplot <- ggplot(Metabolism %>% filter(Species == "2"), aes(x = Mass, y = AS,
  color = Treatment)) +
  geom_point() +
  geom_smooth(method = "lm", se = FALSE, formula = y ~ x) +
  ggtitle("AT") +
  scale_color_manual(values = Treatment_colors, labels = Treatment_labels) +
  labs(x = "Mass (g)", y = "") +
  theme(legend.position = "none") +
  coord_cartesian(xlim = c(0, 150), ylim = c(100, 900))
```

```
as_CS_linearplot <- ggplot(Metabolism %>% filter(Species == "3"), aes(x = Mass, y = AS,
  color = Treatment)) +
  geom_point() +
  geom_smooth(method = "lm", se = FALSE, formula = y ~ x) +
  ggtitle("CS") +
  scale_color_manual(values = Treatment_colors, labels = Treatment_labels) +
  labs(x = "Mass (g)", y = "") +
  theme(legend.position = "none") +
  coord_cartesian(xlim = c(0, 150), ylim = c(100, 900))

legend <- get_legend(
  ggplot(Metabolism %>% filter(Species == "1"), aes(x = Mass, y = AS, color = Treatment)) +
    geom_point() +
    scale_color_manual(values = Treatment_colors, labels = Treatment_labels) +
    theme(legend.box.margin = margin(0, 0, 0, 12))
)
```

```
## Warning in get_plot_component(plot, "guide-box"): Multiple components found;
## returning the first one. To return all, use `return_all = TRUE`.
```

```
AS_plot <- plot_grid(
  plot_grid(as_NL_linearplot, as_AT_linearplot, as_CS_linearplot, nrow = 1, rel_widths
  = c(1, 1, 1)), legend, ncol = 2, rel_widths = c(3, 0.5))

print(AS_plot)
```

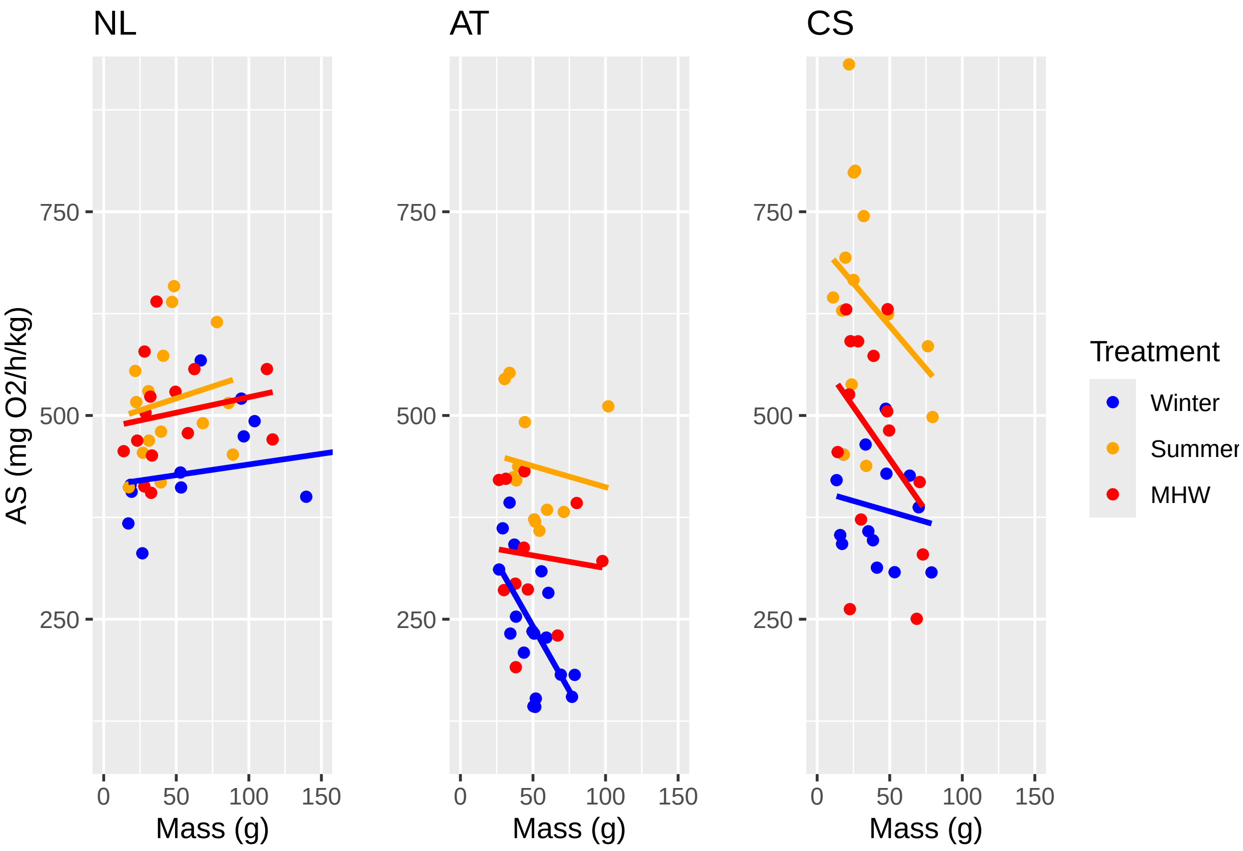


```
# plot transformed dependent variable (lnAS) vs. continuous independent variable (lnMass)
lnas_NL_linearplot <- ggplot(Metabolism %>% filter(Species == "1"), aes(x = lnMass,
```

```
  y = lnAS, color = Treatment)) +
  geom_point() +
  geom_smooth(method = "lm", se = FALSE, formula = y ~ x) +
  ggtitle("NL") +
  scale_color_manual(values = Treatment_colors, labels = Treatment_labels) +
  labs(x = "lnMass (g)", y = "lnAS (mg O2/h/kg)") +
  theme(legend.position = "none") +
  coord_cartesian(xlim = c(2.5, 5.0), ylim = c(5.0, 7.0))

lnas_AT_linearplot <- ggplot(Metabolism %>% filter(Species == "2"), aes(x = lnMass,
  y = lnAS, color = Treatment)) +
  geom_point() +
  geom_smooth(method = "lm", se = FALSE, formula = y ~ x) +
  ggtitle("AT") +
  scale_color_manual(values = Treatment_colors, labels = Treatment_labels) +
  labs(x = "lnMass (g)", y = "") +
  theme(legend.position = "none") +
  coord_cartesian(xlim = c(2.5, 5.0), ylim = c(5.0, 7.0))

lnas_CS_linearplot <- ggplot(Metabolism %>% filter(Species == "3"), aes(x = lnMass,
  y = lnAS, color = Treatment)) +
  geom_point() +
  geom_smooth(method = "lm", se = FALSE, formula = y ~ x) +
  ggtitle("CS") +
  scale_color_manual(values = Treatment_colors, labels = Treatment_labels) +
  labs(x = "lnMass (g)", y = "") +
  theme(legend.position = "none") +
  coord_cartesian(xlim = c(2.5, 5.0), ylim = c(5.0, 7.0))

legend <- get_legend(
  ggplot(Metabolism %>% filter(Species == "1"), aes(x = lnMass, y = lnAS, color =
    Treatment)) +
    geom_point() +
    scale_color_manual(values = Treatment_colors, labels = Treatment_labels) +
    theme(legend.box.margin = margin(0, 0, 0, 12)))
```

```
## Warning in get_plot_component(plot, "guide-box"): Multiple components found;
## returning the first one. To return all, use `return_all = TRUE`.
```

```
lnAS_plot <- plot_grid(
  plot_grid(lnas_NL_linearplot, lnas_AT_linearplot, lnas_CS_linearplot, nrow = 1,
  rel_widths = c(1, 1, 1)), legend, ncol = 2, rel_widths = c(3, 0.5))

print(lnAS_plot)
```

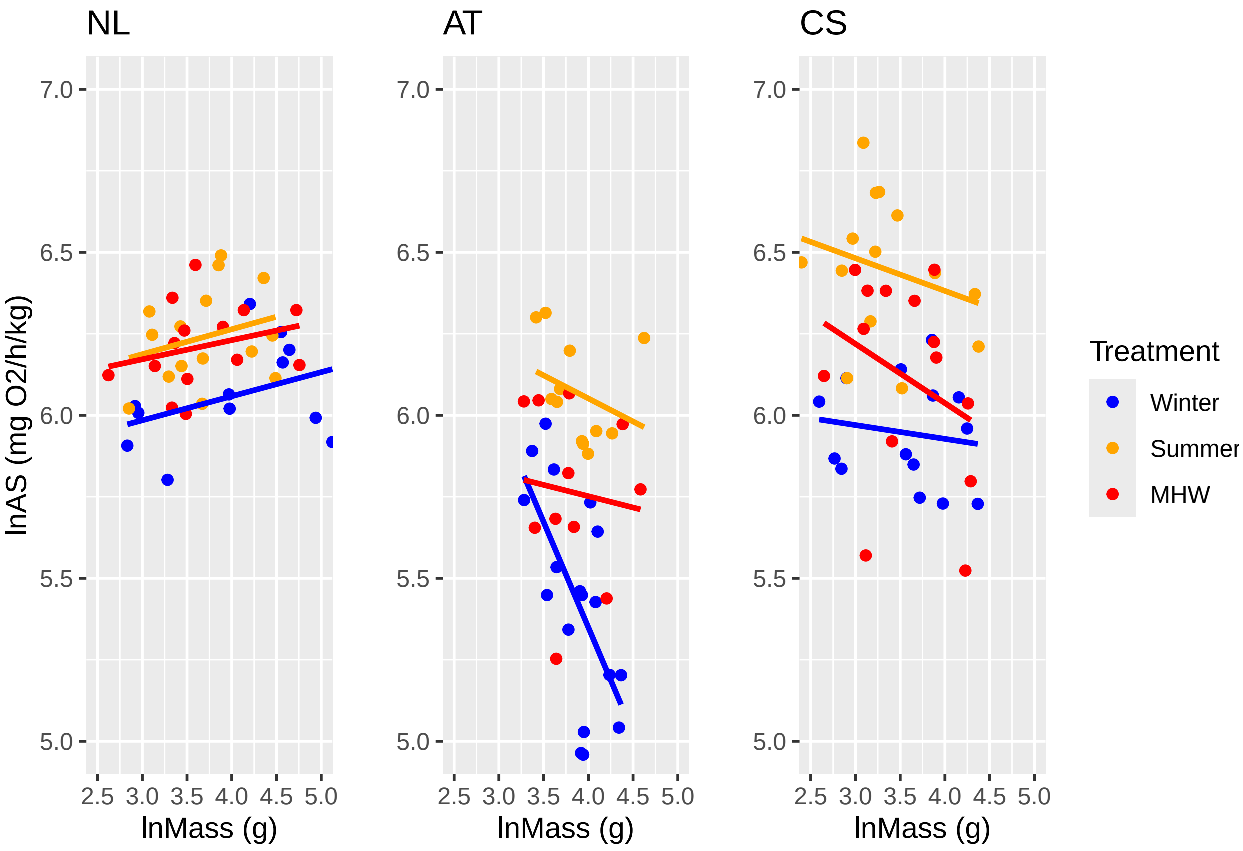

Model fitting (AS)

In this section, we built a selection of candidate models for AS. AS and body mass were log transformed to best meet the assumptions underlying linear regression models.

```
# fit models
as_model1 = lm(lnAS ~ 1, data = Metabolism)
as_model2 = lm(lnAS ~ lnMass, data = Metabolism)
as_model3 = lm(lnAS ~ Treatment, data = Metabolism)
as_model4 = lm(lnAS ~ Species, data = Metabolism)
as_model5 = lm(lnAS ~ lnMass + Treatment, data = Metabolism)
as_model6 = lm(lnAS ~ lnMass + Species, data = Metabolism)
as_model7 = lm(lnAS ~ Treatment + Species, data = Metabolism)
as_model8 = lm(lnAS ~ lnMass * Treatment, data = Metabolism)
as_model9 = lm(lnAS ~ lnMass * Species, data = Metabolism)
as_model10 = lm(lnAS ~ Treatment * Species, data = Metabolism)
as_model11 = lm(lnAS ~ lnMass + Treatment + Species, data = Metabolism)
as_model12 = lm(lnAS ~ lnMass * Treatment + Species, data = Metabolism)
as_model13 = lm(lnAS ~ Treatment + lnMass * Species, data = Metabolism)
as_model14 = lm(lnAS ~ lnMass + Treatment * Species, data = Metabolism)
as_model15 = lm(lnAS ~ lnMass * Treatment + lnMass * Species, data = Metabolism)
as_model16 = lm(lnAS ~ lnMass * Treatment + Treatment * Species, data = Metabolism)
as_model17 = lm(lnAS ~ lnMass * Species + Treatment * Species, data = Metabolism)
as_model18 = lm(lnAS ~ lnMass * Treatment + lnMass * Species + Treatment * Species,
              data = Metabolism)
```

All of the models for AS were fit without convergence issues.

Model selection (AS)

In this section, we selected the best-fitting model based on Akaike's Information Criterion (AIC) from the set of candidate models (Burnham and Anderson, 2004).

```
# model selection based on AIC
as_aic = AIC(as_model1, as_model2, as_model3, as_model4, as_model5, as_model6, as_model7,
        as_model8, as_model9, as_model10, as_model11, as_model12, as_model13, as_model14,
        as_model15, as_model16, as_model17, as_model18)
as_aic = as_aic[order(as_aic$AIC), ]
as_aic
```

```
##            df         AIC
## as_model17 13 -16.8994376
## as_model18 15 -13.2228839
## as_model10 10  -8.7699228
## as_model14 11  -8.3739195
## as_model13  9  -7.7907925
## as_model16 13  -4.4333361
## as_model15 11  -4.0271138
## as_model7   6   0.3320168
## as_model11  7   1.3531843
## as_model12  9   5.3529962
## as_model9   7  53.0779126
## as_model6   5  55.8050389
## as_model4   4  56.6824285
## as_model5   5  62.1114850
## as_model3   4  64.3531771
## as_model8   7  65.6264685
## as_model2   3 102.2460659
## as_model1   2 106.9370510
```

Model checking (AS)

In this section, we checked the primary assumptions of linear regression models (i.e. normality, homoscedasticity and outliers).

Assumption of normality

```
# check assumption of normality for top four models
# q-q plot
plot(as_model17, which = 2)
```

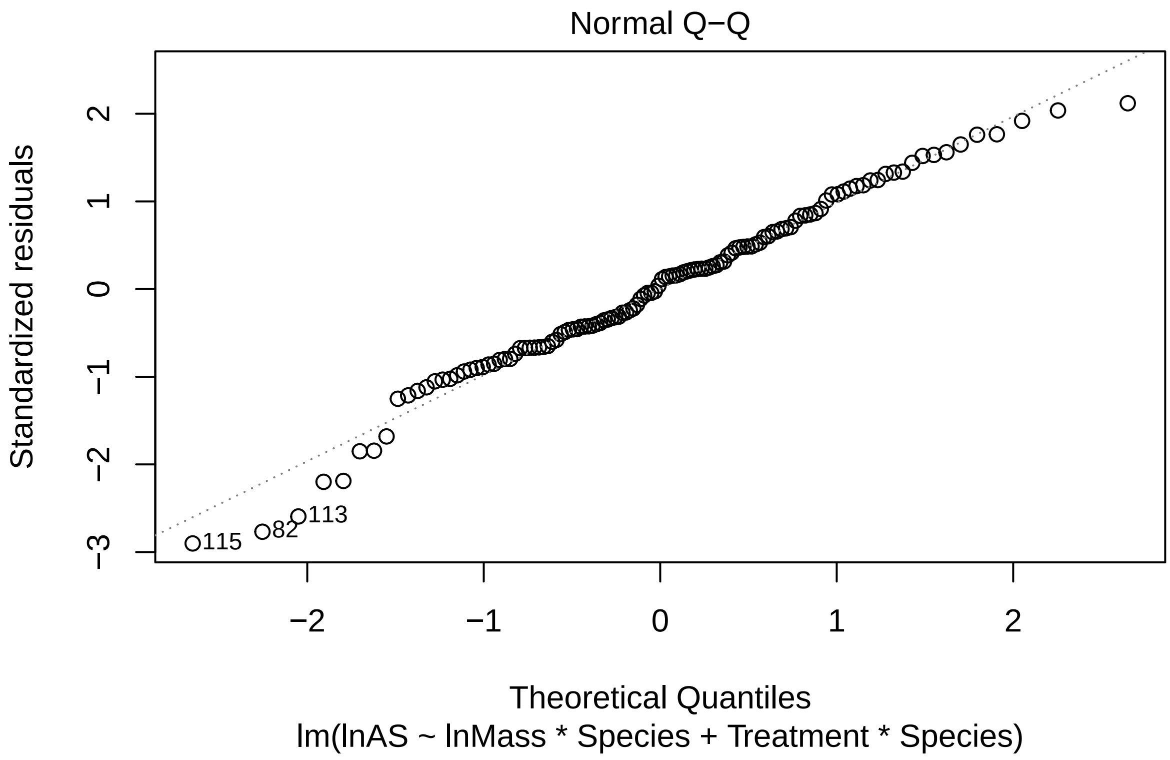


```
# shapiro-wilk test
shapiro.test(resid(as_model17))
```

```
##
##  Shapiro-Wilk normality test
##
## data:  resid(as_model17)
## W = 0.9814, p-value = 0.08556
```

Assumption of homoscedasticity

```
# check assumption of homoscedasticity for top four models
# plot residuals vs. fitted, plot standardized residuals vs. fitted
par(mfrow = c(2, 2))
plot(as_model17, which = 1, main = "Residuals vs. fitted")
plot(as_model17, which = 3, main = "Std. Residuals vs. fitted")
```

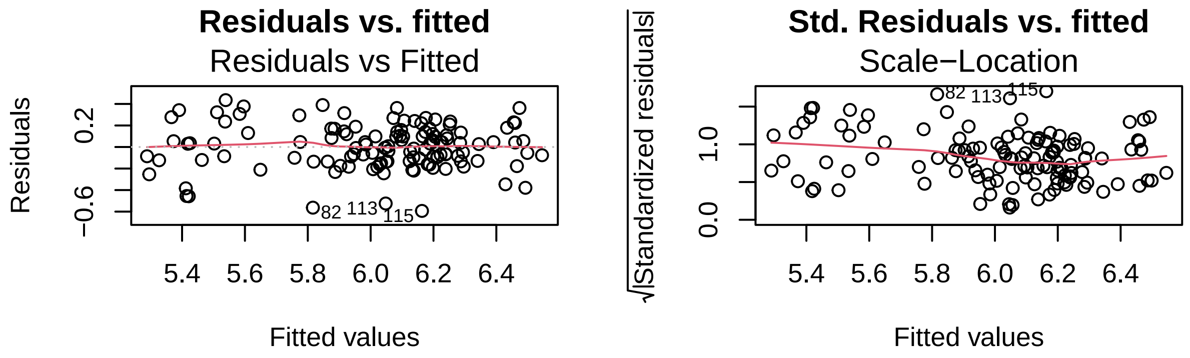

Outliers

```r
# check for outliers in top four models
# plot studentized residuals, cook's distance, and leverage
asa_stud.resid <- rstudent(as_model17)
par(mfrow = c(1, 3))
plot(asa_stud.resid, main = "Studentized residuals")
plot(as_model17, which = 4, main = "Cook's distance")
plot(as_model17, which = 5, main = "Leverage")
```

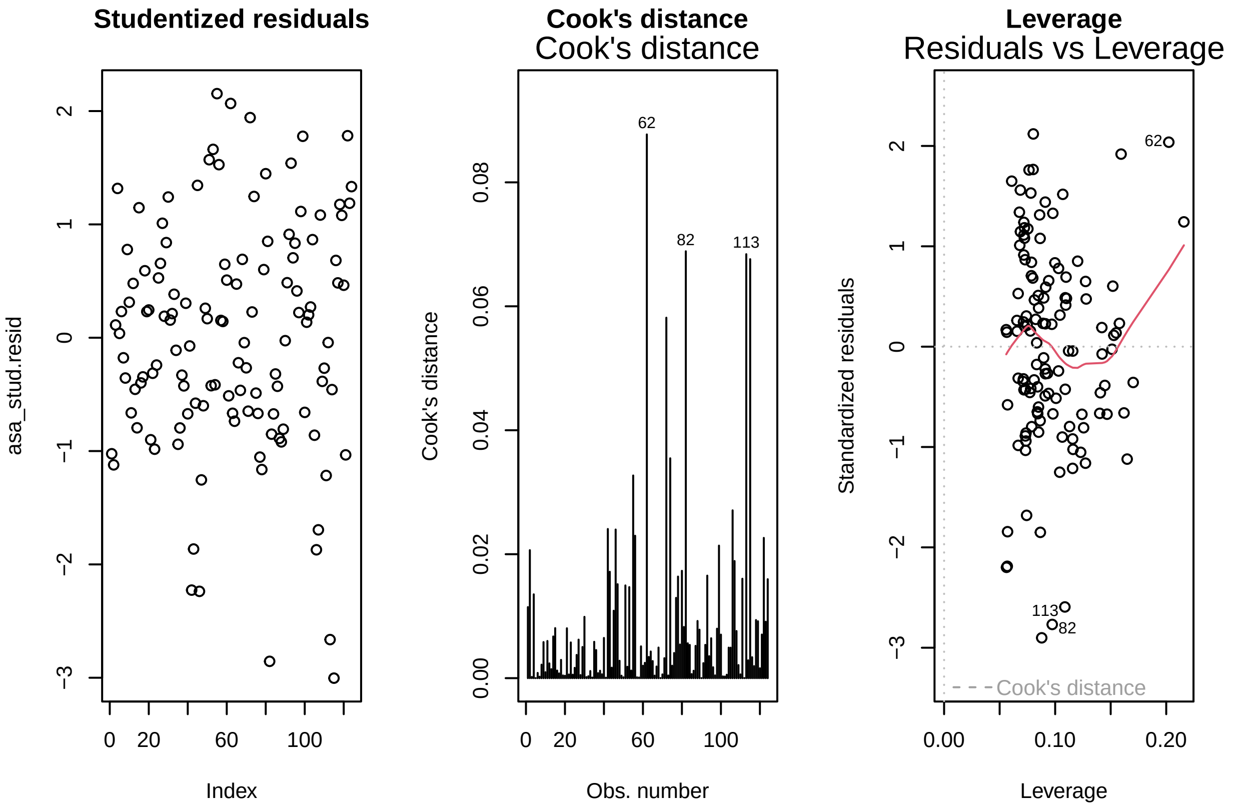

We chose to proceed with as_model17 based on the fact that it had the lowest AIC and best met the assumptions of normality (based on q-q plots and Shapiro-Wilk test statistic), homoscedasticity (based on residuals vs. fitted values, standardized residuals vs. fitted values) and had no significant outliers (studentized residuals between -3 and 3, cook's distances were below 1, and/or leverage was less than 0.5).

Model inference (AS)

In this section, we inspected the values of the coefficients and the corresponding confidence intervals for body mass, treatment (1 = winter, 2 = summer, 3 = MHW), species (1= NL, 2 = AT, 3 = CS), the body mass:species interaction, and treatment:species interaction.

```r
# generate model summary and confidence interval
summary(as_model17)
```

```
##
## Call:
## lm(formula = lnAS ~ lnMass * Species + Treatment * Species, data = Metabolism)
##
## Residuals:
##      Min       1Q   Median       3Q      Max
```

```
## -0.59342 -0.13227  0.01504  0.13554  0.43531
##
## Coefficients:
##                       Estimate Std. Error t value Pr(>|t|)
## (Intercept)           5.777776   0.225368  25.637  < 2e-16 ***
## lnMass                0.070139   0.054219   1.294 0.198457
## Species2              0.805359   0.445662   1.807 0.073430 .
## Species3              0.538155   0.320770   1.678 0.096195 .
## Treatment2            0.203581   0.084480   2.410 0.017590 *
## Treatment3            0.175748   0.086065   2.042 0.043496 *
## lnMass:Species2      -0.366657   0.112561  -3.257 0.001488 **
## lnMass:Species3      -0.173792   0.082253  -2.113 0.036830 *
## Species2:Treatment2   0.431569   0.116225   3.713 0.000321 ***
## Species3:Treatment2   0.274343   0.117923   2.326 0.021793 *
## Species2:Treatment3   0.137034   0.118943   1.152 0.251733
## Species3:Treatment3 -0.005445   0.118152  -0.046 0.963327
## ---
## Signif. codes:  0 '***' 0.001 '**' 0.01 '*' 0.05 '.' 0.1 ' ' 1
##
## Residual standard error: 0.2142 on 112 degrees of freedom
## Multiple R-squared:  0.6915, Adjusted R-squared:  0.6612
## F-statistic: 22.82 on 11 and 112 DF,  p-value: < 2.2e-16
```

```
confint(as_model17)
```

```
##                            2.5 %      97.5 %
## (Intercept)           5.331236885  6.22431440
## lnMass               -0.037289074  0.17756761
## Species2             -0.077662707  1.68838124
## Species3             -0.097410540  1.17372053
## Treatment2            0.036194373  0.37096853
## Treatment3            0.005222296  0.34627420
## lnMass:Species2      -0.589683101 -0.14363080
## lnMass:Species3      -0.336765553 -0.01081913
## Species2:Treatment2   0.201284029  0.66185319
## Species3:Treatment2   0.040693900  0.50799258
## Species2:Treatment3 -0.098635629  0.37270344
## Species3:Treatment3 -0.239547349  0.22865810
```

```
# create a forest plot for model coefficients and confidence intervals
as_forestplot_coef <- data.frame(
  Variable = rownames(summary(as_model17)$coefficients),
  Estimate = summary(as_model17)$coefficients[, 1],
  Lower = confint(as_model17)[, 1],
  Upper = confint(as_model17)[, 2])

ggplot(as_forestplot_coef, aes(x = Estimate, y = Variable)) +
  geom_errorbarh(aes(xmin = Lower, xmax = Upper), height = 0, color = "black", size = 1) +
  geom_vline(xintercept = 0, linetype = "dotted", color = "black") +
  geom_point(size = 3, shape = 21, fill = "white", color = "black") +
  coord_cartesian(xlim = c(-1, 7)) +
  scale_y_discrete(labels = function(x) gsub(":", " - ", x)) +
  theme_classic() +
  xlab("Coefficient Estimate") +
  ylab("") +
```

```r
  ggtitle("AS - Model coefficients")
```

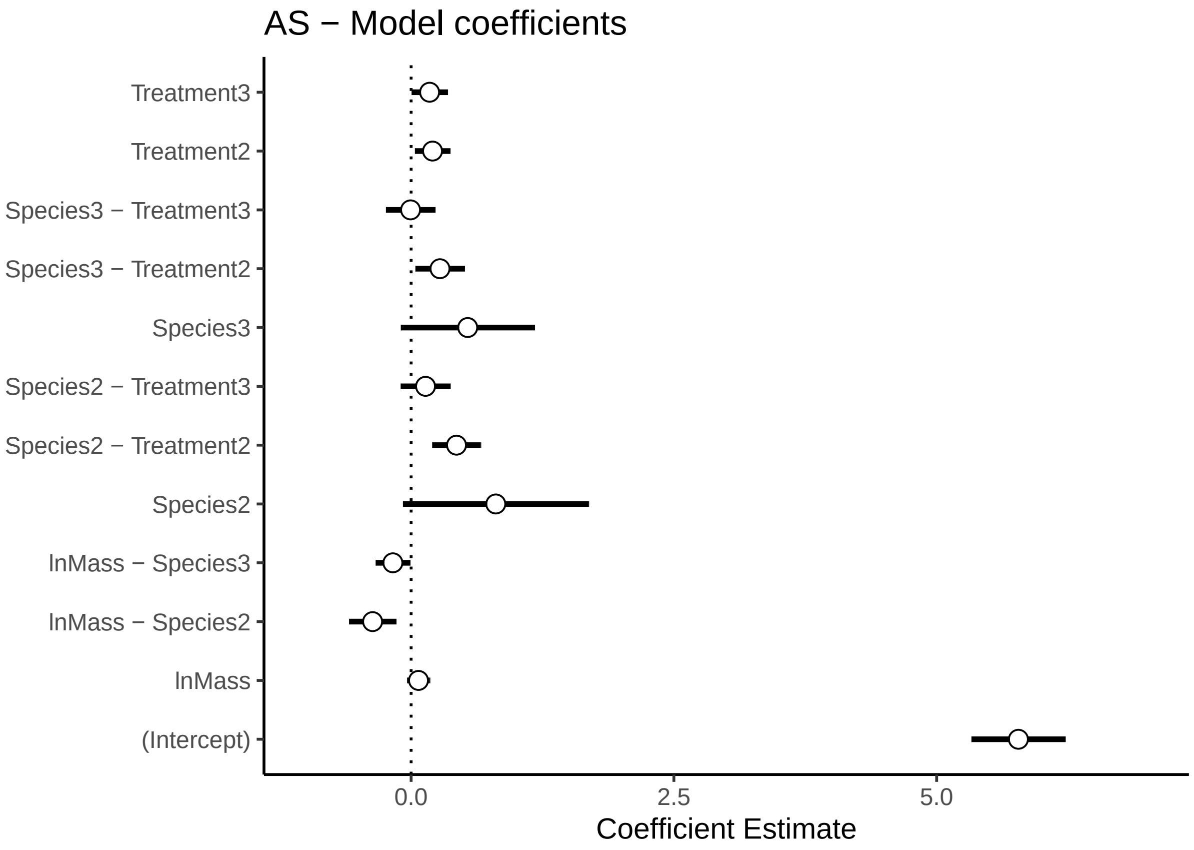


```r
# perform ANOVA
anova(as_model17)
```

```
## Analysis of Variance Table
##
## Response: lnAS
##                     Df Sum Sq Mean Sq F value    Pr(>F)
## lnMass               1 0.8747 0.87473 19.0720 2.822e-05 ***
## Species              2 5.2730 2.63650 57.4842 < 2.2e-16 ***
## Treatment            2 3.9482 1.97412 43.0422 1.357e-14 ***
## lnMass:Species       2 0.6594 0.32970  7.1884  0.001156 **
## Species:Treatment    4 0.7600 0.18999  4.1424  0.003624 **
## Residuals          112 5.1369 0.04586
## ---
## Signif. codes:  0 '***' 0.001 '**' 0.01 '*' 0.05 '.' 0.1 ' ' 1
```

```r
# perform planned contrasts - treatment differences within species
as_NL_emmeans <- emmeans(as_model17, ~ Treatment, at = list(lnMass = 3.9703, Species = "1"))
as_NL_contrasts <- pairs(as_NL_emmeans, adjust = "none")
as_NL_summary <- summary(as_NL_contrasts)
as_NL_p_values <- as_NL_summary$p.value
as_NL_emmeans
```

```
##  Treatment emmean     SE  df lower.CL upper.CL
##  1           6.06 0.0618 112     5.93     6.18
##  2           6.26 0.0572 112     6.15     6.37
```

```
##  3           6.23 0.0595 112     6.11     6.35
##
## Confidence level used: 0.95
```

```
as_AT_emmeans <- emmeans(as_model17, ~ Treatment, at = list(lnMass = 3.9512, Species = "2"))
as_AT_contrasts <- pairs(as_AT_emmeans, adjust = "none")
as_AT_summary <- summary(as_AT_contrasts)
as_AT_p_values <- as_AT_summary$p.value
as_AT_emmeans
```

```
##  Treatment emmean     SE  df lower.CL upper.CL
##  1           5.41 0.0512 112     5.31     5.51
##  2           6.05 0.0623 112     5.92     6.17
##  3           5.72 0.0659 112     5.59     5.85
##
## Confidence level used: 0.95
```

```
as_CS_emmeans <- emmeans(as_model17, ~ Treatment, at = list(lnMass = 3.5553, Species = "3"))
as_CS_contrasts <- pairs(as_CS_emmeans, adjust = "none")
as_CS_summary <- summary(as_CS_contrasts)
as_CS_p_values <- as_CS_summary$p.value
as_CS_emmeans
```

```
##  Treatment emmean     SE  df lower.CL upper.CL
##  1           5.95 0.0572 112     5.83     6.06
##  2           6.43 0.0588 112     6.31     6.54
##  3           6.12 0.0572 112     6.00     6.23
##
## Confidence level used: 0.95
```

```
# perform FDR correction on all contrasts
as_p_values <- c(as_NL_p_values, as_AT_p_values, as_CS_p_values)
as_fdr_corrected_p <- p.adjust(as_p_values, method = "fdr")

as_contrast_names <- c("NL winter - NL summer","NL winter - NL MHW","NL summer - NL MHW",
                       "AT winter - AT summer","AT winter - AT MHW","AT summer - AT MHW",
                       "CS winter - CS summer","CS winter - CS MHW","CS summer - CS MHW")
as_fdr_corrected_p_named <- setNames(as_fdr_corrected_p, as_contrast_names)
as_fdr_corrected_p_named
```

```
## NL winter - NL summer    NL winter - NL MHW    NL summer - NL MHW
##          2.638517e-02          4.893253e-02          7.272464e-01
## AT winter - AT summer    AT winter - AT MHW    AT summer - AT MHW
##          1.403431e-11          6.442525e-04          8.606265e-04
## CS winter - CS summer    CS winter - CS MHW    CS summer - CS MHW
##          2.694619e-07          4.838221e-02          6.442525e-04
```

```
# forest plot of planned contrasts - treatment differences within species
as_create_forestplot <- function(data, plot_title = NULL, show_legend = FALSE)
  {data <- data %>% filter(Treatment %in% c(1, 2, 3))
  data$lower.CL <- data$emmean - 1.96 * data$SE
  data$upper.CL <- data$emmean + 1.96 * data$SE
  data$y_axis <- c(1, 2, 3)
  color_vector <- c("blue", "orange", "red")

  ggplot(data, aes(x = emmean, xmin = lower.CL, xmax = upper.CL, y = y_axis)) +
    geom_point(aes(color = factor(Treatment)), size = 4) +
```

```
    geom_errorbarh(aes(color = factor(Treatment)), height = 0) +
    scale_color_manual(values = color_vector, name = "Treatment", labels =
    c("Winter", "Summer", "MHW")) +
    scale_y_continuous(breaks = c(1, 2, 3), labels = c("Winter", "Summer", "MHW")) +
    labs(title = plot_title, x = "lnAS (mg O2/h/kg)", y = "") +
    theme_classic() +
    theme(legend.position = ifelse(show_legend, "right", "none")) +
    coord_cartesian(xlim = c(5.0, 7.0))}

as_NL_forestplot_emmean <- as_create_forestplot(data.frame(as_NL_emmeans), plot_title="NL")
as_AT_forestplot_emmean <- as_create_forestplot(data.frame(as_AT_emmeans), plot_title="AT")
as_CS_forestplot_emmean <- as_create_forestplot(data.frame(as_CS_emmeans), plot_title="CS")
plot_grid(as_NL_forestplot_emmean, as_AT_forestplot_emmean, as_CS_forestplot_emmean,
  nrow = 1, rel_widths = c(0.33, 0.33, 0.33))
```

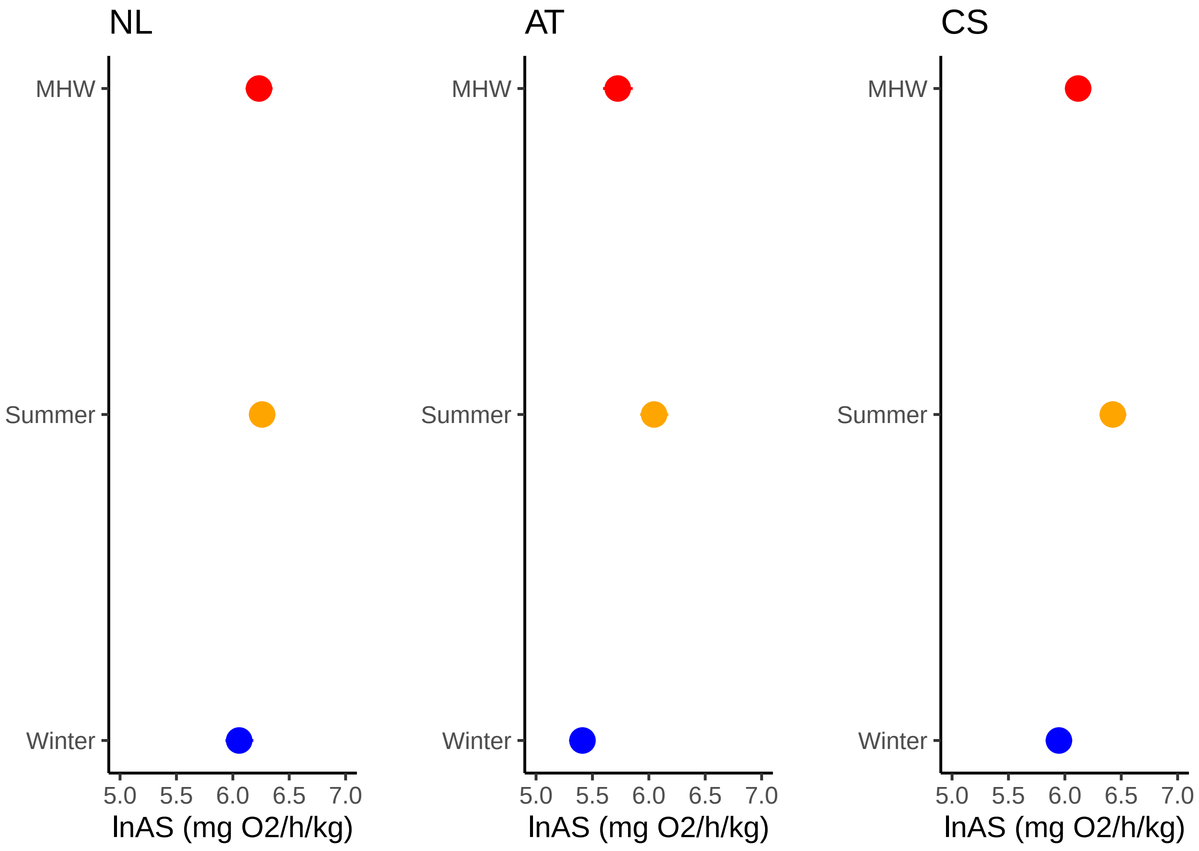

The most parsimonious linear regression model (as_model17) statistically significantly predicted AS ($F_{11,112}$=22.820, p<0.001, adj. R^2=0.661). The interaction between body mass and species ($F_{2,112}$=7.188, p=0.001) and the interaction between species and treatment ($F_{4,112}$=4.142, p=0.004) added significantly to the prediction of AS. With regards to our hypothesis, model coefficients and/or planned contrasts revealed that AS increased from winter to summer in all species, and then from summer to MHW it either plateaued (NL) or decreased (AT and CS).

Model predictions (AS)

In this section, we use the model to predict mean AS with confidence intervals for all species across their respective body mass ranges.

```r
# predicting AS (mean and confidence intervals)
# find the minimum and maximum body mass values for AT and NL in the dataset
nl_min_mass <- 2.6218
nl_max_mass <- 5.1249
at_min_mass <- 3.2794
at_max_mass <- 4.6238
cs_min_mass <- 2.3970
cs_max_mass <- 4.3764

# create a data frame with the desired body mass values for prediction
as_nl_new_predictors <- data.frame(
  Species = '1',
  lnMass = rep(seq(nl_min_mass, nl_max_mass, length.out = 20), times = 3),
  Treatment = factor(rep(c(1, 2, 3), each = 20)))
as_at_new_predictors <- data.frame(
  Species = '2',
  lnMass = rep(seq(at_min_mass, at_max_mass, length.out = 20), times = 3),
  Treatment = factor(rep(c(1, 2, 3), each = 20)))
as_cs_new_predictors <- data.frame(
  Species = '3',
  lnMass = rep(seq(cs_min_mass, cs_max_mass, length.out = 20), times = 3),
  Treatment = factor(rep(c(1, 2, 3), each = 20)))

# use predict() to obtain predictions for each treatment level
as_nl_predictions <- predict(as_model17, newdata = as_nl_new_predictors, interval = "confidence")
as_at_predictions <- predict(as_model17, newdata = as_at_new_predictors, interval = "confidence")
as_cs_predictions <- predict(as_model17, newdata = as_cs_new_predictors, interval = "confidence")

# add the predicted AS values to the data frame
as_nl_new_predictors$lnAS <- as_nl_predictions[,1]
as_nl_new_predictors$lower <- as_nl_predictions[,2]
as_nl_new_predictors$upper <- as_nl_predictions[,3]
as_at_new_predictors$lnAS <- as_at_predictions[,1]
as_at_new_predictors$lower <- as_at_predictions[,2]
as_at_new_predictors$upper <- as_at_predictions[,3]
as_cs_new_predictors$lnAS <- as_cs_predictions[,1]
as_cs_new_predictors$lower <- as_cs_predictions[,2]
as_cs_new_predictors$upper <- as_cs_predictions[,3]

# combine the predictions with the new predictors
as_nl_predicted <- cbind(as_nl_new_predictors, as_nl_predictions)
as_at_predicted <- cbind(as_at_new_predictors, as_at_predictions)
as_cs_predicted <- cbind(as_cs_new_predictors, as_cs_predictions)

# plot the data
as_NL_predictplot <- ggplot() +
  geom_ribbon(data = as_nl_predicted, aes(x = lnMass, y = lnAS, ymin = lwr, ymax = upr,
  fill = Treatment), alpha = 0.2) +
  geom_line(data = as_nl_predicted, aes(x = lnMass, y = lnAS, color = Treatment),
  linewidth = 1) +
  geom_point(data = Metabolism[Metabolism$Species == '1',], aes(x = lnMass, y = lnAS,
  color = Treatment), size = 2) +
  ggtitle("NL") +
```

```
  scale_fill_manual(values = Treatment_colors, guide = "none") +
  scale_color_manual(values = Treatment_colors, labels = Treatment_labels) +
  labs(x = "lnMass (g)", y = "lnAS (mg O2/h/kg)") +
  theme(legend.position = "none") +
  coord_cartesian(xlim = c(2.5, 5.0), ylim = c(5.0, 7.0))

as_AT_predictplot <- ggplot() +
  geom_ribbon(data = as_at_predicted, aes(x = lnMass, y = lnAS, ymin = lwr, ymax = upr,
  fill = Treatment), alpha = 0.2) +
  geom_line(data = as_at_predicted, aes(x = lnMass, y = lnAS, color = Treatment),
  linewidth = 1) +
  geom_point(data = Metabolism[Metabolism$Species == '2',], aes(x = lnMass, y = lnAS,
  color = Treatment), size = 2) +
  ggtitle("AT") +
  scale_fill_manual(values = Treatment_colors, guide = "none") +
  scale_color_manual(values = Treatment_colors, labels = Treatment_labels) +
  labs(x = "lnMass (g)", y = "lnAS (mg O2/h/kg)") +
  theme(legend.position = "none") +
  coord_cartesian(xlim = c(2.5, 5.0), ylim = c(5.0, 7.0))

as_CS_predictplot <- ggplot() +
  geom_ribbon(data = as_cs_predicted, aes(x = lnMass, y = lnAS, ymin = lwr, ymax = upr,
  fill = Treatment), alpha = 0.2) +
  geom_line(data = as_cs_predicted, aes(x = lnMass, y = lnAS, color = Treatment),
  linewidth = 1) +
  geom_point(data = Metabolism[Metabolism$Species == '3',], aes(x = lnMass, y = lnAS,
  color = Treatment), size = 2) +
  ggtitle("CS") +
  scale_fill_manual(values = Treatment_colors, guide = "none") +
  scale_color_manual(values = Treatment_colors, labels = Treatment_labels) +
  labs(x = "lnMass (g)", y = "lnAS (mg O2/h/kg)") +
  theme(legend.position = "right") +
  coord_cartesian(xlim = c(2.5, 5.0), ylim = c(5.0, 7.0))

# Arrange plots in a grid
plot_grid(as_NL_predictplot, as_AT_predictplot, as_CS_predictplot,
  nrow = 1, rel_widths = c(0.28, 0.28, 0.44))
```

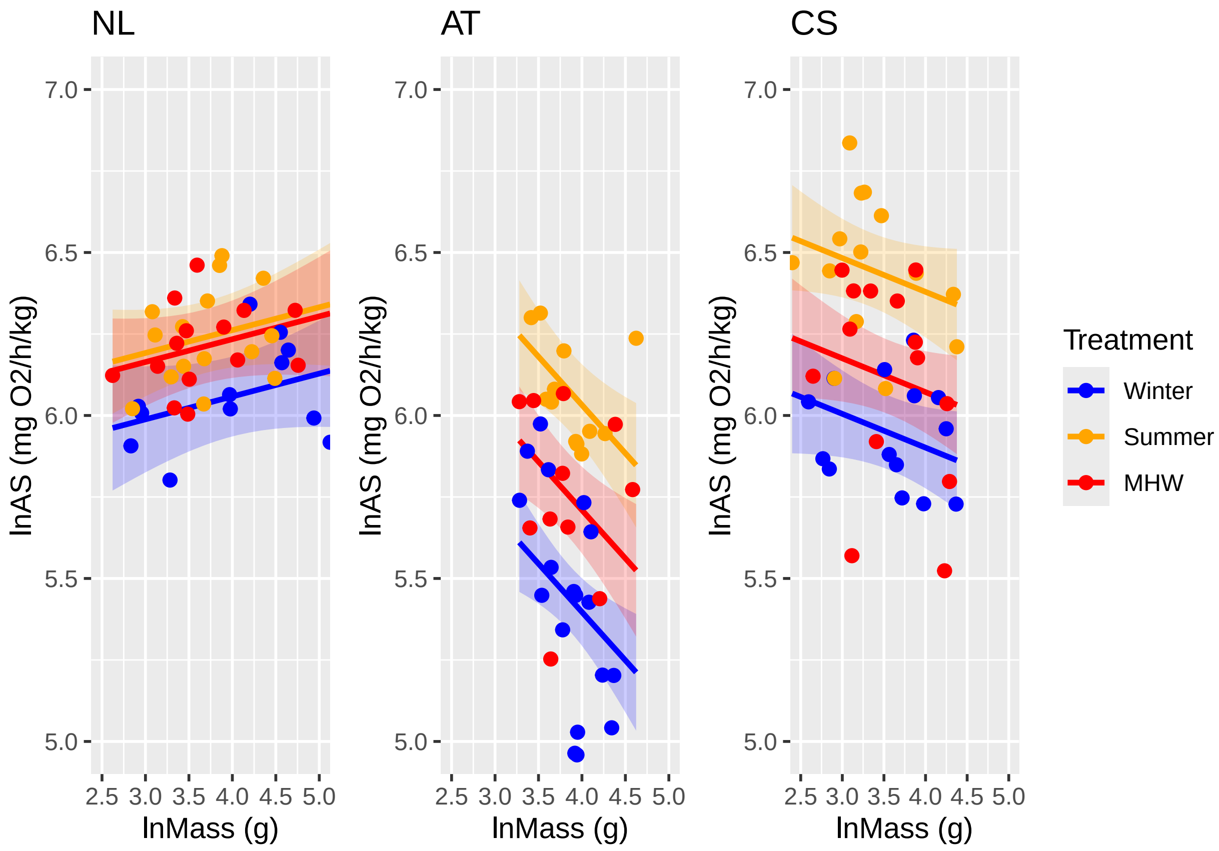
NL
AT
CS
7.0
6.5
6.0
5.5
5.0
2.5 3.0 3.5 4.0 4.5 5.0
lnAS (mg O2/h/kg)
lnMass (g)
Treatment
Winter
Summer
MHW
