# Supplementary material for: Outlasting the Heat: Collapse of Herbivorous Fish Control of Invasive Algae During Marine Heatwaves
Source: Glob Chang Biol. 2025 Aug 20;31(8):e70438. doi: 10.1111/gcb.70438 (PMC12365732; doi:10.1111/gcb.70438)

Supp. Info. 4: Foraging analyses

2025-02-01 - Jeroen Brijs

SETUP

In this section, we loaded the necessary packages and data, and defined useful plotting utilities.

```
# load packages
library(readr) # for loading data file
library(dplyr) # for data manipulation
library(ggplot2) # for plotting
library(ggstance) # for plotting
library(cowplot) # for plotting
library(lmtest) # for assumption testing
library(emmeans) # for posthoc analyses
library(betareg) # for beta regression
library(fitdistrplus) # for beta regression plots
library(effects) # for assumption testing
library(car) # for significance testing

# load data into R studio
Foraging <- read_csv("/Users/xbrije/Documents/Research/20 Herbivores/Stats/Foraging.csv")

# change from a numeric to a factor independent variable and clarify treatment colours
Foraging$Species <- factor(Foraging$Species)
Foraging$Treatment <- factor(Foraging$Treatment)
Treatment_colors <- c("1" = "blue", "2" = "orange", "3" = "red")
Treatment_labels <- c("1" = "Winter", "2" = "Summer", "3" = "MHW")
Species_labels <- c("1" = "NL", "2" = "AT", "3" = "CS")
```

FORAGING RATE (BITE COUNTS)

Data exploration (Foraging rate)

In this section, we explore the relationship between foraging rate and body mass for all species of herbivores.

```
# plot untransformed dependent variable (ForagingRate) vs. continuous independent variable (Mass)
ForagingRate_NL_linearplot <- ggplot(Foraging %>% filter(Species == "1"), aes(x = Mass,
  y = ForagingRate,
  color = Treatment)) +
  geom_point() +
  geom_smooth(method = "lm", se = FALSE, formula = y ~ x) +
  ggtitle("NL") +
  scale_color_manual(values = Treatment_colors, labels = Treatment_labels) +
  labs(x = "Mass (g)", y = "Foraging rate (bites/min)") +
  theme(legend.position = "none") +
  coord_cartesian(xlim = c(0, 150), ylim = c(0, 10))
```

```r
ForagingRate_AT_linearplot <- ggplot(Foraging %>% filter(Species == "2"), aes(x = Mass,
  y = ForagingRate,
  color = Treatment)) +
  geom_point() +
  geom_smooth(method = "lm", se = FALSE, formula = y ~ x) +
  ggtitle("AT") +
  scale_color_manual(values = Treatment_colors, labels = Treatment_labels) +
  labs(x = "Mass (g)", y = "") +
  theme(legend.position = "none") +
  coord_cartesian(xlim = c(0, 150), ylim = c(0, 10))

ForagingRate_CS_linearplot <- ggplot(Foraging %>% filter(Species == "3"), aes(x = Mass,
  y = ForagingRate,
  color = Treatment)) +
  geom_point() +
  geom_smooth(method = "lm", se = FALSE, formula = y ~ x) +
  ggtitle("CS") +
  scale_color_manual(values = Treatment_colors, labels = Treatment_labels) +
  labs(x = "Mass (g)", y = "") +
  theme(legend.position = "none") +
  coord_cartesian(xlim = c(0, 150), ylim = c(0, 10))

legend <- get_legend(
  ggplot(Foraging %>% filter(Species == "1"), aes(x = Mass, y = ForagingRate, color =
    Treatment)) +
    geom_point() +
    scale_color_manual(values = Treatment_colors, labels = Treatment_labels) +
    theme(legend.box.margin = margin(0, 0, 0, 12)))
```

```
## Warning in get_plot_component(plot, "guide-box"): Multiple components found;
## returning the first one. To return all, use `return_all = TRUE`.
```

```r
ForagingRate_plot <- plot_grid(
  plot_grid(ForagingRate_NL_linearplot, ForagingRate_AT_linearplot, ForagingRate_CS_linearplot,
  nrow = 1, rel_widths = c(1, 1, 1)),
  legend,
  ncol = 2,
  rel_widths = c(3, 0.5))

print(ForagingRate_plot)
```

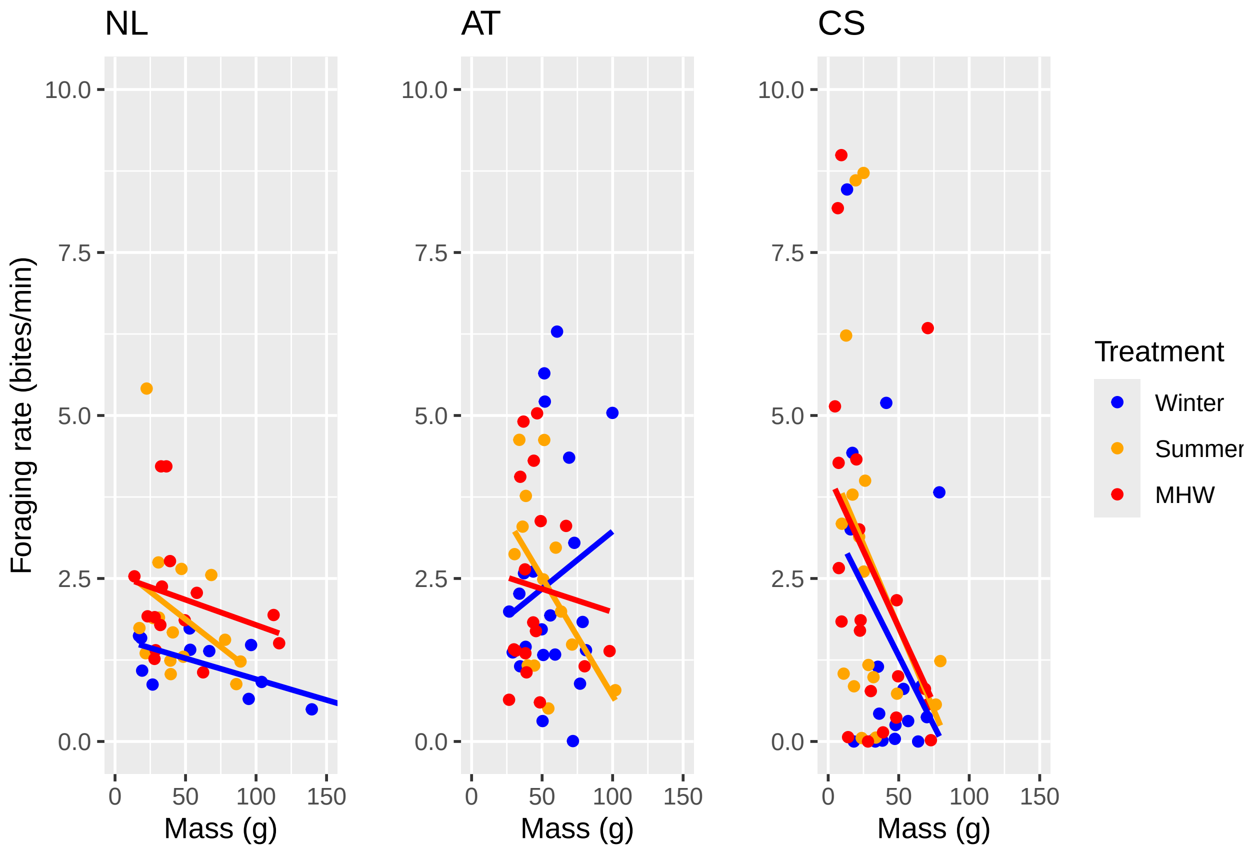


```r
# plot transformed dependent variable (sqForagingRate) vs. continuous independent variable (sqMass)
sqForagingRate_NL_linearplot <- ggplot(Foraging %>% filter(Species == "1"), aes(x = sqMass,
  y = sqForagingRate,
  color = Treatment)) +
  geom_point() +
  geom_smooth(method = "lm", se = FALSE, formula = y ~ x) +
  ggtitle("NL") +
  scale_color_manual(values = Treatment_colors, labels = Treatment_labels) +
  labs(x = "sqMass (g)", y = "sqForaging rate (bites/min)") +
  theme(legend.position = "none") +
  coord_cartesian(xlim = c(0, 15), ylim = c(0, 3))

sqForagingRate_AT_linearplot <- ggplot(Foraging %>% filter(Species == "2"), aes(x = sqMass,
  y = sqForagingRate,
  color = Treatment)) +
  geom_point() +
  geom_smooth(method = "lm", se = FALSE, formula = y ~ x) +
  ggtitle("AT") +
  scale_color_manual(values = Treatment_colors, labels = Treatment_labels) +
  labs(x = "sqMass (g)", y = "") +
  theme(legend.position = "none") +
  coord_cartesian(xlim = c(0, 15), ylim = c(0, 3))

sqForagingRate_CS_linearplot <- ggplot(Foraging %>% filter(Species == "3"), aes(x = sqMass,
  y = sqForagingRate,
  color = Treatment)) +
  geom_point() +
```

```
  geom_smooth(method = "lm", se = FALSE, formula = y ~ x) +
  ggtitle("CS") +
  scale_color_manual(values = Treatment_colors, labels = Treatment_labels) +
  labs(x = "sqMass (g)", y = "") +
  theme(legend.position = "none") +
  coord_cartesian(xlim = c(0, 15), ylim = c(0, 3))

legend <- get_legend(
  ggplot(Foraging %>% filter(Species == "1"), aes(x = sqMass, y = sqForagingRate, color =
    Treatment)) +
    geom_point() +
    scale_color_manual(values = Treatment_colors, labels = Treatment_labels) +
    theme(legend.box.margin = margin(0, 0, 0, 12)))
```

```
## Warning in get_plot_component(plot, "guide-box"): Multiple components found;
## returning the first one. To return all, use `return_all = TRUE`.
```

```
sqForagingRate_plot <- plot_grid(
  plot_grid(sqForagingRate_NL_linearplot, sqForagingRate_AT_linearplot,
  sqForagingRate_CS_linearplot, nrow = 1, rel_widths = c(1, 1, 1)),
  legend,
  ncol = 2,
  rel_widths = c(3, 0.5))

print(sqForagingRate_plot)
```

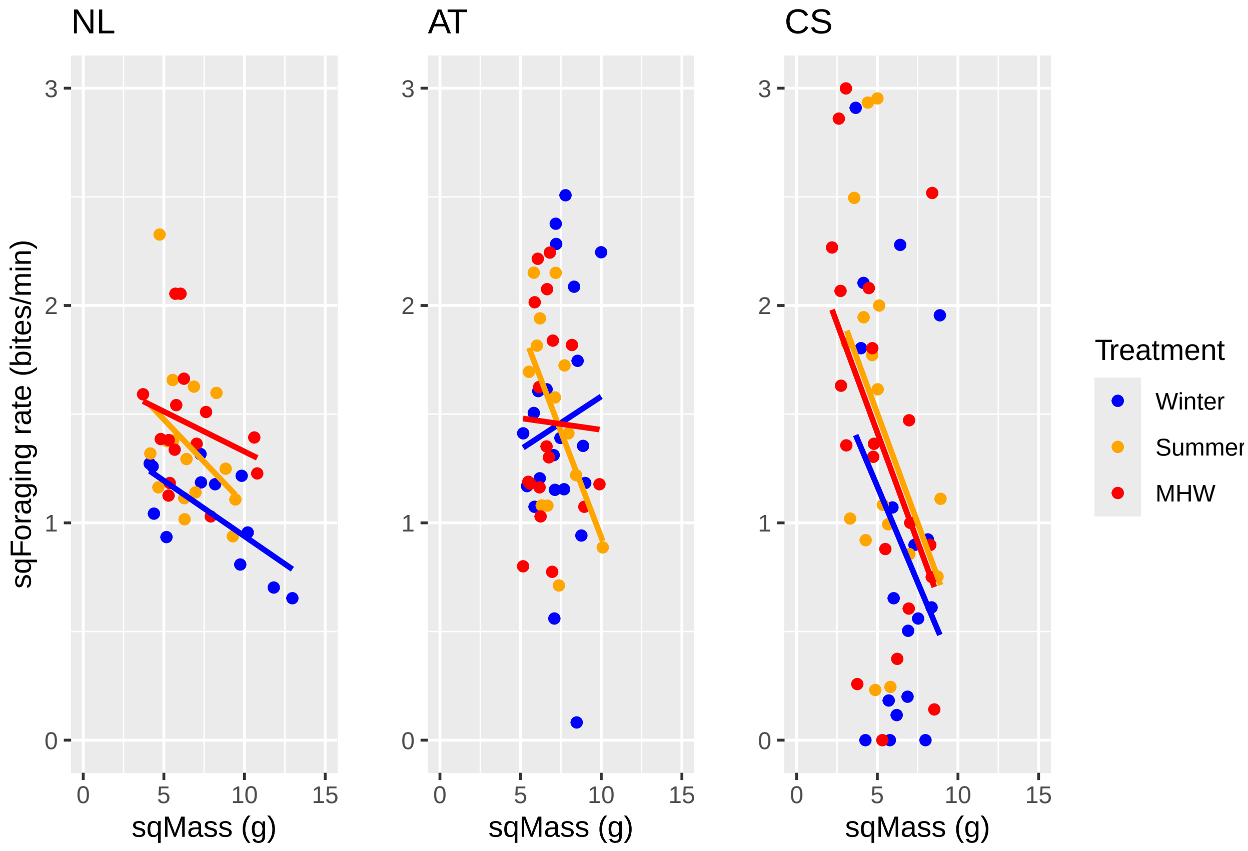

Model fitting (ForagingRate)

In this section, we built a selection of candidate models for ForagingRate. ForagingRate and body mass were log transformed to best meet the assumptions underlying linear regression models.

```
# fit models
ForagingRate_model1 = lm(sqForagingRate ~ 1, data = Foraging)
ForagingRate_model2 = lm(sqForagingRate ~ sqMass, data = Foraging)
ForagingRate_model3 = lm(sqForagingRate ~ Treatment, data = Foraging)
ForagingRate_model4 = lm(sqForagingRate ~ Species, data = Foraging)
ForagingRate_model5 = lm(sqForagingRate ~ sqMass + Treatment, data = Foraging)
ForagingRate_model6 = lm(sqForagingRate ~ sqMass + Species, data = Foraging)
ForagingRate_model7 = lm(sqForagingRate ~ Treatment + Species, data = Foraging)
ForagingRate_model8 = lm(sqForagingRate ~ sqMass * Treatment, data = Foraging)
ForagingRate_model9 = lm(sqForagingRate ~ sqMass * Species, data = Foraging)
ForagingRate_model10 = lm(sqForagingRate ~ Treatment * Species, data = Foraging)
ForagingRate_model11 = lm(sqForagingRate ~ sqMass + Treatment + Species, data = Foraging)
ForagingRate_model12 = lm(sqForagingRate ~ sqMass * Treatment + Species, data = Foraging)
ForagingRate_model13 = lm(sqForagingRate ~ Treatment + sqMass * Species, data = Foraging)
ForagingRate_model14 = lm(sqForagingRate ~ sqMass + Treatment * Species, data = Foraging)
ForagingRate_model15 = lm(sqForagingRate ~ sqMass * Treatment + sqMass * Species, data = Foraging)
ForagingRate_model16 = lm(sqForagingRate ~ sqMass * Treatment + Treatment * Species, data = Foraging)
ForagingRate_model17 = lm(sqForagingRate ~ sqMass * Species + Treatment * Species, data = Foraging)
ForagingRate_model18 = lm(sqForagingRate ~ sqMass * Treatment + sqMass * Species +
  Treatment * Species, data = Foraging)
```

All of the models for ForagingRate were fit without convergence issues.

Model selection (ForagingRate)

In this section, we selected the best-fitting model based on Akaike's Information Criterion (AIC) from the set of candidate models (Burnham and Anderson, 2004).

```
# model selection based on AIC
ForagingRate_aic = AIC(ForagingRate_model1, ForagingRate_model2, ForagingRate_model3,
                       ForagingRate_model4, ForagingRate_model5, ForagingRate_model6,
                       ForagingRate_model7, ForagingRate_model8, ForagingRate_model9,
                       ForagingRate_model10, ForagingRate_model11, ForagingRate_model12,
                       ForagingRate_model13, ForagingRate_model14, ForagingRate_model15,
                       ForagingRate_model16, ForagingRate_model17, ForagingRate_model18)
ForagingRate_aic = ForagingRate_aic[order(ForagingRate_aic$AIC), ]
ForagingRate_aic
```

```
##                      df      AIC
## ForagingRate_model9   7 280.1048
## ForagingRate_model13  9 281.4127
## ForagingRate_model15 11 284.2103
## ForagingRate_model6   5 284.3776
## ForagingRate_model11  7 285.6664
## ForagingRate_model12  9 286.8838
## ForagingRate_model17 13 287.4510
## ForagingRate_model2   3 290.1383
## ForagingRate_model14 11 290.6027
## ForagingRate_model18 15 290.6301
## ForagingRate_model5   5 291.7095
## ForagingRate_model16 13 292.8574
```

```
## ForagingRate_model8   7 294.0652
## ForagingRate_model7   6 297.9414
## ForagingRate_model3   4 298.2988
## ForagingRate_model1   2 299.4137
## ForagingRate_model4   4 300.2244
## ForagingRate_model10 10 302.0852
```

Model checking (ForagingRate)

In this section, we checked the primary assumptions of linear regression models (i.e. normality, homoscedasticity and outliers).

Assumption of normality

```
# check assumption of normality for top four models
# q-q plot
plot(ForagingRate_model13, which = 2)
```

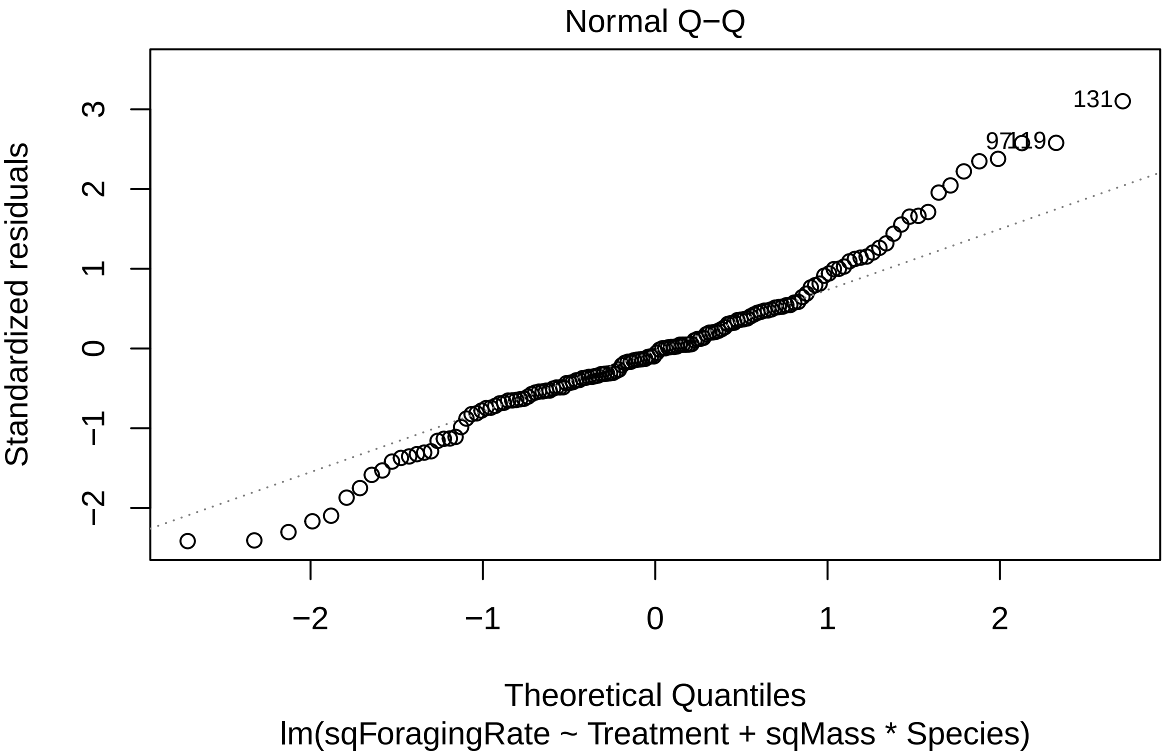


```
# shapiro-wilk test
shapiro.test(resid(ForagingRate_model13))
```

```
##
##  Shapiro-Wilk normality test
##
## data:  resid(ForagingRate_model13)
## W = 0.97666, p-value = 0.01176
```

Assumption of homoscedasticity

```r
# check assumption of homoscedasticity for top four models
# plot residuals vs. fitted, plot standardized residuals vs. fitted
par(mfrow = c(2, 2))
plot(ForagingRate_model13, which = 1, main = "Residuals vs. fitted")
plot(ForagingRate_model13, which = 3, main = "Std. Residuals vs. fitted")
```

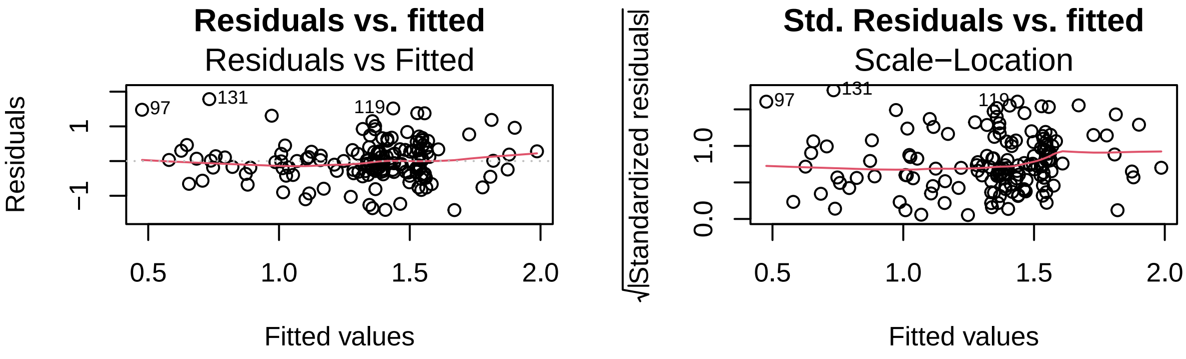

Outliers

```r
# check for outliers in top four models
# plot studentized residuals, cook's distance, and leverage
ForagingRatea_stud.resid <- rstudent(ForagingRate_model13)
par(mfrow = c(1, 3))
plot(ForagingRatea_stud.resid, main = "Studentized residuals")
plot(ForagingRate_model13, which = 4, main = "Cook's distance")
plot(ForagingRate_model13, which = 5, main = "Leverage")
```

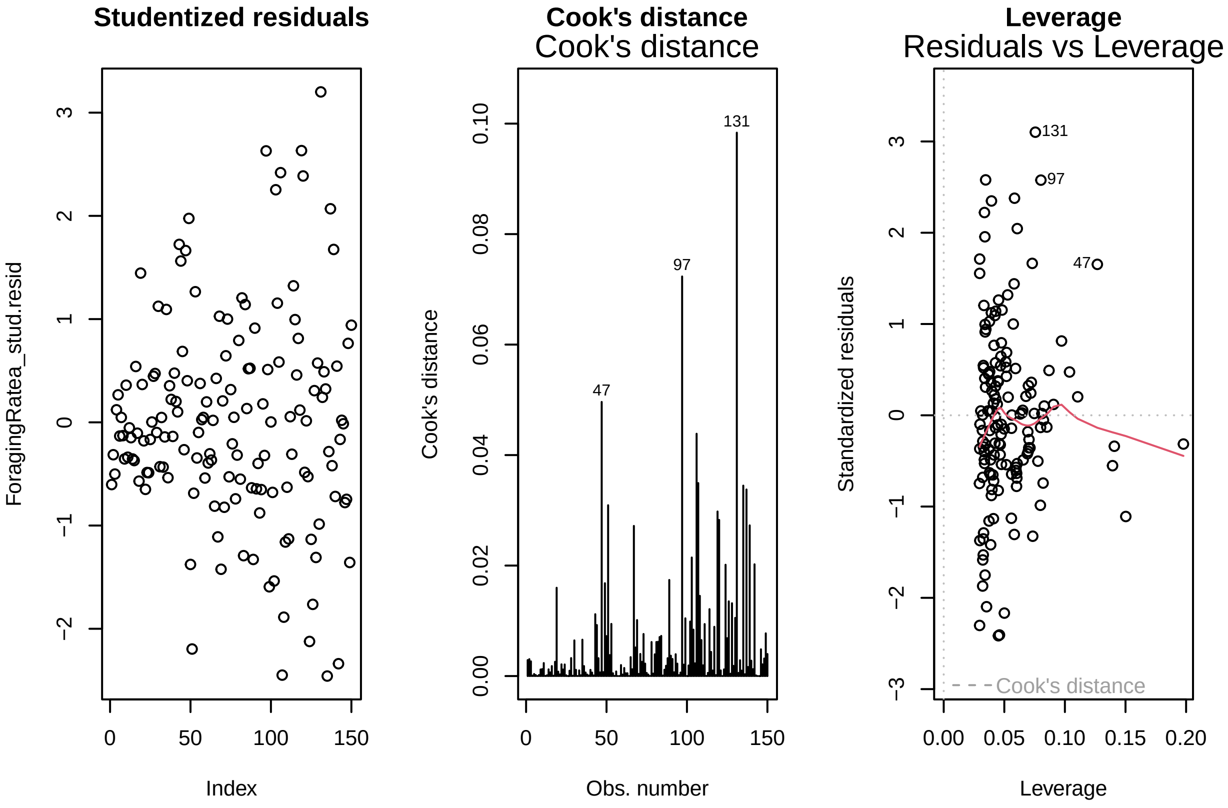

We chose to proceed with ForagingRate_model13 based on the fact that it was the model with the lowest AIC (which included treatment) and best met the assumptions of homoscedasticity (based on residuals vs. fitted values, standardized residuals vs. fitted values). Despite a violation of the normality assumption (based on q-q plots and Shapiro-Wilk test statistic) and the presence of one significant outlier (studentized residual was above 3 but cook's distances were below 1 and leverage was less than 0.5), the model was deemed suitable for analysis due to its robustness to deviations in normality while there was no good biological reason to exclude the outlier.

Model inference (ForagingRate)

In this section, we inspected the values of the coefficients and the corresponding confidence intervals for body mass, treatment (1 = winter, 2 = summer, 3 = MHW), species (1= NL, 2 = AT, 3 = CS), and the body mass:treatment interaction.

```
# generate model summary and confidence interval
summary(ForagingRate_model13)
```

```
##
## Call:
## lm(formula = sqForagingRate ~ Treatment + sqMass * Species, data = Foraging)
##
## Residuals:
##      Min       1Q   Median       3Q      Max
## -1.41246 -0.31318 -0.04433  0.28059  1.78443
##
## Coefficients:
##                  Estimate Std. Error t value Pr(>|t|)
## (Intercept)       1.59155    0.32077   4.962 1.97e-06 ***
## Treatment2        0.17995    0.12518   1.438   0.1528
## Treatment3        0.16199    0.12050   1.344   0.1810
## sqMass           -0.05935    0.04131  -1.436   0.1531
## Species2         -0.10430    0.56284  -0.185   0.8532
## Species3          0.67529    0.39228   1.721   0.0873 .
## sqMass:Species2   0.04265    0.07784   0.548   0.5846
## sqMass:Species3  -0.14235    0.05945  -2.394   0.0180 *
## ---
## Signif. codes:  0 '***' 0.001 '**' 0.01 '*' 0.05 '.' 0.1 ' ' 1
##
## Residual standard error: 0.5984 on 142 degrees of freedom
## Multiple R-squared:  0.1921, Adjusted R-squared:  0.1523
## F-statistic: 4.824 on 7 and 142 DF,  p-value: 6.724e-05
```

```
confint(ForagingRate_model13)
```

```
##                       2.5 %      97.5 %
## (Intercept)      0.95744698  2.22566220
## Treatment2      -0.06750175  0.42740356
## Treatment3      -0.07622024  0.40020453
## sqMass          -0.14101386  0.02232199
## Species2        -1.21692513  1.00831676
## Species3        -0.10017767  1.45075846
## sqMass:Species2 -0.11122453  0.19651703
## sqMass:Species3 -0.25987951 -0.02482166
```

```
# create a forest plot for model coefficients and confidence intervals
ForagingRate_forestplot_coef <- data.frame(
```

```
  Variable = rownames(summary(ForagingRate_model13)$coefficients),
  Estimate = summary(ForagingRate_model13)$coefficients[, 1],
  Lower = confint(ForagingRate_model13)[, 1],
  Upper = confint(ForagingRate_model13)[, 2])

ggplot(ForagingRate_forestplot_coef, aes(x = Estimate, y = Variable)) +
  geom_errorbarh(aes(xmin = Lower, xmax = Upper), height = 0, color = "black") +
  geom_vline(xintercept = 0, linetype = "dotted", color = "black") +
  geom_point(size = 3, shape = 21, fill = "white", color = "black") +
  coord_cartesian(xlim = c(-1, 7)) +
  scale_y_discrete(labels = function(x) gsub(":", " - ", x)) +
  theme_classic() +
  xlab("Coefficient Estimate") +
  ylab("") +
  ggtitle("AS - Model coefficients")
```

```
## Warning: Using the `size` aesthetic with geom_path was deprecated in ggplot2 3.4.0.
## i Please use the `linewidth` aesthetic instead.
## This warning is displayed once every 8 hours.
## Call `lifecycle::last_lifecycle_warnings()` to see where this warning was
## generated.
```

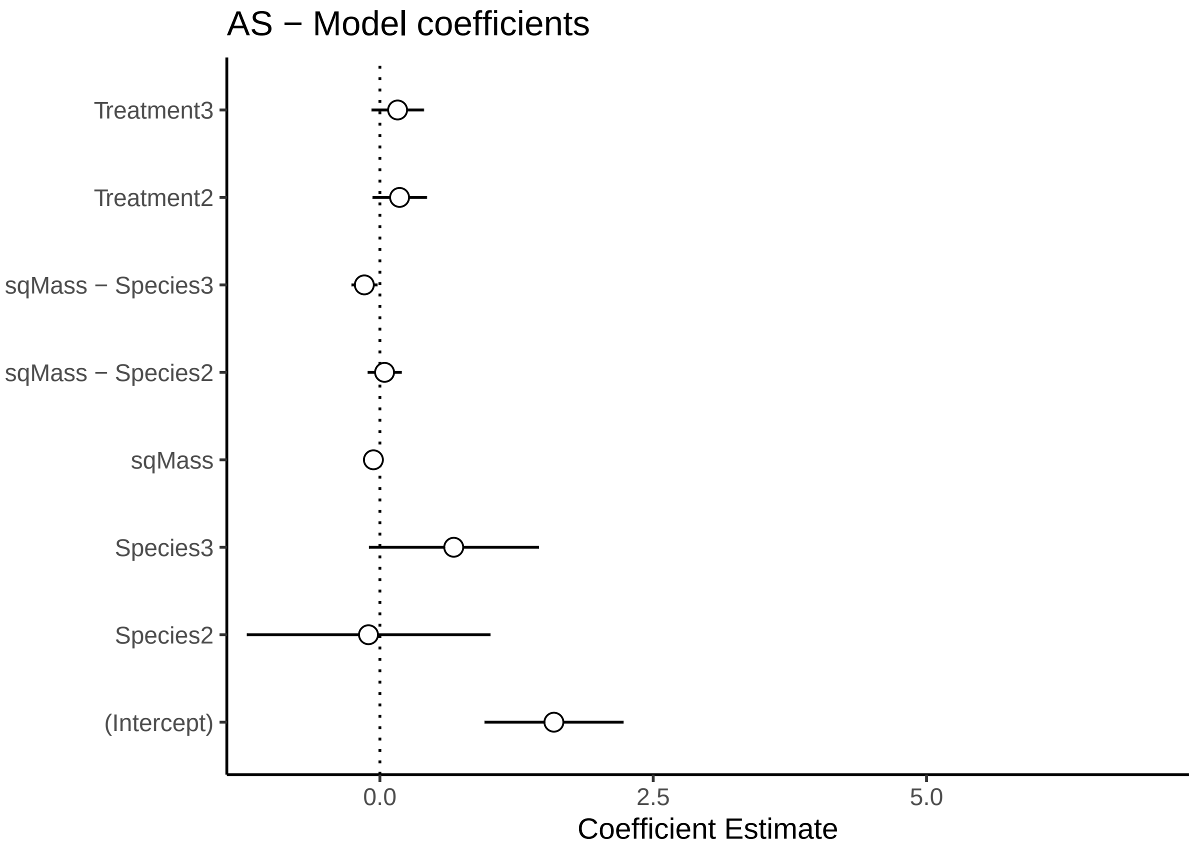


```
# perform ANOVA
anova(ForagingRate_model13)
```

```
## Analysis of Variance Table
##
## Response: sqForagingRate
```

```
##                  Df Sum Sq Mean Sq F value   Pr(>F)
## Treatment         2  2.110  1.0550  2.9463 0.055755 .
## sqMass            1  3.385  3.3854  9.4541 0.002527 **
## Species           2  3.720  1.8601  5.1946 0.006649 **
## sqMass:Species    2  2.876  1.4382  4.0163 0.020105 *
## Residuals       142 50.848  0.3581
## ---
## Signif. codes:  0 '***' 0.001 '**' 0.01 '*' 0.05 '.' 0.1 ' ' 1
```

```
# perform planned contrasts - treatment differences within species
ForagingRate_NL_emmeans <- emmeans(ForagingRate_model13, ~ Treatment, at = list(sqMass = 7.2801,
                                  Species = "1"))
ForagingRate_NL_contrasts <- pairs(ForagingRate_NL_emmeans, adjust = "none")
ForagingRate_NL_summary <- summary(ForagingRate_NL_contrasts)
ForagingRate_NL_p_values <- ForagingRate_NL_summary$p.value
ForagingRate_NL_emmeans
```

```
##  Treatment emmean    SE  df lower.CL upper.CL
##  1           1.16 0.119 142    0.924     1.40
##  2           1.34 0.117 142    1.109     1.57
##  3           1.32 0.115 142    1.093     1.55
##
## Confidence level used: 0.95
```

```
ForagingRate_AT_emmeans <- emmeans(ForagingRate_model13, ~ Treatment, at = list(sqMass = 7.2111,
                                  Species = "2"))
ForagingRate_AT_contrasts <- pairs(ForagingRate_AT_emmeans, adjust = "none")
ForagingRate_AT_summary <- summary(ForagingRate_AT_contrasts)
ForagingRate_AT_p_values <- ForagingRate_AT_summary$p.value
ForagingRate_AT_emmeans
```

```
##  Treatment emmean    SE  df lower.CL upper.CL
##  1           1.37 0.103 142     1.16     1.57
##  2           1.55 0.116 142     1.32     1.78
##  3           1.53 0.110 142     1.31     1.75
##
## Confidence level used: 0.95
```

```
ForagingRate_CS_emmeans <- emmeans(ForagingRate_model13, ~ Treatment, at = list(Mass = 5.9161,
                                  Species = "3"))
ForagingRate_CS_contrasts <- pairs(ForagingRate_CS_emmeans, adjust = "none")
ForagingRate_CS_summary <- summary(ForagingRate_CS_contrasts)
ForagingRate_CS_p_values <- ForagingRate_CS_summary$p.value
ForagingRate_CS_emmeans
```

```
##  Treatment emmean    SE  df lower.CL upper.CL
##  1          0.957 0.110 142    0.739     1.18
##  2          1.137 0.118 142    0.903     1.37
##  3          1.119 0.112 142    0.898     1.34
##
## Confidence level used: 0.95
```

```
# perform FDR correction on all contrasts
ForagingRate_p_values <- c(ForagingRate_NL_p_values, ForagingRate_AT_p_values,
                           ForagingRate_CS_p_values)
ForagingRate_fdr_corrected_p <- p.adjust(ForagingRate_p_values, method = "fdr")
```

```r
ForagingRate_contrast_names <- c("NL winter - NL summer","NL winter - NL MHW",
                                 "NL summer - NL MHW", "AT winter - AT summer",
                                 "AT winter - AT MHW","AT summer - AT MHW",
                                 "CS winter - CS summer","CS winter - CS MHW",
                                 "CS summer - CS MHW")
ForagingRate_fdr_corrected_p_named <- setNames(ForagingRate_fdr_corrected_p,
                                               ForagingRate_contrast_names)
ForagingRate_fdr_corrected_p_named
```

```
## NL winter - NL summer    NL winter - NL MHW    NL summer - NL MHW
##             0.2714947             0.2714947             0.8828790
## AT winter - AT summer    AT winter - AT MHW    AT summer - AT MHW
##             0.2714947             0.2714947             0.8828790
## CS winter - CS summer    CS winter - CS MHW    CS summer - CS MHW
##             0.2714947             0.2714947             0.8828790
```

```r
# forest plot of planned contrasts - treatment differences within species
ForagingRate_create_forestplot <- function(data, plot_title = NULL, show_legend = FALSE) {
  data <- data %>%
    filter(Treatment %in% c(1, 2, 3))
  data$lower.CL <- data$emmean - 1.96 * data$SE
  data$upper.CL <- data$emmean + 1.96 * data$SE
  data$y_axis <- c(1, 2, 3)
  color_vector <- c("blue", "orange", "red")
  ggplot(data, aes(x = emmean, xmin = lower.CL, xmax = upper.CL, y = y_axis)) +
    geom_point(aes(color = factor(Treatment)), size = 4) +
    geom_errorbarh(aes(color = factor(Treatment)), height = 0) +
    scale_color_manual(values = color_vector, name = "Treatment", labels =
                     c("Winter","Summer","MHW")) +
    scale_y_continuous(breaks = c(1, 2, 3), labels = c("Winter", "Summer", "MHW")) +
    labs(title = plot_title, x = "sqForaging rate (bites/min)", y = "") +
    theme_classic() +
    theme(legend.position = ifelse(show_legend, "right", "none")) +
    coord_cartesian(xlim = c(0.5, 2.0))}

ForagingRate_NL_forestplot_emmean <- ForagingRate_create_forestplot(data.frame(ForagingRate_NL_emmeans)
                              plot_title="NL")
ForagingRate_AT_forestplot_emmean <- ForagingRate_create_forestplot(data.frame(ForagingRate_AT_emmeans)
                              plot_title="AT")
ForagingRate_CS_forestplot_emmean <- ForagingRate_create_forestplot(data.frame(ForagingRate_CS_emmeans)
                              plot_title="CS")
plot_grid(ForagingRate_NL_forestplot_emmean, ForagingRate_AT_forestplot_emmean,
          ForagingRate_CS_forestplot_emmean, nrow = 1, rel_widths = c(0.33, 0.33, 0.33))
```

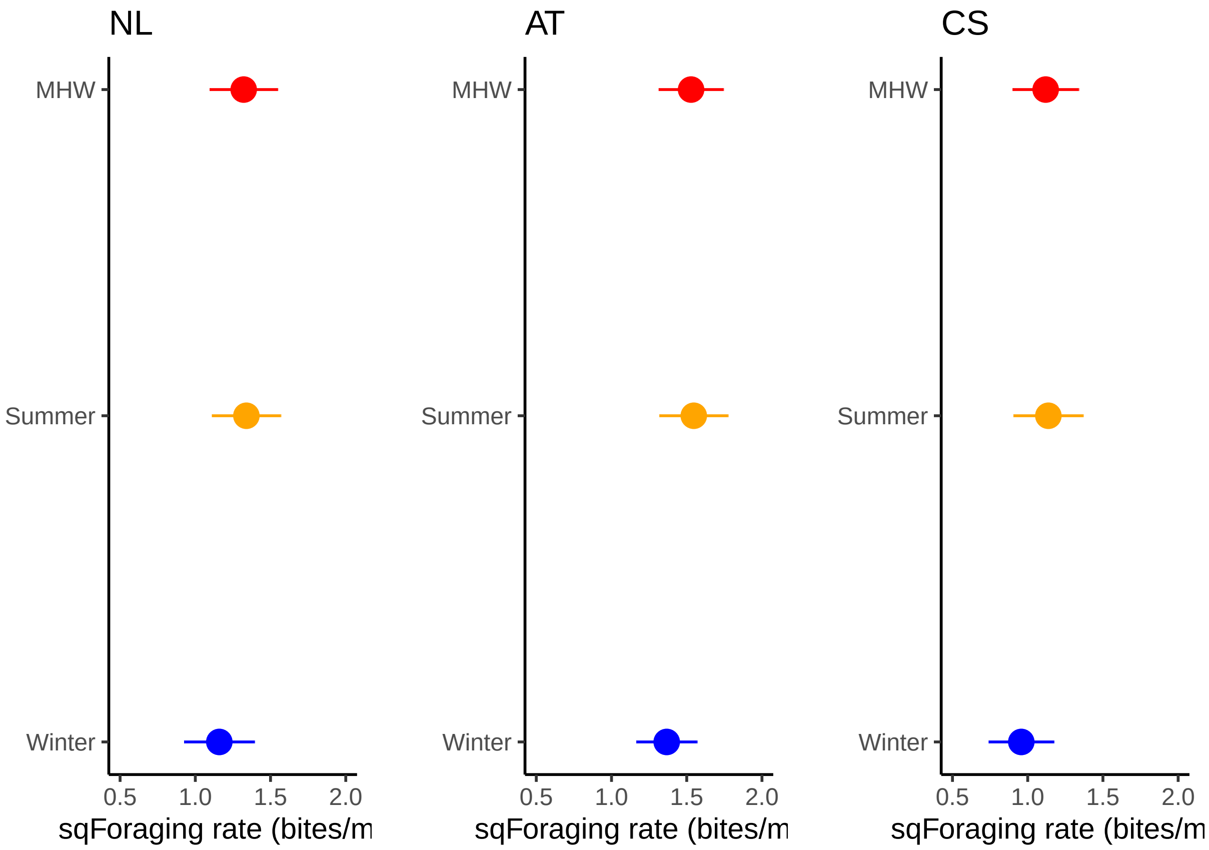

The most parsimonious linear regression model (ForagingRate_model13) statistically significantly predicted foraging rate ($F_{7,142}=4.824$, $p<0.001$, adj. $R2=0.152$). The interaction between body mass and species ($F_{2,142}=4.016$, $p=0.020$) added significantly to the prediction of SMR. With regards to our hypothesis, model coefficients and/or planned contrasts revealed that foraging rate did not change in response to treatment temperature for all species.

Model predictions (ForagingRate)

In this section, we use the model to predict mean foraging rate with confidence intervals for all species across their respective body mass ranges.

```
# predicting ForagingRate (mean and confidence intervals)
# find the minimum and maximum body mass values for AT and NL in the dataset
nl_min_mass <- 3.7094
nl_max_mass <- 12.9673
at_min_mass <- 5.1536
at_max_mass <- 10.0936
cs_min_mass <- 2.1932
cs_max_mass <- 8.9191

# create a data frame with the desired body mass values for prediction
ForagingRate_nl_new_predictors <- data.frame(
  Species = '1',
  sqMass = rep(seq(nl_min_mass, nl_max_mass, length.out = 20), times = 3),
  Treatment = factor(rep(c(1, 2, 3), each = 20)))
ForagingRate_at_new_predictors <- data.frame(
  Species = '2',
  sqMass = rep(seq(at_min_mass, at_max_mass, length.out = 20), times = 3),
```

```
  Treatment = factor(rep(c(1, 2, 3), each = 20)))
ForagingRate_cs_new_predictors <- data.frame(
  Species = '3',
  sqMass = rep(seq(cs_min_mass, cs_max_mass, length.out = 20), times = 3),
  Treatment = factor(rep(c(1, 2, 3), each = 20)))

# use predict() to obtain predictions for each treatment level
ForagingRate_nl_predictions <- predict(ForagingRate_model13, newdata =
                               ForagingRate_nl_new_predictors, interval = "confidence")
ForagingRate_at_predictions <- predict(ForagingRate_model13, newdata =
                               ForagingRate_at_new_predictors, interval = "confidence")
ForagingRate_cs_predictions <- predict(ForagingRate_model13, newdata =
                               ForagingRate_cs_new_predictors, interval = "confidence")

# add the predicted ForagingRate values to the data frame
ForagingRate_nl_new_predictors$sqForagingRate <- ForagingRate_nl_predictions[,1]
ForagingRate_nl_new_predictors$lower <- ForagingRate_nl_predictions[,2]
ForagingRate_nl_new_predictors$upper <- ForagingRate_nl_predictions[,3]
ForagingRate_at_new_predictors$sqForagingRate <- ForagingRate_at_predictions[,1]
ForagingRate_at_new_predictors$lower <- ForagingRate_at_predictions[,2]
ForagingRate_at_new_predictors$upper <- ForagingRate_at_predictions[,3]
ForagingRate_cs_new_predictors$sqForagingRate <- ForagingRate_cs_predictions[,1]
ForagingRate_cs_new_predictors$lower <- ForagingRate_cs_predictions[,2]
ForagingRate_cs_new_predictors$upper <- ForagingRate_cs_predictions[,3]

# combine the predictions with the new predictors
ForagingRate_nl_predicted <- cbind(ForagingRate_nl_new_predictors, ForagingRate_nl_predictions)
ForagingRate_at_predicted <- cbind(ForagingRate_at_new_predictors, ForagingRate_at_predictions)
ForagingRate_cs_predicted <- cbind(ForagingRate_cs_new_predictors, ForagingRate_cs_predictions)

# plot the data
ForagingRate_NL_predictplot <- ggplot() +
  geom_ribbon(data = ForagingRate_nl_predicted, aes(x = sqMass, y = sqForagingRate, ymin = lwr,
  ymax = upr, fill = Treatment), alpha = 0.2) +
  geom_line(data = ForagingRate_nl_predicted, aes(x = sqMass, y = sqForagingRate, color =
  Treatment), linewidth = 1) +
  geom_point(data = Foraging[Foraging$Species == '1',], aes(x = sqMass, y = sqForagingRate,
  color = Treatment), size = 2) +
  ggtitle("NL") +
  scale_fill_manual(values = Treatment_colors, guide = "none") +
  scale_color_manual(values = Treatment_colors, labels = Treatment_labels) +
  labs(x = "sqMass (g)", y = "sqForagingRate (bites/min)") +
  theme(legend.position = "none") +
  coord_cartesian(xlim = c(0, 15), ylim = c(0, 3))

ForagingRate_AT_predictplot <- ggplot() +
  geom_ribbon(data = ForagingRate_at_predicted, aes(x = sqMass, y = sqForagingRate, ymin = lwr,
  ymax = upr, fill = Treatment), alpha = 0.2) +
  geom_line(data = ForagingRate_at_predicted, aes(x = sqMass, y = sqForagingRate, color =
  Treatment), linewidth = 1) +
  geom_point(data = Foraging[Foraging$Species == '2',], aes(x = sqMass, y = sqForagingRate,
  color = Treatment), size = 2) +
  ggtitle("AT") +
```

```
  scale_fill_manual(values = Treatment_colors, guide = "none") +
  scale_color_manual(values = Treatment_colors, labels = Treatment_labels) +
  labs(x = "sqMass (g)", y = "sqForagingRate (bites/min)") +
  theme(legend.position = "none") +
  coord_cartesian(xlim = c(0, 15), ylim = c(0, 3))

ForagingRate_CS_predictplot <- ggplot() +
  geom_ribbon(data = ForagingRate_cs_predicted, aes(x = sqMass, y = sqForagingRate, ymin = lwr,
  ymax = upr, fill = Treatment), alpha = 0.2) +
  geom_line(data = ForagingRate_cs_predicted, aes(x = sqMass, y = sqForagingRate, color =
  Treatment), linewidth = 1) +
  geom_point(data = Foraging[Foraging$Species == '3',], aes(x = sqMass, y = sqForagingRate,
  color = Treatment), size = 2) +
  ggtitle("CS") +
  scale_fill_manual(values = Treatment_colors, guide = "none") +
  scale_color_manual(values = Treatment_colors, labels = Treatment_labels) +
  labs(x = "sqMass (g)", y = "sqForagingRate (bites/min)") +
  theme(legend.position = "right") +
  coord_cartesian(xlim = c(0, 15), ylim = c(0, 3))

plot_grid(ForagingRate_NL_predictplot, ForagingRate_AT_predictplot, ForagingRate_CS_predictplot, nrow =
```

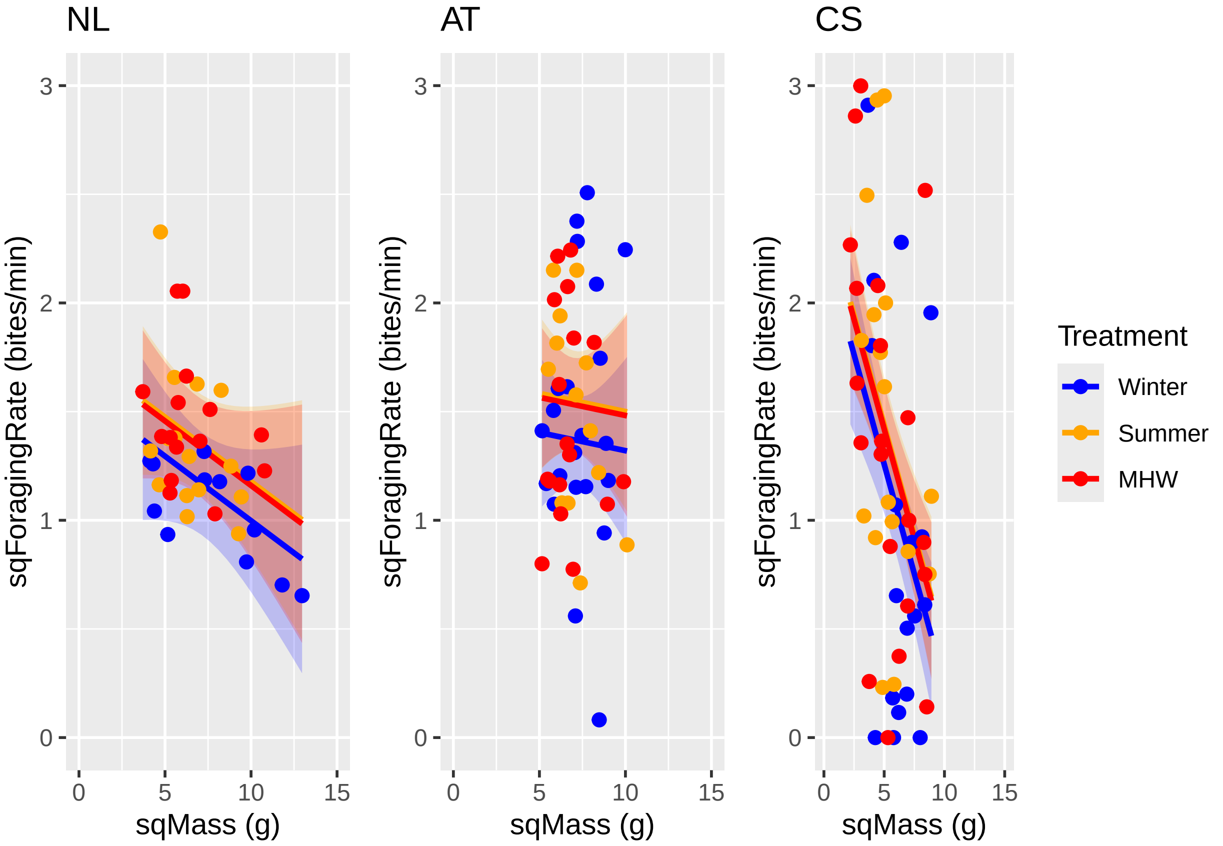

FORAGING ACTIVITY (% time active)

Data exploration (Foraging activity)

In this section, we explore the relationship between foraging activity and body mass for all species of herbivores.

```
# plot untransformed dependent variable (ForagingActivity) vs. continuous independent variable (Mass)
ForagingActivity_NL_linearplot <- ggplot(Foraging %>% filter(Species == "1"), aes(x = Mass,
  y = ForagingActivity,
  color = Treatment)) +
  geom_point() +
  geom_smooth(method = "lm", se = FALSE, formula = y ~ x) +
  ggtitle("NL") +
  scale_color_manual(values = Treatment_colors, labels = Treatment_labels) +
  labs(x = "Mass (g)", y = "ForagingActivity (% time active)") +
  theme(legend.position = "none") +
  coord_cartesian(xlim = c(0, 150), ylim = c(0, 100))

ForagingActivity_AT_linearplot <- ggplot(Foraging %>% filter(Species == "2"), aes(x = Mass,
  y = ForagingActivity,
  color = Treatment)) +
  geom_point() +
  geom_smooth(method = "lm", se = FALSE, formula = y ~ x) +
  ggtitle("AT") +
  scale_color_manual(values = Treatment_colors, labels = Treatment_labels) +
  labs(x = "Mass (g)", y = "") +
  theme(legend.position = "none") +
  coord_cartesian(xlim = c(0, 150), ylim = c(0, 100))

ForagingActivity_CS_linearplot <- ggplot(Foraging %>% filter(Species == "3"), aes(x = Mass,
  y = ForagingActivity,
  color = Treatment)) +
  geom_point() +
  geom_smooth(method = "lm", se = FALSE, formula = y ~ x) +
  ggtitle("CS") +
  scale_color_manual(values = Treatment_colors, labels = Treatment_labels) +
  labs(x = "Mass (g)", y = "") +
  theme(legend.position = "none") +
  coord_cartesian(xlim = c(0, 150), ylim = c(0, 100))

legend <- get_legend(
  ggplot(Foraging %>% filter(Species == "1"), aes(x = Mass, y = ForagingActivity, color =
    Treatment)) +
    geom_point() +
    scale_color_manual(values = Treatment_colors, labels = Treatment_labels) +
    theme(legend.box.margin = margin(0, 0, 0, 12)))
```

```
## Warning in get_plot_component(plot, "guide-box"): Multiple components found;
## returning the first one. To return all, use `return_all = TRUE`.
```

```
ForagingActivity_plot <- plot_grid(
  plot_grid(ForagingActivity_NL_linearplot, ForagingActivity_AT_linearplot,
            ForagingActivity_CS_linearplot, nrow = 1, rel_widths = c(1, 1, 1)),
  legend,
```

```
  ncol = 2,
  rel_widths = c(3, 0.5))

print(ForagingActivity_plot)
```

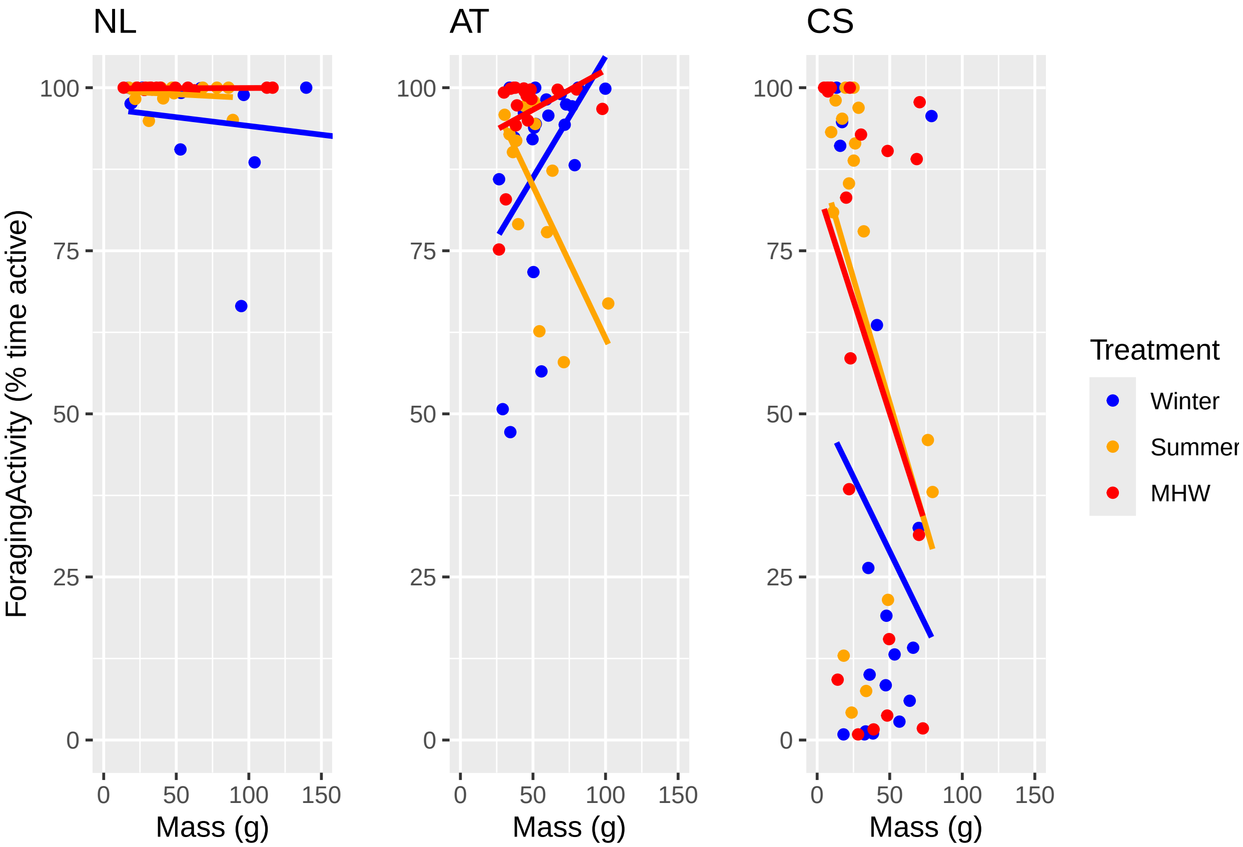


```
# Fit beta distribution to the data
fit_beta <- fitdist(Foraging$transformForagingActivity, "beta",
                    method = "mle",
                    start = list(shape1 = 2, shape2 = 2))

shape1 <- fit_beta$estimate["shape1"]
shape2 <- fit_beta$estimate["shape2"]

ggplot(Foraging, aes(x = transformForagingActivity)) +
  geom_histogram(aes(y = ..density..), bins = 30, color = "black", fill = "lightblue") +
  stat_function(fun = dbeta, args = list(shape1 = shape1, shape2 = shape2),
                color = "red", size = 1) +
  labs(title = "Histogram with Beta Distribution Fit",
       x = "Transformed Foraging Activity (Proportion)",
       y = "Density") +
  theme_classic()
```

```
## Warning: Using `size` aesthetic for lines was deprecated in ggplot2 3.4.0.
## i Please use `linewidth` instead.
## This warning is displayed once every 8 hours.
## Call `lifecycle::last_lifecycle_warnings()` to see where this warning was
## generated.
```

```
## Warning: The dot-dot notation (`..density..`) was deprecated in ggplot2 3.4.0.
## i Please use `after_stat(density)` instead.
## This warning is displayed once every 8 hours.
## Call `lifecycle::last_lifecycle_warnings()` to see where this warning was
## generated.
```

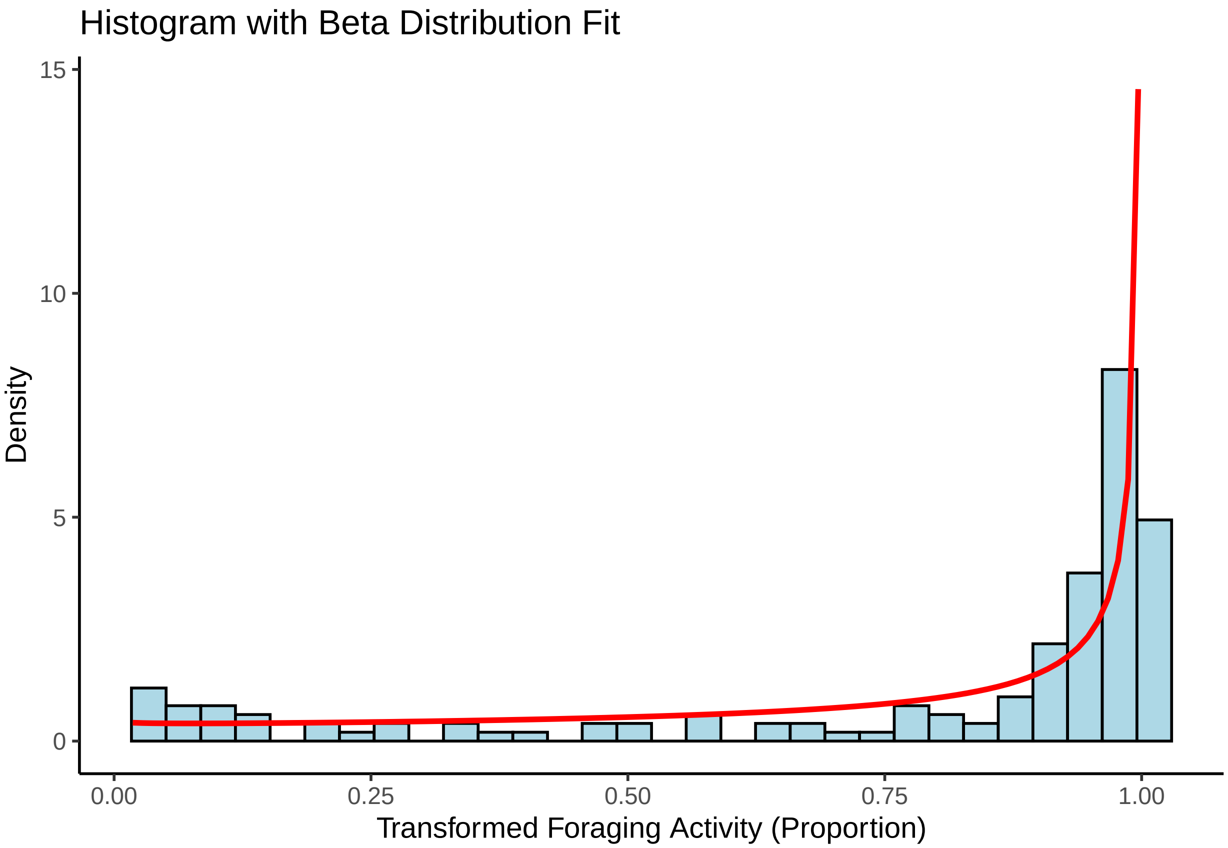

Model fitting (Foraging activity)

In this section, we built a selection of candidate models for foraging activity. Foraging activity and body mass were transformed (adjusted beta transformation and log transformation) to best meet the assumptions of the underlying beta regression models.

```
# fit models
ForagingActivity_model1 = betareg(transformForagingActivity ~ 1, data = Foraging)
ForagingActivity_model2 = betareg(transformForagingActivity ~ lnMass, data = Foraging)
ForagingActivity_model3 = betareg(transformForagingActivity ~ Treatment, data = Foraging)
ForagingActivity_model4 = betareg(transformForagingActivity ~ Species, data = Foraging)
ForagingActivity_model5 = betareg(transformForagingActivity ~ lnMass + Treatment, data =
                          Foraging)
ForagingActivity_model6 = betareg(transformForagingActivity ~ lnMass + Species, data =
                          Foraging)
ForagingActivity_model7 = betareg(transformForagingActivity ~ Treatment + Species, data =
                          Foraging)
ForagingActivity_model8 = betareg(transformForagingActivity ~ lnMass * Treatment, data =
                          Foraging)
ForagingActivity_model9 = betareg(transformForagingActivity ~ lnMass * Species, data = Foraging)
ForagingActivity_model10 = betareg(transformForagingActivity ~ Treatment * Species,
                           data = Foraging)
```

```r
ForagingActivity_model11 = betareg(transformForagingActivity ~ lnMass + Treatment + Species,
                           data = Foraging)
ForagingActivity_model12 = betareg(transformForagingActivity ~ lnMass * Treatment + Species,
                           data = Foraging)
ForagingActivity_model13 = betareg(transformForagingActivity ~ Treatment + lnMass * Species,
                           data = Foraging)
ForagingActivity_model14 = betareg(transformForagingActivity ~ lnMass + Treatment * Species,
                           data = Foraging)
ForagingActivity_model15 = betareg(transformForagingActivity ~ lnMass * Treatment + lnMass *
                           Species, data = Foraging)
ForagingActivity_model16 = betareg(transformForagingActivity ~ lnMass * Treatment + Treatment *
                           Species, data = Foraging)
ForagingActivity_model17 = betareg(transformForagingActivity ~ lnMass * Species + Treatment *
                           Species, data = Foraging)
ForagingActivity_model18 = betareg(transformForagingActivity ~ lnMass * Treatment + lnMass *
                          Species + Treatment * Species, data = Foraging)
```

All of the models for ForagingActivity were fit without convergence issues.

Model selection (Foraging activity)

In this section, we selected the best-fitting model based on Akaike's Information Criterion (AIC) from the set of candidate models (Burnham and Anderson, 2004).

```r
# model selection based on AIC
ForagingActivity_aic = AIC(ForagingActivity_model1, ForagingActivity_model2,
  ForagingActivity_model3, ForagingActivity_model4, ForagingActivity_model5,
  ForagingActivity_model6, ForagingActivity_model7, ForagingActivity_model8,
  ForagingActivity_model9, ForagingActivity_model10, ForagingActivity_model11,
  ForagingActivity_model12, ForagingActivity_model13, ForagingActivity_model14,
  ForagingActivity_model15, ForagingActivity_model16, ForagingActivity_model17,
  ForagingActivity_model18)
ForagingActivity_aic = ForagingActivity_aic[order(ForagingActivity_aic$AIC), ]
ForagingActivity_aic
```

```
##                          df       AIC
## ForagingActivity_model13  9 -327.1983
## ForagingActivity_model17 13 -327.1049
## ForagingActivity_model15 11 -326.2724
## ForagingActivity_model9   7 -325.8032
## ForagingActivity_model18 15 -323.7130
## ForagingActivity_model14 11 -320.1773
## ForagingActivity_model12  9 -318.7020
## ForagingActivity_model11  7 -317.4356
## ForagingActivity_model6   5 -317.3765
## ForagingActivity_model16 13 -317.3378
## ForagingActivity_model10 10 -312.5058
## ForagingActivity_model7   6 -309.3780
## ForagingActivity_model4   4 -305.5981
## ForagingActivity_model1   2 -254.0548
## ForagingActivity_model3   4 -253.7957
## ForagingActivity_model5   5 -252.3453
## ForagingActivity_model2   3 -252.1026
## ForagingActivity_model8   7 -249.0413
```

Model checking (Foraging activity)

In this section, we checked the primary assumptions of linear regression models (i.e. linearity, homoscedasticity and outliers).

Assumption of linearity

```
# plot the Treatment effect
treatment_effect <- Effect("Treatment", ForagingActivity_model13)
plot(treatment_effect, main = "Effect of Treatment on Foraging Activity")
```

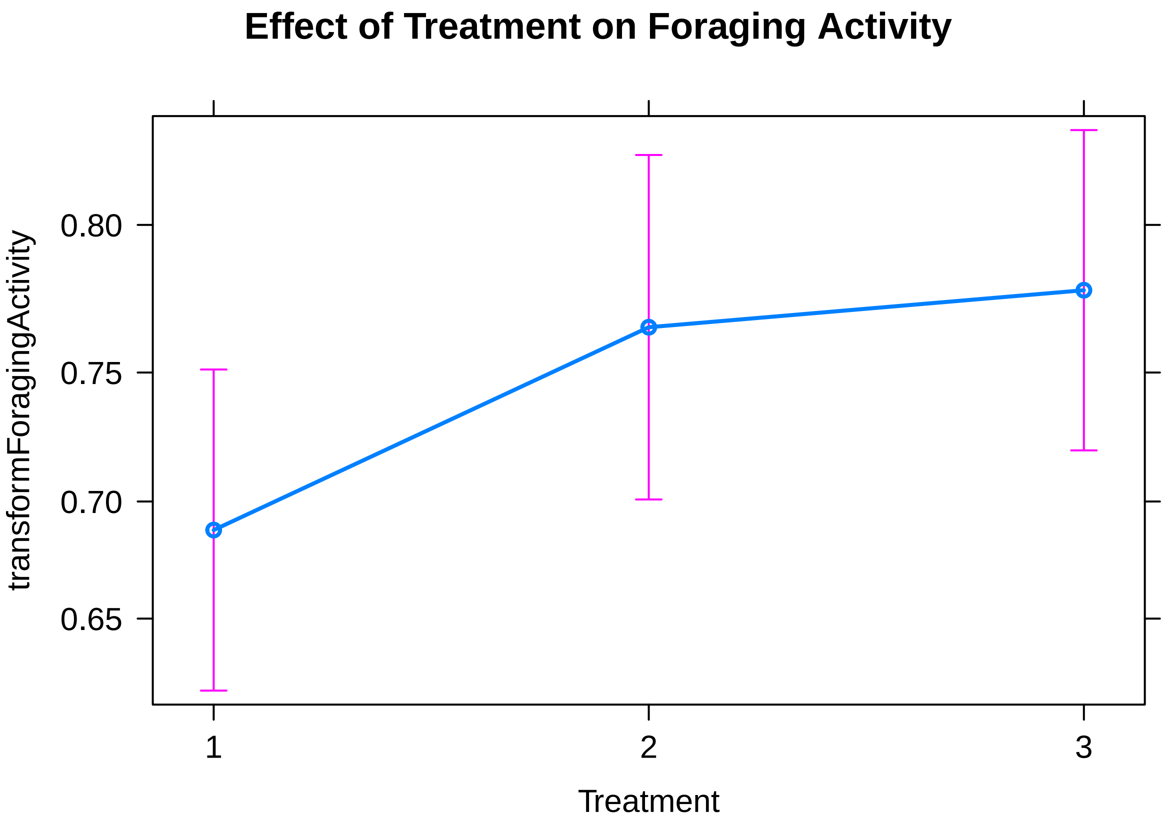


```
# plot the lnMass * Species effect
effect_data <- as.data.frame(Effect(c("lnMass", "Species"), ForagingActivity_model13))

ggplot(effect_data, aes(x = lnMass, y = fit, color = Species)) +
  geom_line() +
  geom_ribbon(aes(ymin = lower, ymax = upper, fill = Species), alpha = 0.2, color = NA) +
  facet_wrap(~ Species, scales = "free_x") + # Facet by Species
  labs(title = "Effect of lnMass by Species",
       x = "lnMass",
       y = "Fitted Value") +
  theme_minimal()
```

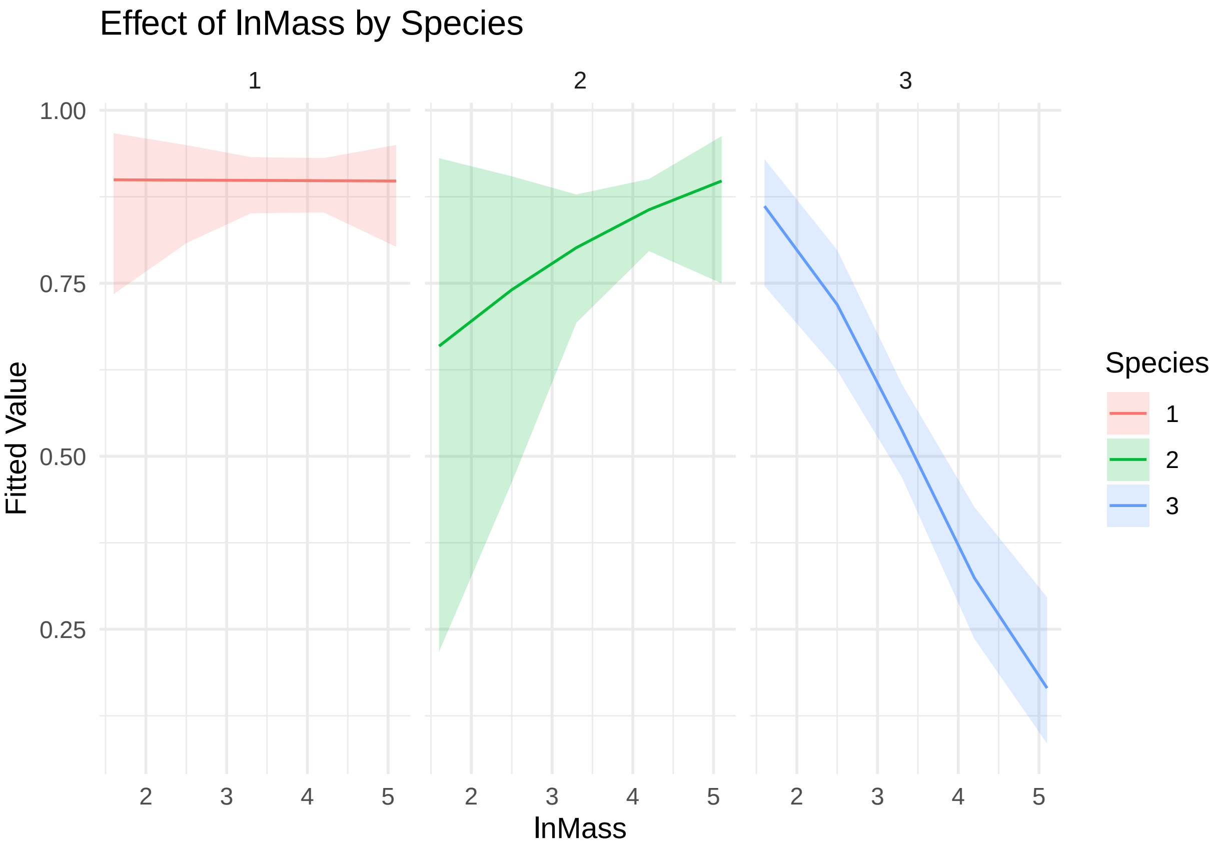

Assumption of homoscedasticity

```
# plot residuals vs fitted values
residuals <- residuals(ForagingActivity_model13, type = "pearson")
fitted <- predict(ForagingActivity_model13, type = "response")

ggplot(data = data.frame(residuals, fitted), aes(x = fitted, y = residuals)) +
  geom_point() +
  geom_smooth(method = "loess", color = "red") +
  labs(title = "Residuals vs Fitted Values",
       x = "Fitted Values",
       y = "Pearson Residuals") +
  theme_minimal()
```

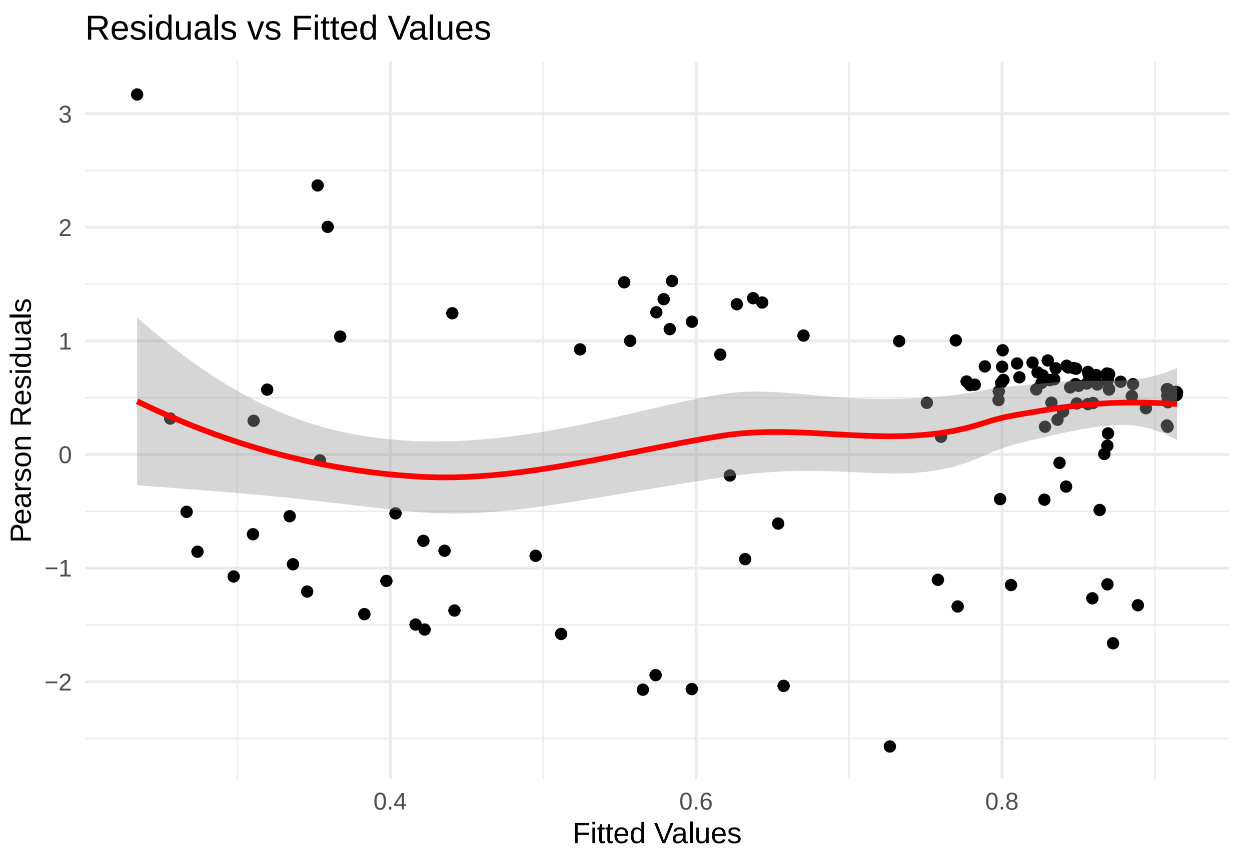


```
# dispersion test
model_constant <- betareg(transformForagingActivity ~ Treatment + lnMass * Species,
                    data = Foraging)
model_variable <- betareg(transformForagingActivity ~ Treatment + lnMass * Species | lnMass,
                    data = Foraging)
lrtest(model_constant, model_variable)
```

```
## Likelihood ratio test
##
## Model 1: transformForagingActivity ~ Treatment + lnMass * Species
## Model 2: transformForagingActivity ~ Treatment + lnMass * Species | lnMass
##   #Df LogLik Df Chisq Pr(>Chisq)
## 1   9  172.6
## 2  10  173.9  1 2.607     0.1064
```

Outliers

```
# plot Cook's distance
cooks <- cooks.distance(ForagingActivity_model13)
threshold <- 1

cooks_data <- data.frame(
  Observation = seq_along(cooks),
  CooksDistance = cooks,
  HighInfluence = cooks > threshold)

ggplot(cooks_data, aes(x = Observation, y = CooksDistance)) +
```

```
  geom_point(aes(color = HighInfluence), size = 2) +
  scale_color_manual(values = c("TRUE" = "red", "FALSE" = "blue"), name = "High Influence") +
  geom_hline(yintercept = threshold, color = "red", linetype = "dashed") +
  labs(title = "Cook's Distance with Threshold at 1",
       x = "Observation Index",
       y = "Cook's Distance") +
  theme_minimal()
```

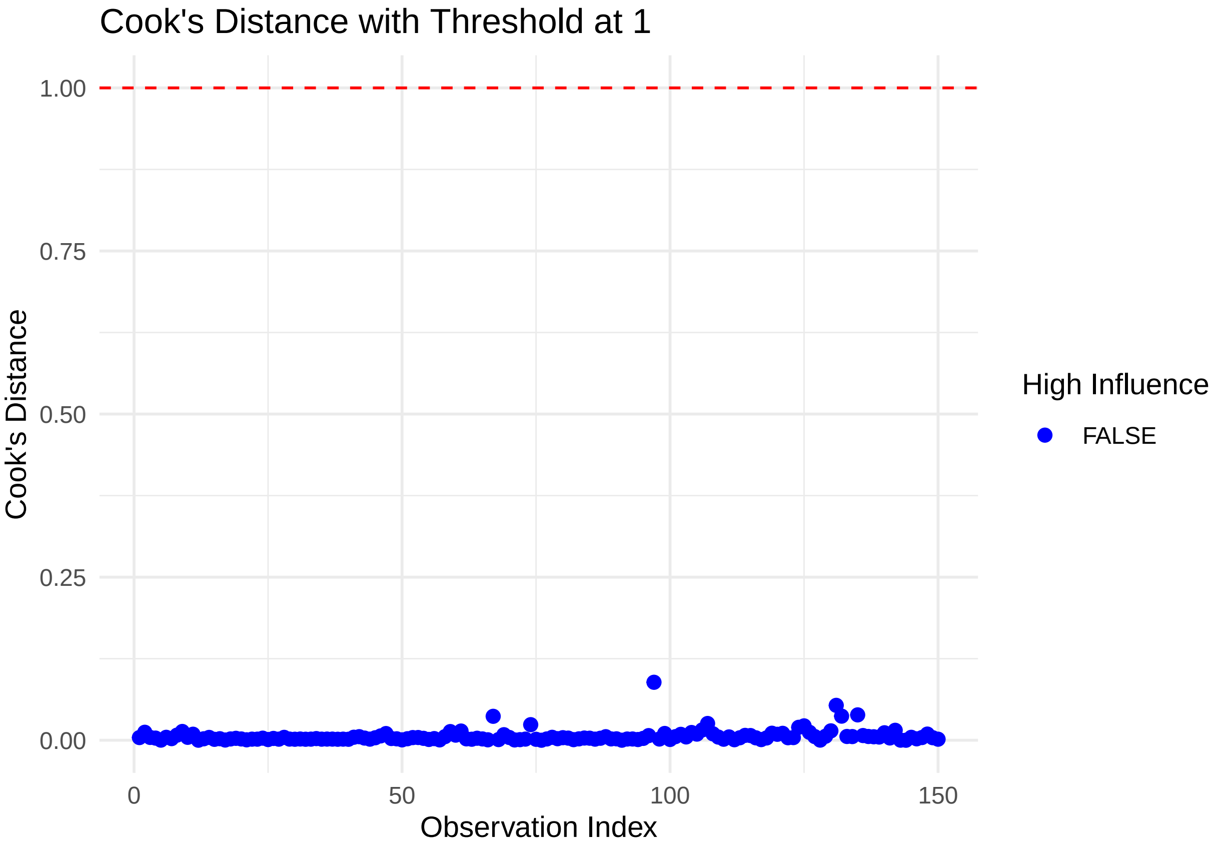


```
# plot leverage values
leverage <- hatvalues(ForagingActivity_model13)

high_leverage <- which(leverage > 0.5)
print(high_leverage)
```

```
## named integer(0)
```

```
leverage_data <- data.frame(
  Observation = seq_along(leverage),
  Leverage = leverage,
  HighLeverage = leverage > 0.5)

ggplot(leverage_data, aes(x = Observation, y = Leverage)) +
  geom_bar(stat = "identity", aes(fill = HighLeverage), color = "black") +
  scale_fill_manual(values = c("FALSE" = "lightblue", "TRUE" = "red"), name = "High Leverage") +
  geom_hline(yintercept = 0.5, color = "red", linetype = "dashed") +
  labs(title = "Leverage Values",
       x = "Observation Index",
       y = "Leverage") +
```

```
theme_minimal()
```

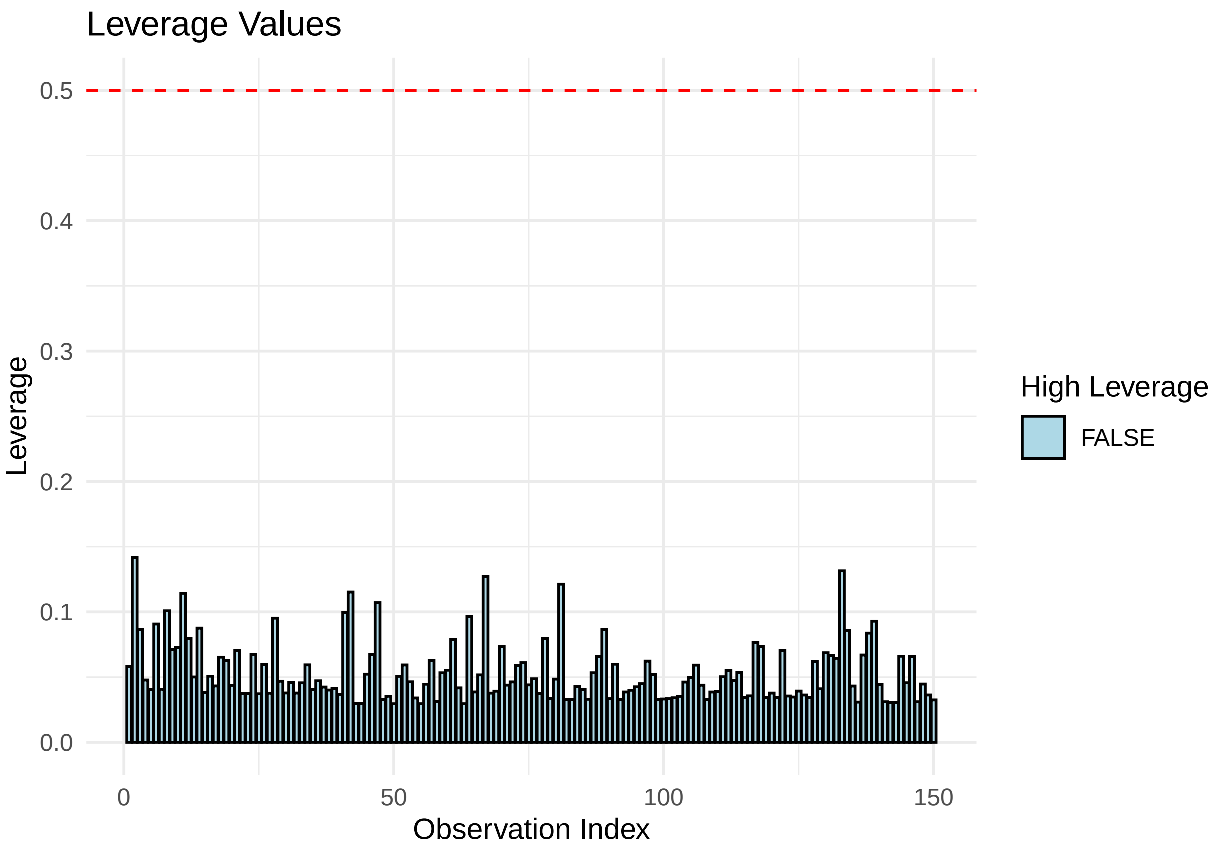

Link functions

```
#Check appropriateness of link function
model_logit <- betareg(transformForagingActivity ~ Treatment + lnMass * Species, link =
               "logit", data = Foraging)
model_probit <- betareg(transformForagingActivity ~ Treatment + lnMass * Species, link =
               "probit", data = Foraging)
model_cloglog <- betareg(transformForagingActivity ~ Treatment + lnMass * Species, link =
               "cloglog", data = Foraging)
AIC(ForagingActivity_model13, model_logit, model_probit, model_cloglog)
```

```
##                          df       AIC
## ForagingActivity_model13  9 -327.1983
## model_logit               9 -327.1983
## model_probit              9 -326.5666
## model_cloglog             9 -325.9002
```

We chose to proceed with ForagingActivity_model13 based on the fact that it had the lowest AIC and best met the assumptions of linearity (based on partial residual plots), homoscedasticity (based on residuals vs. fitted values, comparison between constant and dispersion models) and had no significant outliers (cook's distances were below 1, and/or leverage was less than 0.5), and tested for appropriateness of a link function (not required as AIC was best for original model).

Model inference (Foraging activity)

In this section, we inspected the values of the coefficients and the corresponding confidence intervals for body mass, treatment (1 = winter, 2 = summer, 3 = MHW), species (1= NL, 2 = AT, 3 = CS), and the body mass:species interaction.

```
# generate model summary and confidence interval
summary(ForagingActivity_model13)
```

```
##
## Call:
## betareg(formula = transformForagingActivity ~ Treatment + lnMass * Species,
##     data = Foraging)
##
## Standardized weighted residuals 2:
##     Min      1Q  Median      3Q     Max
## -2.8243 -0.3513  0.0129  0.4412  2.4631
##
## Coefficients (mean model with logit link):
##                  Estimate Std. Error z value Pr(>|z|)
## (Intercept)      1.916679   1.027952   1.865  0.06224 .
## Treatment2       0.395258   0.218958   1.805  0.07105 .
## Treatment3       0.467339   0.212229   2.202  0.02766 *
## lnMass          -0.005484   0.261760  -0.021  0.98328
## Species2        -2.233236   1.937735  -1.152  0.24912
## Species3         1.203323   1.201028   1.002  0.31639
## lnMass:Species2  0.438096   0.499078   0.878  0.38005
## lnMass:Species3 -0.979849   0.329107  -2.977  0.00291 **
##
## Phi coefficients (precision model with identity link):
##       Estimate Std. Error z value Pr(>|z|)
## (phi)   2.5407     0.3019   8.415   <2e-16 ***
## ---
## Signif. codes:  0 '***' 0.001 '**' 0.01 '*' 0.05 '.' 0.1 ' ' 1
##
## Type of estimator: ML (maximum likelihood)
## Log-likelihood: 172.6 on 9 Df
## Pseudo R-squared: 0.5866
## Number of iterations: 32 (BFGS) + 3 (Fisher scoring)
```

```
confint(ForagingActivity_model13)
```

```
##                       2.5 %     97.5 %
## (Intercept)     -0.09806862  3.9314274
## Treatment2      -0.03389309  0.8244083
## Treatment3       0.05137819  0.8833007
## lnMass          -0.51852472  0.5075563
## Species2        -6.03112745  1.5646548
## Species3        -1.15064799  3.5572936
## lnMass:Species2 -0.54007907  1.4162703
## lnMass:Species3 -1.62488714 -0.3348119
## (phi)            1.94896178  3.1325194
```

```
# perform ANOVA
Anova(ForagingActivity_model13, type = "III")
```

```
## Analysis of Deviance Table (Type III tests)
```

```
##
## Response: transformForagingActivity
##                Df   Chisq Pr(>Chisq)
## (Intercept)     1  3.4766  0.0622428 .
## Treatment       2  5.4747  0.0647422 .
## lnMass          1  0.0004  0.9832846
## Species         2  4.0368  0.1328685
## lnMass:Species  2 14.1283  0.0008552 ***
## ---
## Signif. codes:  0 '***' 0.001 '**' 0.01 '*' 0.05 '.' 0.1 ' ' 1
```

```
# perform planned contrasts - treatment differences within species
ForagingActivity_NL_emmeans <- emmeans(ForagingActivity_model13, ~ Treatment, at = list(Mass =
                              3.9703, Species = "1"))
ForagingActivity_NL_contrasts <- pairs(ForagingActivity_NL_emmeans, adjust = "none")
ForagingActivity_NL_summary <- summary(ForagingActivity_NL_contrasts)
ForagingActivity_NL_p_values <- ForagingActivity_NL_summary$p.value
ForagingActivity_NL_emmeans
```

```
##  Treatment emmean     SE  df asymp.LCL asymp.UCL
##  1          0.870 0.0260 Inf     0.819     0.920
##  2          0.908 0.0187 Inf     0.871     0.945
##  3          0.914 0.0176 Inf     0.880     0.948
##
## Confidence level used: 0.95
```

```
ForagingActivity_AT_emmeans <- emmeans(ForagingActivity_model13, ~ Treatment, at = list(lnMass =
                              3.9512, Species = "2"))
ForagingActivity_AT_contrasts <- pairs(ForagingActivity_AT_emmeans, adjust = "none")
ForagingActivity_AT_summary <- summary(ForagingActivity_AT_contrasts)
ForagingActivity_AT_p_values <- ForagingActivity_AT_summary$p.value
ForagingActivity_AT_emmeans
```

```
##  Treatment emmean     SE  df asymp.LCL asymp.UCL
##  1          0.801 0.0306 Inf     0.741     0.861
##  2          0.857 0.0270 Inf     0.804     0.910
##  3          0.865 0.0247 Inf     0.817     0.914
##
## Confidence level used: 0.95
```

```
ForagingActivity_CS_emmeans <- emmeans(ForagingActivity_model13, ~ Treatment, at = list(lnMass =
                              3.5553, Species = "3"))
ForagingActivity_CS_contrasts <- pairs(ForagingActivity_CS_emmeans, adjust = "none")
ForagingActivity_CS_summary <- summary(ForagingActivity_CS_contrasts)
ForagingActivity_CS_p_values <- ForagingActivity_CS_summary$p.value
ForagingActivity_CS_emmeans
```

```
##  Treatment emmean     SE  df asymp.LCL asymp.UCL
##  1          0.405 0.0454 Inf     0.316     0.494
##  2          0.503 0.0494 Inf     0.406     0.600
##  3          0.521 0.0470 Inf     0.429     0.613
##
## Confidence level used: 0.95
```

```
# perform FDR correction on all contrasts
ForagingActivity_p_values <- c(ForagingActivity_NL_p_values, ForagingActivity_AT_p_values,
                               ForagingActivity_CS_p_values)
```

```r
ForagingActivity_fdr_corrected_p <- p.adjust(ForagingActivity_p_values, method = "fdr")

ForagingActivity_contrast_names <- c("NL winter - NL summer","NL winter - NL MHW",
                                     "NL summer - NL MHW", "AT winter - AT summer",
                                     "AT winter - AT MHW","AT summer - AT MHW",
                                     "CS winter - CS summer","CS winter - CS MHW",
                                     "CS summer - CS MHW")
ForagingActivity_fdr_corrected_p_named <- setNames(ForagingActivity_fdr_corrected_p,
                                                    ForagingActivity_contrast_names)
ForagingActivity_fdr_corrected_p_named
```

```
## NL winter - NL summer    NL winter - NL MHW    NL summer - NL MHW
##             0.1282231             0.1215733             0.7362108
## AT winter - AT summer    AT winter - AT MHW    AT summer - AT MHW
##             0.1255346             0.1215733             0.7362108
## CS winter - CS summer    CS winter - CS MHW    CS summer - CS MHW
##             0.1255346             0.1215733             0.7362108
```

```r
# forest plot of planned contrasts - treatment differences within species
ForagingActivity_create_forestplot <- function(data, plot_title = NULL, show_legend = FALSE) {
  data <- data %>%
    filter(Treatment %in% c(1, 2, 3))
  data$lower.CL <- data$emmean - 1.96 * data$SE
  data$upper.CL <- data$emmean + 1.96 * data$SE
  data$y_axis <- c(1, 2, 3)
  color_vector <- c("blue", "orange", "red")
  ggplot(data, aes(x = emmean, xmin = lower.CL, xmax = upper.CL, y = y_axis)) +
    geom_point(aes(color = factor(Treatment)), size = 4) +
    geom_errorbarh(aes(color = factor(Treatment)), height = 0) +
    scale_color_manual(values = color_vector, name = "Treatment", labels = c("Winter","Summer","MHW")) +
    scale_y_continuous(breaks = c(1, 2, 3), labels = c("Winter", "Summer", "MHW")) +
    labs(title = plot_title, x = "transformForaging activity", y = "") +
    theme_classic() +
    theme(legend.position = ifelse(show_legend, "right", "none")) +
    coord_cartesian(xlim = c(0, 1))}

ForagingActivity_NL_forestplot_emmean <- ForagingActivity_create_forestplot(data.frame
                                        (ForagingActivity_NL_emmeans), plot_title="NL")
ForagingActivity_AT_forestplot_emmean <- ForagingActivity_create_forestplot(data.frame
                                        (ForagingActivity_AT_emmeans), plot_title="AT")
ForagingActivity_CS_forestplot_emmean <- ForagingActivity_create_forestplot(data.frame
                                        (ForagingActivity_CS_emmeans), plot_title="CS")
plot_grid(ForagingActivity_NL_forestplot_emmean, ForagingActivity_AT_forestplot_emmean,
          ForagingActivity_CS_forestplot_emmean, nrow = 1, rel_widths = c(0.33, 0.33, 0.33))
```

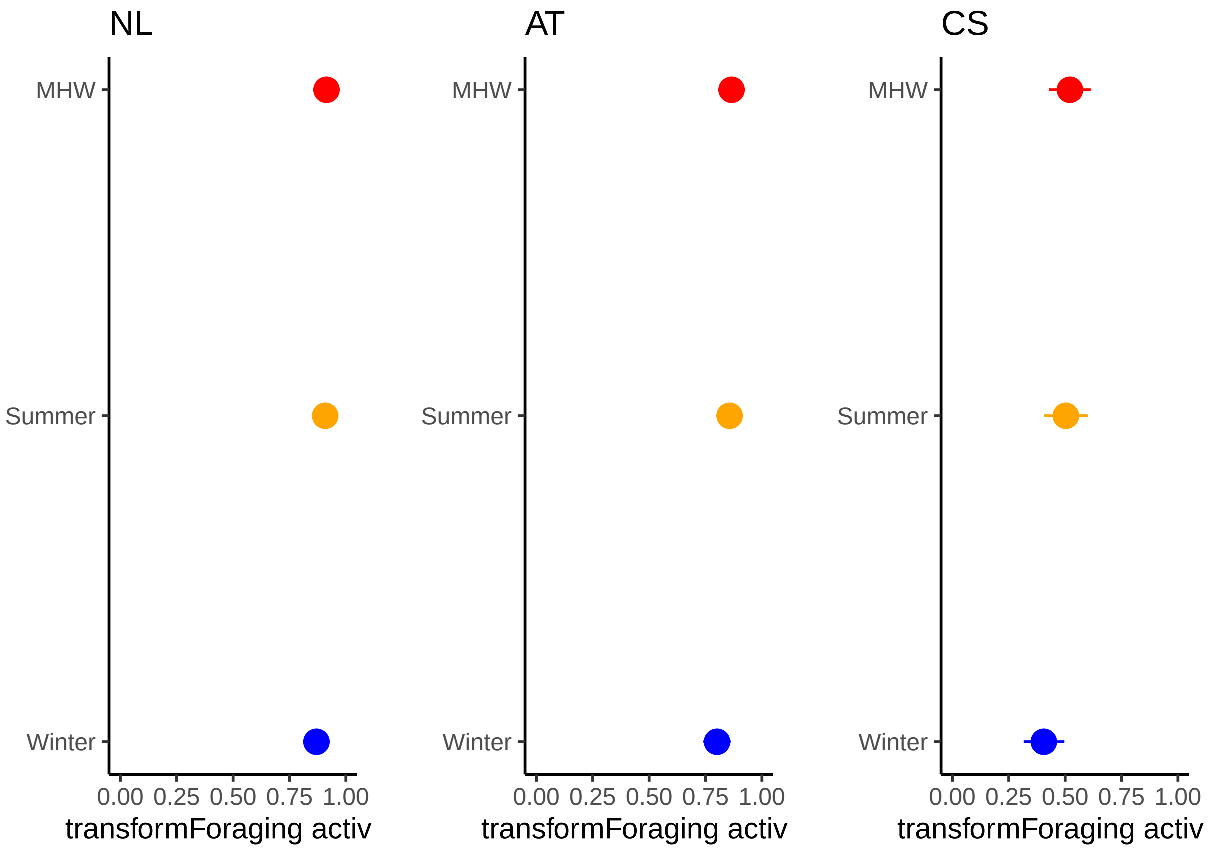

The most parsimonious beta regression model (ForagingActivity_model13) statistically significantly predicted foraging activity (log-likelihood 9 = 172.6, pseudo R2=0.587). The interaction between body mass and species (Chisquare2=14.128, p<0.001) added significantly to the prediction of foraging activity. With regards to our hypothesis, model coefficients and/or planned contrasts revealed that foraging activity did not change in response to treatment temperature for all species.

Model predictions (Foraging activity)

In this section, we use the model to predict mean foraging activity with confidence intervals for all species across their respective body mass ranges.

```
# Predicted response values (mean of the beta distribution)
Foraging$predicted <- predict(ForagingActivity_model13, type = "response")

# Plot with panels for Species and Treatments distinguished within panels
ggplot(Foraging, aes(x = lnMass, y = predicted, color = Treatment)) +
  geom_point() +
  geom_line(aes(linetype = Treatment), size = 1) +
  facet_wrap(~ Species) +
  labs(
    title = "Predicted Foraging Activity",
    x = "Log Mass (lnMass)",
    y = "Predicted Foraging Activity",
    color = "Treatment",
    linetype = "Treatment"
  ) +
  scale_color_manual(values = c("blue", "orange", "red"),
                     labels = c("Winter", "Summer", "MHW")) +
```

```
  theme_minimal()
```

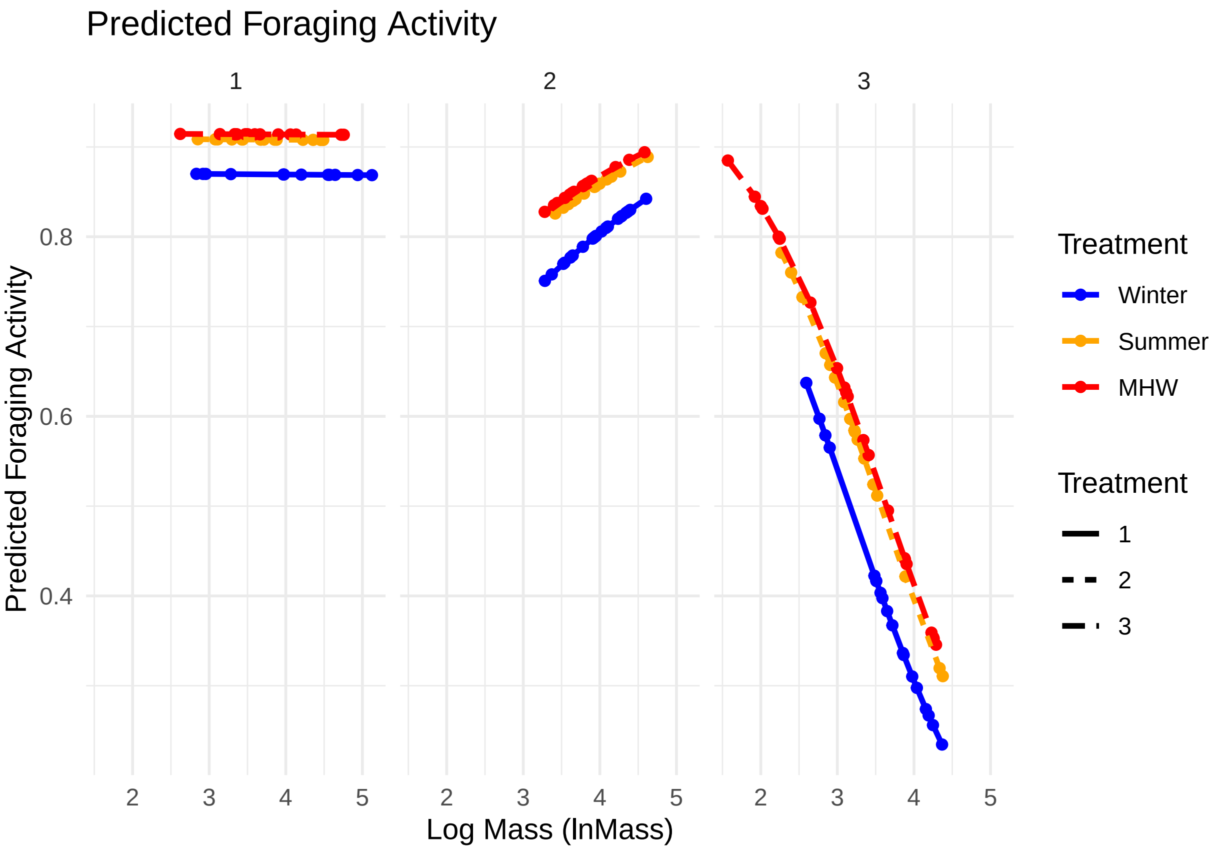

DAILY CHANGES IN BODY MASS (% initial body mass/day)

Data exploration (Percent changes in body mass)

In this section, we explore the relationship between percent changes in body mass and body mass for all species of herbivores.

```
# plot untransformed dependent variable (Percentchange) vs. continuous independent variable (Mass)
Percentchange_NL_linearplot <- ggplot(Foraging %>% filter(Species == "1"), aes(x = Mass,
  y = Percentchange,
  color = Treatment)) +
  geom_point() +
  geom_smooth(method = "lm", se = FALSE, formula = y ~ x) +
  ggtitle("NL") +
  scale_color_manual(values = Treatment_colors, labels = Treatment_labels) +
  labs(x = "Mass (g)", y = "Daily change in body mass (%)") +
  theme(legend.position = "none") +
  coord_cartesian(xlim = c(0, 150), ylim = c(-4.0, 2.0))

Percentchange_AT_linearplot <- ggplot(Foraging %>% filter(Species == "2"), aes(x = Mass,
  y = Percentchange,
  color = Treatment)) +
  geom_point() +
  geom_smooth(method = "lm", se = FALSE, formula = y ~ x) +
```

```r
  ggtitle("AT") +
  scale_color_manual(values = Treatment_colors, labels = Treatment_labels) +
  labs(x = "Mass (g)", y = "") +
  theme(legend.position = "none") +
  coord_cartesian(xlim = c(0, 150), ylim = c(-4.0, 2.0))

Percentchange_CS_linearplot <- ggplot(Foraging %>% filter(Species == "3"), aes(x = Mass,
  y = Percentchange,
  color = Treatment)) +
  geom_point() +
  geom_smooth(method = "lm", se = FALSE, formula = y ~ x) +
  ggtitle("CS") +
  scale_color_manual(values = Treatment_colors, labels = Treatment_labels) +
  labs(x = "Mass (g)", y = "") +
  theme(legend.position = "none") +
  coord_cartesian(xlim = c(0, 150), ylim = c(-4.0, 2.0))

legend <- get_legend(
  ggplot(Foraging %>% filter(Species == "1"), aes(x = Mass, y = Percentchange, color =
    Treatment)) +
    geom_point() +
    scale_color_manual(values = Treatment_colors, labels = Treatment_labels) +
    theme(legend.box.margin = margin(0, 0, 0, 12)))
```

```
## Warning in get_plot_component(plot, "guide-box"): Multiple components found;
## returning the first one. To return all, use `return_all = TRUE`.
```

```r
Percentchange_plot <- plot_grid(
  plot_grid(Percentchange_NL_linearplot, Percentchange_AT_linearplot,
  Percentchange_CS_linearplot, nrow = 1, rel_widths = c(1, 1, 1)),
  legend,
  ncol = 2,
  rel_widths = c(3, 0.5))

print(Percentchange_plot)
```

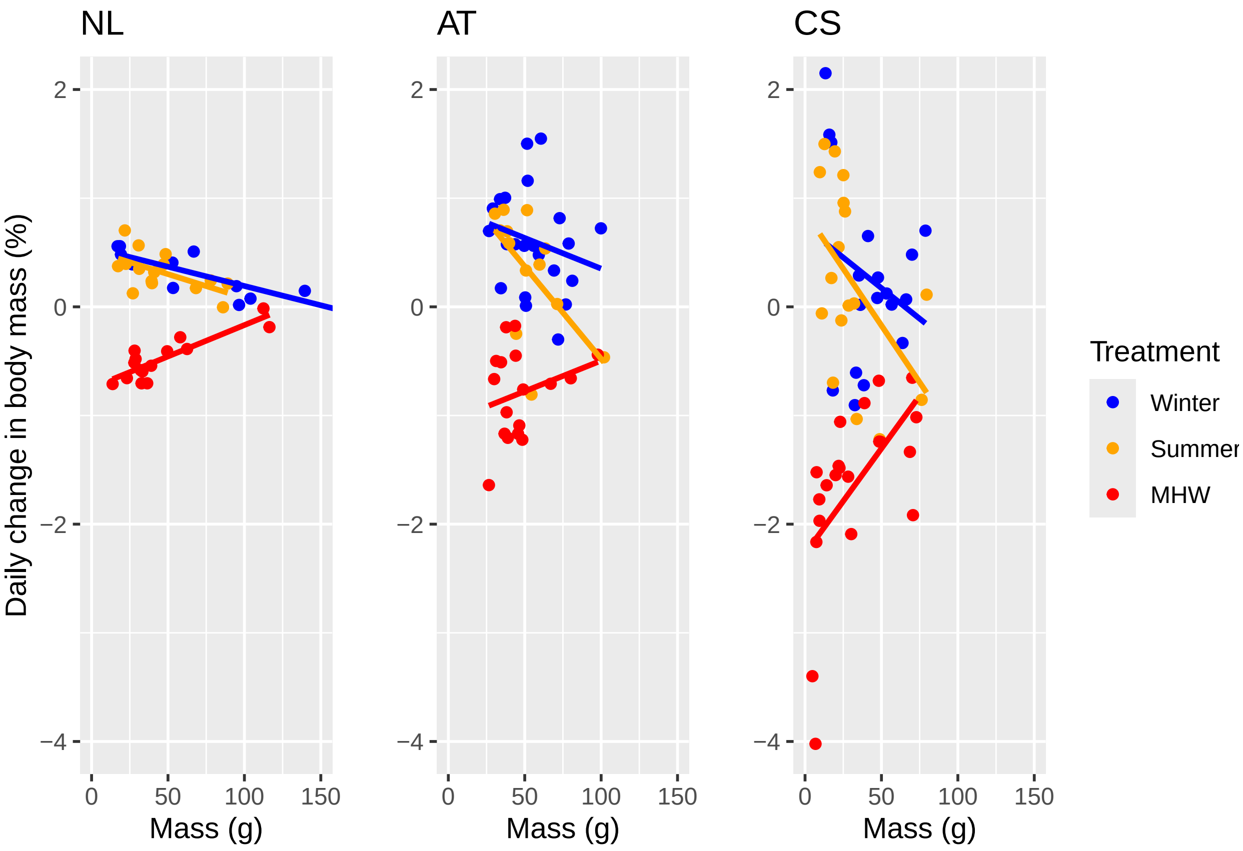

Model fitting (Percentchange)

In this section, we built a selection of candidate models for percent changes in body mass.

```
# fit models
Percentchange_model1 = lm(Percentchange ~ 1, data = Foraging)
Percentchange_model2 = lm(Percentchange ~ Mass, data = Foraging)
Percentchange_model3 = lm(Percentchange ~ Treatment, data = Foraging)
Percentchange_model4 = lm(Percentchange ~ Species, data = Foraging)
Percentchange_model5 = lm(Percentchange ~ Mass + Treatment, data = Foraging)
Percentchange_model6 = lm(Percentchange ~ Mass + Species, data = Foraging)
Percentchange_model7 = lm(Percentchange ~ Treatment + Species, data = Foraging)
Percentchange_model8 = lm(Percentchange ~ Mass * Treatment, data = Foraging)
Percentchange_model9 = lm(Percentchange ~ Mass * Species, data = Foraging)
Percentchange_model10 = lm(Percentchange ~ Treatment * Species, data = Foraging)
Percentchange_model11 = lm(Percentchange ~ Mass + Treatment + Species, data = Foraging)
Percentchange_model12 = lm(Percentchange ~ Mass * Treatment + Species, data = Foraging)
Percentchange_model13 = lm(Percentchange ~ Treatment + Mass * Species, data = Foraging)
Percentchange_model14 = lm(Percentchange ~ Mass + Treatment * Species, data = Foraging)
Percentchange_model15 = lm(Percentchange ~ Mass * Treatment + Mass * Species, data = Foraging)
Percentchange_model16 = lm(Percentchange ~ Mass * Treatment + Treatment * Species,
                        data = Foraging)
Percentchange_model17 = lm(Percentchange ~ Mass * Species + Treatment * Species,
                        data = Foraging)
Percentchange_model18 = lm(Percentchange ~ Mass * Treatment + Mass * Species + Treatment *
                        Species, data = Foraging)
```

All of the models for Percentchange were fit without convergence issues.

Model selection (Percentchange)

In this section, we selected the best-fitting model based on Akaike's Information Criterion (AIC) from the set of candidate models (Burnham and Anderson, 2004).

```
# model selection based on AIC
Percentchange_aic = AIC(Percentchange_model1, Percentchange_model2, Percentchange_model3,
                        Percentchange_model4, Percentchange_model5, Percentchange_model6,
                        Percentchange_model7, Percentchange_model8, Percentchange_model9,
                        Percentchange_model10, Percentchange_model11, Percentchange_model12,
                        Percentchange_model13, Percentchange_model14, Percentchange_model15,
                        Percentchange_model16, Percentchange_model17, Percentchange_model18)
Percentchange_aic = Percentchange_aic[order(Percentchange_aic$AIC), ]
Percentchange_aic
```

```
##                       df      AIC
## Percentchange_model16 13 260.1145
## Percentchange_model12  9 263.1748
## Percentchange_model18 15 263.4350
## Percentchange_model15 11 266.8460
## Percentchange_model10 10 280.0729
## Percentchange_model14 11 281.4120
## Percentchange_model17 13 284.5217
## Percentchange_model8   7 285.4885
## Percentchange_model7   6 292.8943
## Percentchange_model11  7 293.6647
## Percentchange_model13  9 296.5783
## Percentchange_model3   4 307.7570
## Percentchange_model5   5 309.6350
## Percentchange_model4   4 403.9988
## Percentchange_model6   5 405.6542
## Percentchange_model9   7 409.3015
## Percentchange_model2   3 410.6438
## Percentchange_model1   2 411.1127
```

Model checking (Percentchange)

In this section, we checked the primary assumptions of linear regression models (i.e. normality, homoscedasticity and outliers).

Assumption of normality

```
# check assumption of normality for top four models
# q-q plot
plot(Percentchange_model16, which = 2)
```

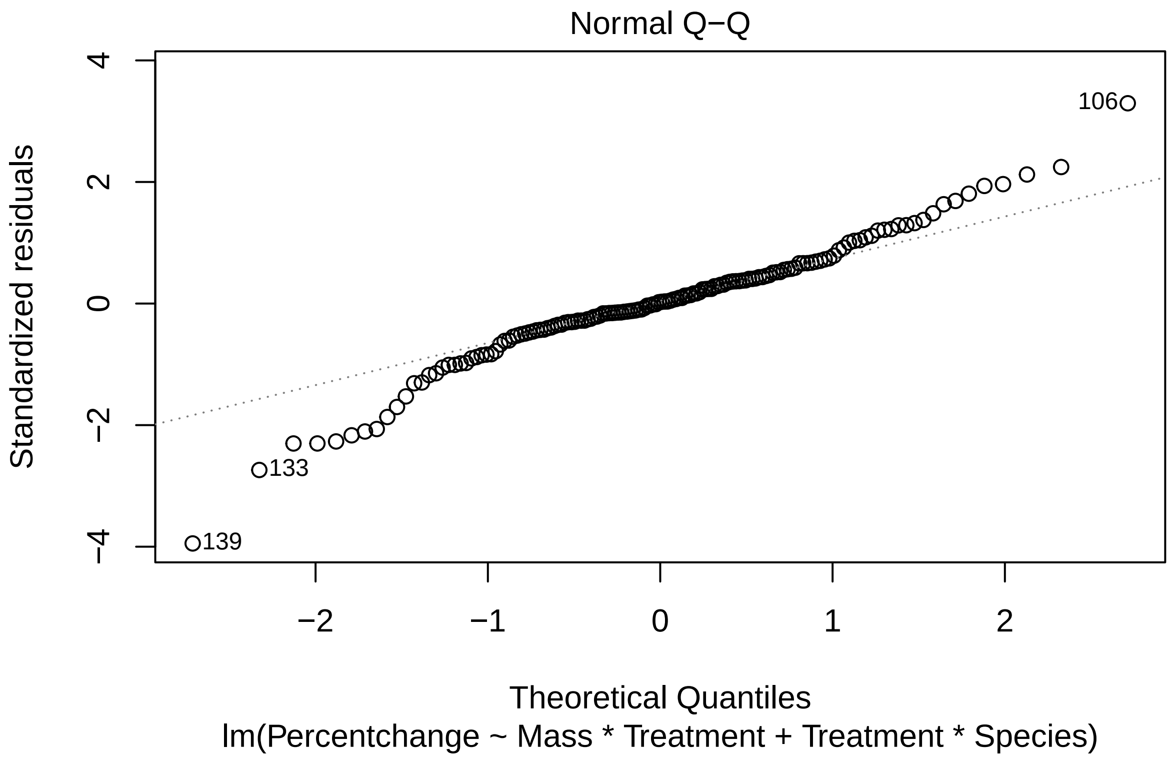


```
# shapiro-wilk test
shapiro.test(resid(Percentchange_model16))
```

```
##
##  Shapiro-Wilk normality test
##
## data:  resid(Percentchange_model16)
## W = 0.9615, p-value = 0.0003375
```

Assumption of homoscedasticity

```
# check assumption of homoscedasticity for top four models
# plot residuals vs. fitted, plot standardized residuals vs. fitted
par(mfrow = c(2, 2))
plot(Percentchange_model16, which = 1, main = "Residuals vs. fitted")
plot(Percentchange_model16, which = 3, main = "Std. Residuals vs. fitted")
```

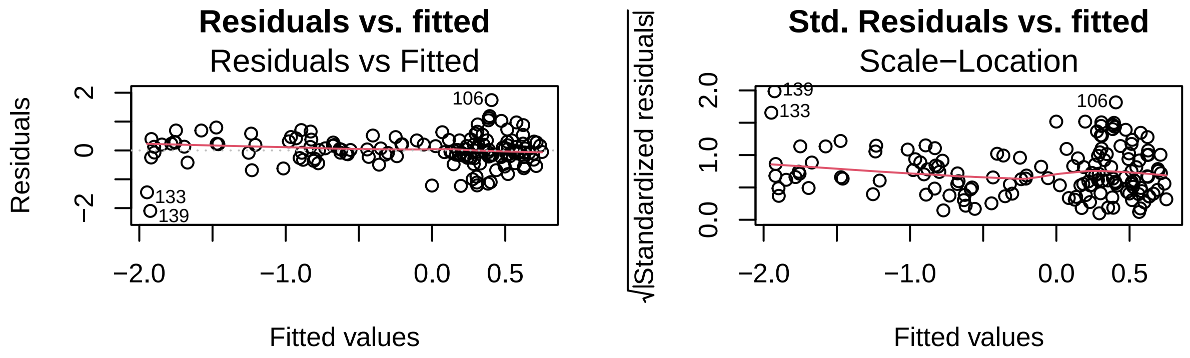

Outliers

```r
# check for outliers in top four models
# plot studentized residuals, cook's distance, and leverage
Percentchangea_stud.resid <- rstudent(Percentchange_model16)
par(mfrow = c(1, 3))
plot(Percentchangea_stud.resid, main = "Studentized residuals")
plot(Percentchange_model16, which = 4, main = "Cook's distance")
plot(Percentchange_model16, which = 5, main = "Leverage")
```

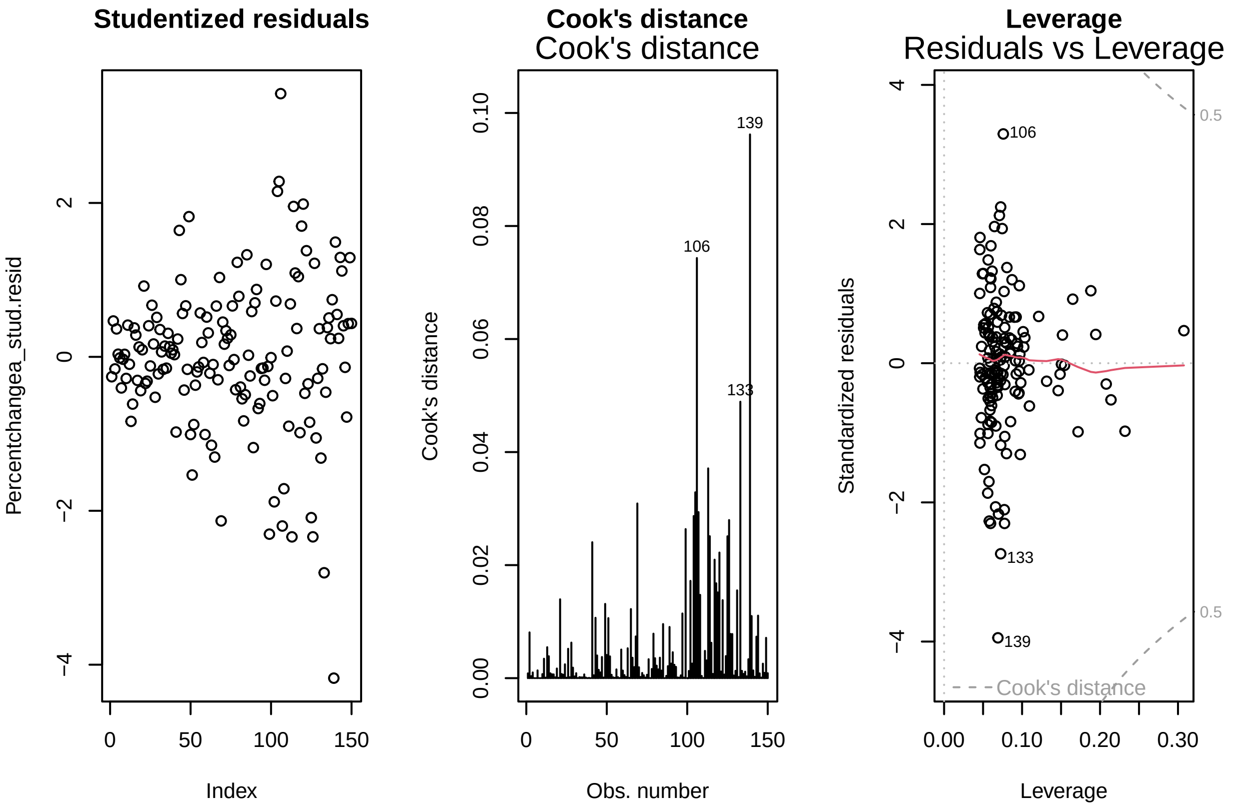

We chose to proceed with Percentchange_model16 based on the fact that it had the lowest AIC and best met the assumptions of homoscedasticity (based on residuals vs. fitted values, standardized residuals vs. fitted values) and had no significant outliers (studentized residuals between -3 and 3, cook's distances were below 1, and/or leverage was less than 0.5). Despite a violation of the normality assumption (based on q-q plots and Shapiro-Wilk test statistic), the model was deemed suitable for analysis due to its robustness to such deviations.

Model inference (Percentchange)

In this section, we inspected the values of the coefficients and the corresponding confidence intervals for body mass, treatment (1 = winter, 2 = summer, 3 = MHW), species (1= NL, 2 = AT, 3 = CS), the body mass:treatment interaction, and species:treatment interaction.

```r
# generate model summary and confidence interval
summary(Percentchange_model16)
```

```
##
## Call:
## lm(formula = Percentchange ~ Mass * Treatment + Treatment * Species,
##     data = Foraging)
```

```
##
## Residuals:
##     Min      1Q  Median      3Q     Max
## -2.0964 -0.2215  0.0132  0.2720  1.7438
##
## Coefficients:
##                      Estimate Std. Error t value Pr(>|t|)
## (Intercept)          0.660697   0.249738   2.646 0.009102 **
## Mass                -0.005145   0.002696  -1.908 0.058448 .
## Treatment2           0.260548   0.340698   0.765 0.445727
## Treatment3          -1.642132   0.323107  -5.082 1.19e-06 ***
## Species2             0.227386   0.202128   1.125 0.262559
## Species3            -0.185506   0.219490  -0.845 0.399478
## Mass:Treatment2     -0.008000   0.004820  -1.660 0.099268 .
## Mass:Treatment3      0.016024   0.004179   3.835 0.000191 ***
## Treatment2:Species2 -0.127321   0.291531  -0.437 0.662988
## Treatment3:Species2 -0.550823   0.280880  -1.961 0.051885 .
## Treatment2:Species3 -0.094931   0.300344  -0.316 0.752423
## Treatment3:Species3 -0.832656   0.291330  -2.858 0.004923 **
## ---
## Signif. codes:  0 '***' 0.001 '**' 0.01 '*' 0.05 '.' 0.1 ' ' 1
##
## Residual standard error: 0.5505 on 138 degrees of freedom
## Multiple R-squared:  0.6844, Adjusted R-squared:  0.6593
## F-statistic: 27.21 on 11 and 138 DF,  p-value: < 2.2e-16
```

```
confint(Percentchange_model16)
```

```
##                            2.5 %        97.5 %
## (Intercept)          0.166890108  1.1545047906
## Mass                -0.010475510  0.0001864179
## Treatment2          -0.413115267  0.9342114971
## Treatment3          -2.281013510 -1.0032505957
## Species2            -0.172282128  0.6270533501
## Species3            -0.619503859  0.2484911561
## Mass:Treatment2     -0.017530859  0.0015314681
## Mass:Treatment3      0.007761251  0.0242874853
## Treatment2:Species2 -0.703765717  0.4491237178
## Treatment3:Species2 -1.106207711  0.0045623409
## Treatment2:Species3 -0.688801632  0.4989390057
## Treatment3:Species3 -1.408704007 -0.2566089766
```

```
# create a forest plot for model coefficients and confidence intervals
Percentchange_forestplot_coef <- data.frame(
  Variable = rownames(summary(Percentchange_model16)$coefficients),
  Estimate = summary(Percentchange_model16)$coefficients[, 1],
  Lower = confint(Percentchange_model16)[, 1],
  Upper = confint(Percentchange_model16)[, 2])

ggplot(Percentchange_forestplot_coef, aes(x = Estimate, y = Variable)) +
  geom_errorbarh(aes(xmin = Lower, xmax = Upper), height = 0, color = "black") +
  geom_vline(xintercept = 0, linetype = "dotted", color = "black") +
  geom_point(size = 3, shape = 21, fill = "white", color = "black") +
  coord_cartesian(xlim = c(-3, 2)) +
  scale_y_discrete(labels = function(x) gsub(":", " - ", x)) +
```

```r
  theme_classic() +
  xlab("Coefficient Estimate") +
  ylab("") +
  ggtitle("Percentchange - Model coefficients")
```

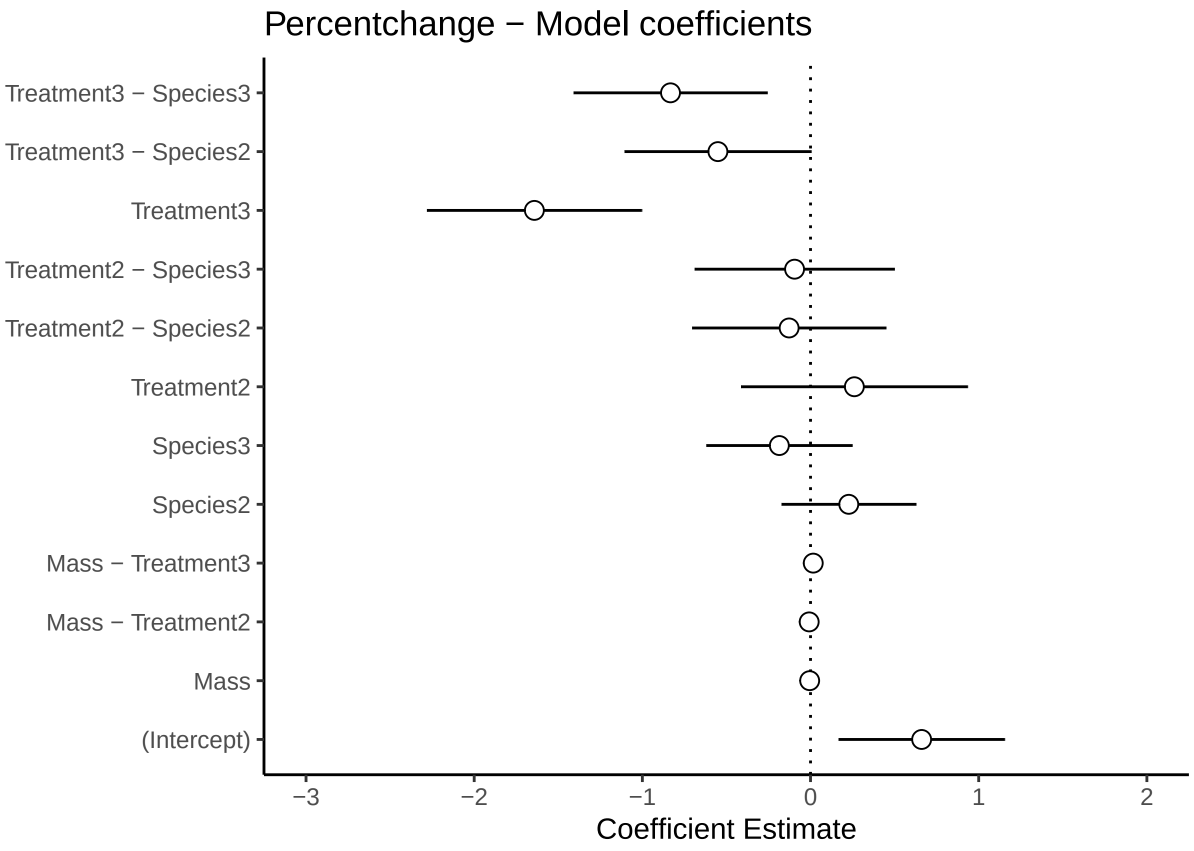


```r
# perform ANOVA
anova(Percentchange_model16)
```

```
## Analysis of Variance Table
##
## Response: Percentchange
##                     Df Sum Sq Mean Sq  F value    Pr(>F)
## Mass                 1  2.164   2.164   7.1384  0.008453 **
## Treatment            2 65.634  32.817 108.2762 < 2.2e-16 ***
## Species              2  8.070   4.035  13.3123 5.170e-06 ***
## Mass:Treatment       2 11.640   5.820  19.2025 4.389e-08 ***
## Treatment:Species    4  3.201   0.800   2.6400  0.036444 *
## Residuals          138 41.826   0.303
## ---
## Signif. codes:  0 '***' 0.001 '**' 0.01 '*' 0.05 '.' 0.1 ' ' 1
```

```r
# perform planned contrasts - treatment differences within species
Percentchange_NL_emmeans <- emmeans(Percentchange_model16, ~ Treatment, at = list(Mass = 53,
                                    Species = "1"))
Percentchange_NL_contrasts <- pairs(Percentchange_NL_emmeans, adjust = "none")
Percentchange_NL_summary <- summary(Percentchange_NL_contrasts)
Percentchange_NL_p_values <- Percentchange_NL_summary$p.value
Percentchange_NL_emmeans
```

```
##   Treatment emmean    SE  df lower.CL upper.CL
##   1           0.388 0.167 138   0.0588    0.717
##   2           0.225 0.145 138  -0.0622    0.511
##   3          -0.405 0.144 138  -0.6891   -0.121
##
## Confidence level used: 0.95
```

```
Percentchange_AT_emmeans <- emmeans(Percentchange_model16, ~ Treatment, at = list(Mass = 52,
                                    Species = "2"))
Percentchange_AT_contrasts <- pairs(Percentchange_AT_emmeans, adjust = "none")
Percentchange_AT_summary <- summary(Percentchange_AT_contrasts)
Percentchange_AT_p_values <- Percentchange_AT_summary$p.value
Percentchange_AT_emmeans
```

```
##   Treatment emmean    SE  df lower.CL upper.CL
##   1           0.621 0.118 138   0.3877    0.853
##   2           0.338 0.153 138   0.0359    0.640
##   3          -0.739 0.135 138  -1.0051   -0.473
##
## Confidence level used: 0.95
```

```
Percentchange_CS_emmeans <- emmeans(Percentchange_model16, ~ Treatment, at = list(Mass = 35,
                                   Species = "3"))
Percentchange_CS_contrasts <- pairs(Percentchange_CS_emmeans, adjust = "none")
Percentchange_CS_summary <- summary(Percentchange_CS_contrasts)
Percentchange_CS_p_values <- Percentchange_CS_summary$p.value
Percentchange_CS_emmeans
```

```
##   Treatment emmean    SE  df lower.CL upper.CL
##   1           0.295 0.131 138   0.0354    0.555
##   2           0.181 0.135 138  -0.0862    0.448
##   3          -1.619 0.121 138  -1.8571   -1.381
##
## Confidence level used: 0.95
```

```
# perform FDR correction on all contrasts
Percentchange_p_values <- c(Percentchange_NL_p_values, Percentchange_AT_p_values,
                            Percentchange_CS_p_values)
Percentchange_fdr_corrected_p <- p.adjust(Percentchange_p_values, method = "fdr")

Percentchange_contrast_names <- c("NL winter - NL summer","NL winter - NL MHW",
                                  "NL summer - NL MHW", "AT winter - AT summer",
                                  "AT winter - AT MHW","AT summer - AT MHW",
                                  "CS winter - CS summer","CS winter - CS MHW",
                                  "CS summer - CS MHW")
Percentchange_fdr_corrected_p_named <- setNames(Percentchange_fdr_corrected_p,
                                                Percentchange_contrast_names)
Percentchange_fdr_corrected_p_named
```

```
## NL winter - NL summer    NL winter - NL MHW    NL summer - NL MHW
##          5.180544e-01          7.863727e-04          3.722241e-03
## AT winter - AT summer    AT winter - AT MHW    AT summer - AT MHW
##          1.862281e-01          1.199354e-11          1.044949e-06
## CS winter - CS summer    CS winter - CS MHW    CS summer - CS MHW
##          5.447215e-01          5.675655e-19          3.014147e-17
```

```r
# forest plot of planned contrasts - treatment differences within species
Percentchange_create_forestplot <- function(data, plot_title = NULL, show_legend = FALSE) {
  data <- data %>%
    filter(Treatment %in% c(1, 2, 3))
  data$lower.CL <- data$emmean - 1.96 * data$SE
  data$upper.CL <- data$emmean + 1.96 * data$SE
  data$y_axis <- c(1, 2, 3)
  color_vector <- c("blue", "orange", "red")
  ggplot(data, aes(x = emmean, xmin = lower.CL, xmax = upper.CL, y = y_axis)) +
    geom_point(aes(color = factor(Treatment)), size = 4) +
    geom_errorbarh(aes(color = factor(Treatment)), height = 0) +
    scale_color_manual(values = color_vector, name = "Treatment", labels =
    c("Winter","Summer","MHW")) +
    scale_y_continuous(breaks = c(1, 2, 3), labels = c("Winter", "Summer", "MHW")) +
    labs(title = plot_title, x = "Body mass change (%)", y = "") +
    theme_classic() +
    theme(legend.position = ifelse(show_legend, "right", "none")) +
    coord_cartesian(xlim = c(-2, 1))}

Percentchange_NL_forestplot_emmean <- Percentchange_create_forestplot(data.frame
                                      (Percentchange_NL_emmeans), plot_title="NL")
Percentchange_AT_forestplot_emmean <- Percentchange_create_forestplot(data.frame
                                      (Percentchange_AT_emmeans), plot_title="AT")
Percentchange_CS_forestplot_emmean <- Percentchange_create_forestplot(data.frame
                                      (Percentchange_CS_emmeans), plot_title="CS")
plot_grid(Percentchange_NL_forestplot_emmean, Percentchange_AT_forestplot_emmean,
          Percentchange_CS_forestplot_emmean, nrow = 1, rel_widths = c(0.33, 0.33, 0.33))
```

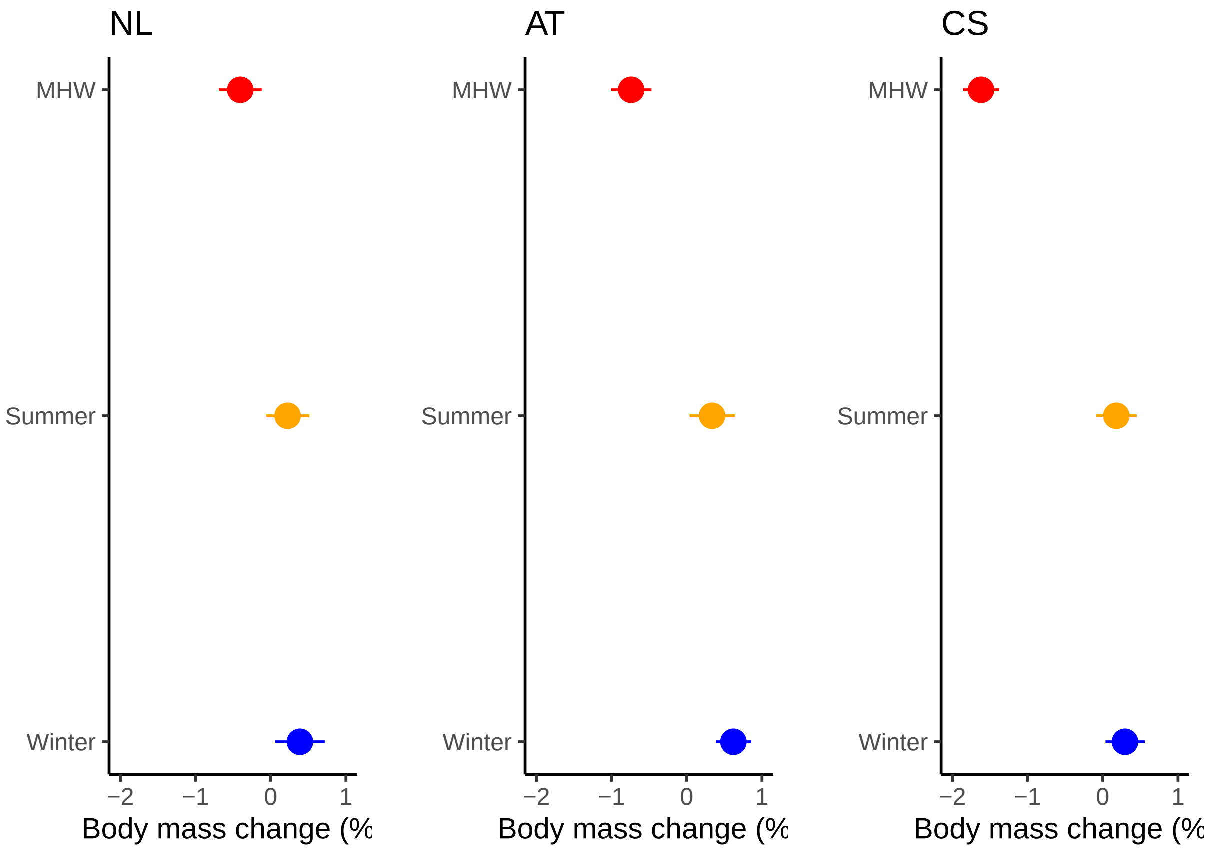

The most parsimonious linear regression model (Percentchange_model16) statistically significantly predicted percent changes in body mass (F11,138=27.210, p<0.001, adj. R2=0.659). The interaction between body mass and treatment (F2,138=19.203, p<0.001) and the interaction between treatment and species (F4,138=2.640, p=0.036) added significantly to the prediction of percent changes in body mass. With regards to our hypothesis, model coefficients and/or planned contrasts revealed that percent changes in body mass did not significantly differ between winter and summer, while it decreased during a MHW.

Model predictions (Percentchange)

In this section, we use the model to predict mean Percentchange with confidence intervals for all species across their respective body mass ranges.

```
# predicting Percentchange (mean and confidence intervals)
# find the minimum and maximum body mass values for AT and NL in the dataset
nl_min_mass <- 14
nl_max_mass <- 168
at_min_mass <- 27
at_max_mass <- 102
cs_min_mass <- 5
cs_max_mass <- 80

# create a data frame with the desired body mass values for prediction
Percentchange_nl_new_predictors <- data.frame(
  Species = '1',
  Mass = rep(seq(nl_min_mass, nl_max_mass, length.out = 20), times = 3),
  Treatment = factor(rep(c(1, 2, 3), each = 20)))
Percentchange_at_new_predictors <- data.frame(
  Species = '2',
```

```r
  Mass = rep(seq(at_min_mass, at_max_mass, length.out = 20), times = 3),
  Treatment = factor(rep(c(1, 2, 3), each = 20)))
Percentchange_cs_new_predictors <- data.frame(
  Species = '3',
  Mass = rep(seq(cs_min_mass, cs_max_mass, length.out = 20), times = 3),
  Treatment = factor(rep(c(1, 2, 3), each = 20)))

# use predict() to obtain predictions for each treatment level
Percentchange_nl_predictions <- predict(Percentchange_model16, newdata =
                                Percentchange_nl_new_predictors, interval = "confidence")
Percentchange_at_predictions <- predict(Percentchange_model16, newdata =
                                Percentchange_at_new_predictors, interval = "confidence")
Percentchange_cs_predictions <- predict(Percentchange_model16, newdata =
                                Percentchange_cs_new_predictors, interval = "confidence")

# add the predicted Percentchange values to the data frame
Percentchange_nl_new_predictors$Percentchange <- Percentchange_nl_predictions[,1]
Percentchange_nl_new_predictors$lower <- Percentchange_nl_predictions[,2]
Percentchange_nl_new_predictors$upper <- Percentchange_nl_predictions[,3]
Percentchange_at_new_predictors$Percentchange <- Percentchange_at_predictions[,1]
Percentchange_at_new_predictors$lower <- Percentchange_at_predictions[,2]
Percentchange_at_new_predictors$upper <- Percentchange_at_predictions[,3]
Percentchange_cs_new_predictors$Percentchange <- Percentchange_cs_predictions[,1]
Percentchange_cs_new_predictors$lower <- Percentchange_cs_predictions[,2]
Percentchange_cs_new_predictors$upper <- Percentchange_cs_predictions[,3]

# combine the predictions with the new predictors
Percentchange_nl_predicted <- cbind(Percentchange_nl_new_predictors, Percentchange_nl_predictions)
Percentchange_at_predicted <- cbind(Percentchange_at_new_predictors, Percentchange_at_predictions)
Percentchange_cs_predicted <- cbind(Percentchange_cs_new_predictors, Percentchange_cs_predictions)

# plot the data
Percentchange_NL_predictplot <- ggplot() +
  geom_ribbon(data = Percentchange_nl_predicted, aes(x = Mass, y = Percentchange, ymin = lwr,
  ymax = upr, fill = Treatment), alpha = 0.2) +
  geom_line(data = Percentchange_nl_predicted, aes(x = Mass, y = Percentchange, color =
  Treatment), linewidth = 1) +
  geom_point(data = Foraging[Foraging$Species == '1',], aes(x = Mass, y = Percentchange,
  color = Treatment), size = 2) +
  ggtitle("NL") +
  scale_fill_manual(values = Treatment_colors, guide = "none") +
  scale_color_manual(values = Treatment_colors, labels = Treatment_labels) +
  labs(x = "Mass (g)", y = "Daily body mass change (%)") +
  theme(legend.position = "none") +
  coord_cartesian(xlim = c(0, 150), ylim = c(-4, 2))

Percentchange_AT_predictplot <- ggplot() +
  geom_ribbon(data = Percentchange_at_predicted, aes(x = Mass, y = Percentchange, ymin = lwr,
  ymax = upr, fill = Treatment), alpha = 0.2) +
  geom_line(data = Percentchange_at_predicted, aes(x = Mass, y = Percentchange, color =
  Treatment), linewidth = 1) +
  geom_point(data = Foraging[Foraging$Species == '2',], aes(x = Mass, y = Percentchange,
  color = Treatment), size = 2) +
```

```
  ggtitle("AT") +
  scale_fill_manual(values = Treatment_colors, guide = "none") +
  scale_color_manual(values = Treatment_colors, labels = Treatment_labels) +
  labs(x = "Mass (g)", y = "Daily body mass change (%)") +
  theme(legend.position = "none") +
  coord_cartesian(xlim = c(0, 150), ylim = c(-4, 2))

Percentchange_CS_predictplot <- ggplot() +
  geom_ribbon(data = Percentchange_cs_predicted, aes(x = Mass, y = Percentchange, ymin = lwr,
  ymax = upr, fill = Treatment), alpha = 0.2) +
  geom_line(data = Percentchange_cs_predicted, aes(x = Mass, y = Percentchange, color =
  Treatment), linewidth = 1) +
  geom_point(data = Foraging[Foraging$Species == '3',], aes(x = Mass, y = Percentchange,
  color = Treatment), size = 2) +
  ggtitle("CS") +
  scale_fill_manual(values = Treatment_colors, guide = "none") +
  scale_color_manual(values = Treatment_colors, labels = Treatment_labels) +
  labs(x = "Mass (g)", y = "Daily body mass change (%)") +
  theme(legend.position = "right") +
  coord_cartesian(xlim = c(0, 150), ylim = c(-4, 2))

plot_grid(Percentchange_NL_predictplot, Percentchange_AT_predictplot, Percentchange_CS_predictplot, nro
```

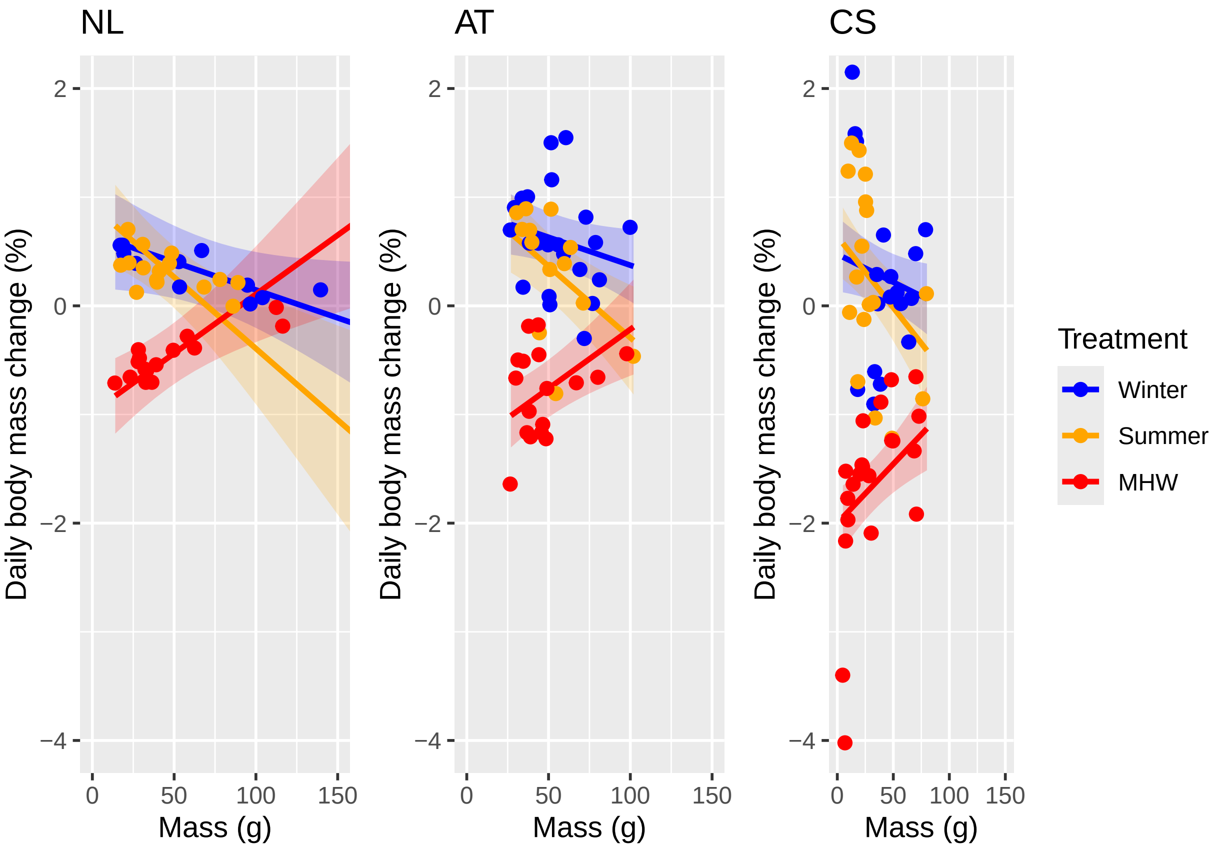

Supplement: Supplementary file 4 — Data S4: Supporting Information 4. [file GCB-31-e70438-s001.pdf]
